# Supplementary figures and images for: Disrupted rhythms of life, work and entertainment and their associations with psychological impacts under the stress of the COVID-19 pandemic: A survey in 5854 Chinese people with different sociodemographic backgrounds
Source: PLoS One. 2021 May 17;16(5):e0250770. doi: 10.1371/journal.pone.0250770 (PMC8128272; doi:10.1371/journal.pone.0250770)

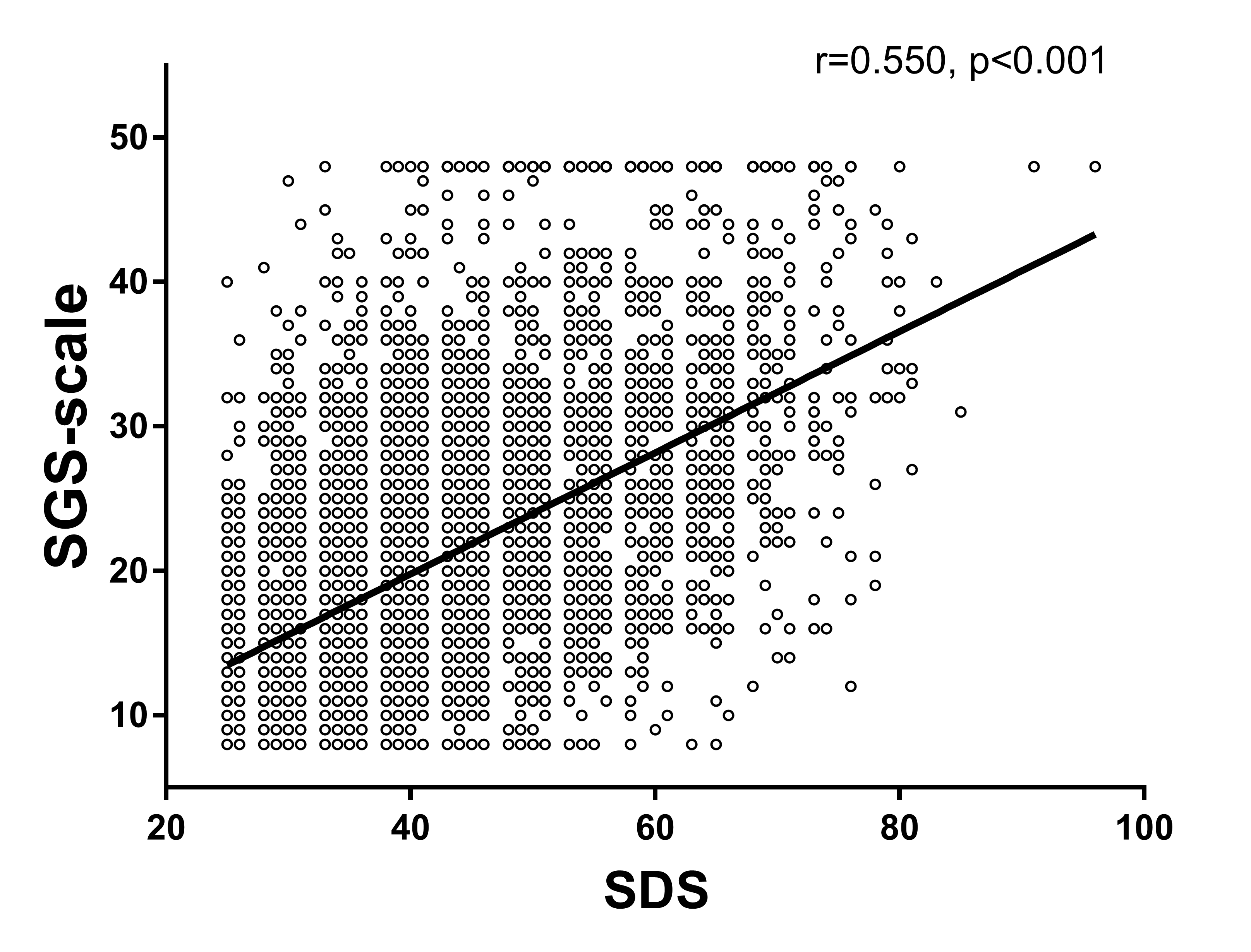

Supplement: S1 Fig — (TIF) [file pone.0250770.s001.tif]

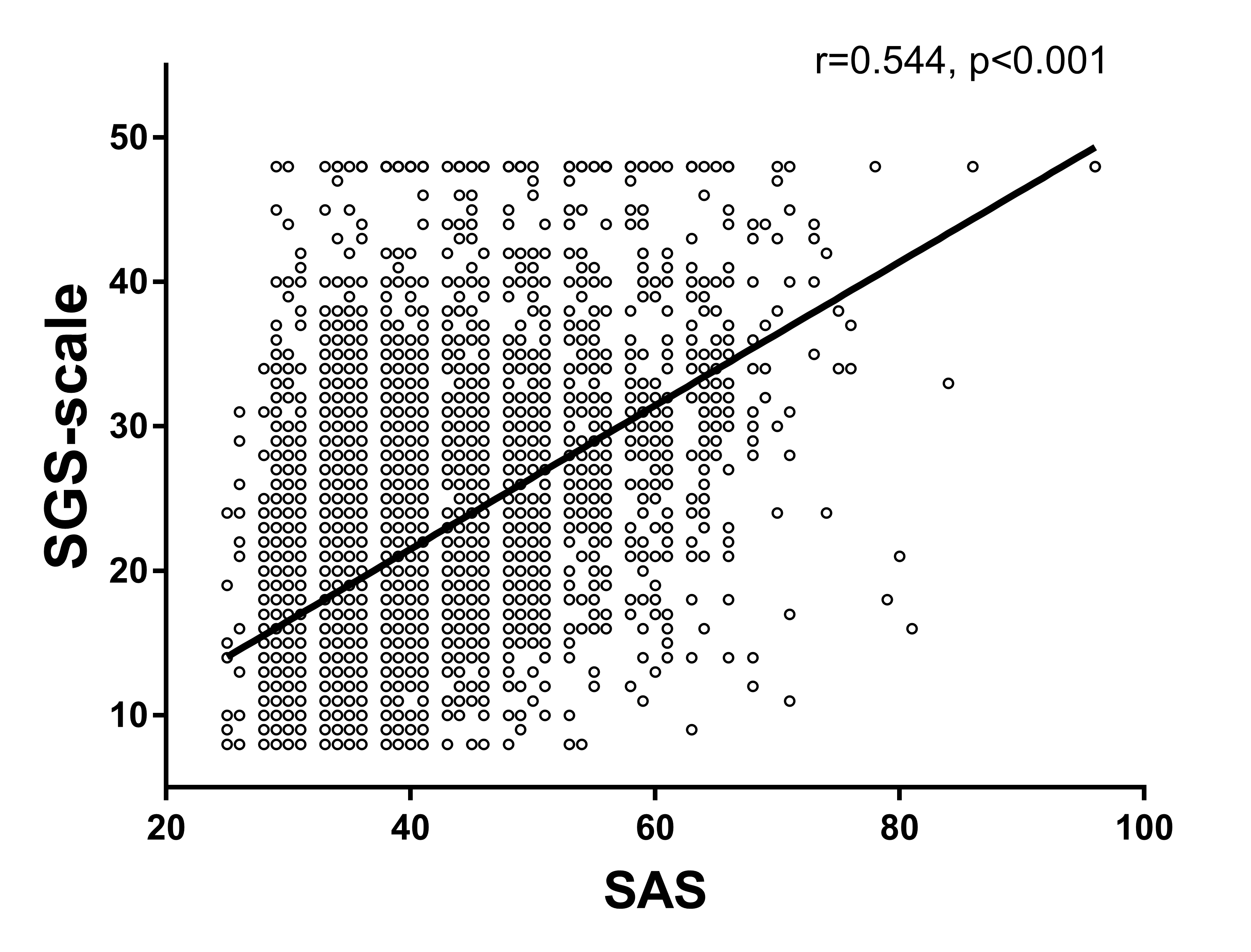

Supplement: S2 Fig — (TIF) [file pone.0250770.s002.tif]
