# Supplementary material for: Disrupted rhythms of life, work and entertainment and their associations with psychological impacts under the stress of the COVID-19 pandemic: A survey in 5854 Chinese people with different sociodemographic backgrounds
Source: PLoS One. 2021 May 17;16(5):e0250770. doi: 10.1371/journal.pone.0250770 (PMC8128272; doi:10.1371/journal.pone.0250770)
Supplement: S1 Table — (DOCX) [file pone.0250770.s004.docx]

**S1 Table. Prevalence of depression with different grades in participants (N=5854).**

|  | **N** | **Normal** | **Mild** | **Moderate** | **Severe** | ***P*** |
| --- | --- | --- | --- | --- | --- | --- |
|  |  | **N=4430** | **N=897** | **N=402** | **N=125** |  |
| **Health status** | | | | | | |
| Poor | 53 | 6(11.32%) | 16(30.19%) | 16(30.19%) | 15(28.30%) | <0.001 |
| Normal | 787 | 410(52.10%) | 204(25.92%) | 121(15.37%) | 52(6.61%) |  |
| Good | 3686 | 2849(77.29%) | 576(15.63%) | 218(5.91%) | 43(1.17%) |  |
| Very good | 1328 | 1165(87.73%) | 101(7.61%) | 47(3.54%) | 15(1.13%) |  |
| **Current occupation** | | | | | | |
| Businessman | 481 | 388(80.67%) | 64(13.31%) | 25(5.20%) | 4(0.83%) | <0.001 |
| Officer | 245 | 201(82.04%) | 39(15.92%) | 3(1.22%) | 2(0.82%) |  |
| Teacher | 231 | 183(79.22%) | 35(15.15%) | 5(2.16%) | 8(3.46%) |  |
| Police | 277 | 234(84.48%) | 25(9.03%) | 15(5.42%) | 3(1.08%) |  |
| Farmer | 217 | 159(73.27%) | 39(17.97%) | 14(6.45%) | 5(2.30%) |  |
| Employee | 270 | 208(77.04%) | 47(17.41%) | 12(4.44%) | 3(1.11%) |  |
| Doctor | 1171 | 828(70.71%) | 188(16.05%) | 115(9.82%) | 40(3.42%) |  |
| Nurse | 1017 | 682(67.06%) | 186(18.29%) | 113(11.11%) | 36(3.54%) |  |
| Medical technician | 139 | 100(71.94%) | 23(16.55%) | 10(7.19%) | 6(4.32%) |  |
| Retired re-employee | 229 | 174(75.98%) | 42(18.34%) | 9(3.93%) | 4(1.75%) |  |
| Non-medical student | 1172 | 984(83.96%) | 131(11.18%) | 49(4.18%) | 8(0.68%) |  |
| Medical student | 405 | 289(71.36%) | 78(19.26%) | 32(7.90%) | 6(1.48%) |  |
| **Chronic disease** | | | | | | |
| No chronic disease | 4373 | 3519(80.47%) | 566(12.94%) | 233(5.33%) | 55(1.26%) | <0.001 |
| CDCPD | 671 | 363(54.10%) | 171(25.48%) | 90(13.41%) | 47(7.00%) |  |
| Chronic diseases only | 810 | 548(67.65%) | 160(19.75%) | 79(9.75%) | 23(2.84%) |  |

SDS, Zung's self-rating depression scale; CY, Chinese yuan; CDCPD, Chronic diseases comorbid with psychosomatic diseases.
