# Supplementary material for: Disrupted rhythms of life, work and entertainment and their associations with psychological impacts under the stress of the COVID-19 pandemic: A survey in 5854 Chinese people with different sociodemographic backgrounds
Source: PLoS One. 2021 May 17;16(5):e0250770. doi: 10.1371/journal.pone.0250770 (PMC8128272; doi:10.1371/journal.pone.0250770)
Supplement: S2 Table — (DOCX) [file pone.0250770.s005.docx]

**S2 Table. Prevalence of anxiety with different grades in participants (N=5854).**

|  | **N** | **Normal** | **Mild** | **Moderate and severe** | ***P*** |
| --- | --- | --- | --- | --- | --- |
|  |  | **N=5114** | **N=513** | **N=227** |  |
| **Health status** | | | | | |
| Poor | 53 | 17(32.08%) | 18(33.96%) | 18(33.96%) | <0.001 |
| Normal | 787 | 550(69.89%) | 161(20.46%) | 76(9.66%) |  |
| Good | 3686 | 3297(89.45%) | 287(7.79%) | 102(2.77%) |  |
| Very good | 1328 | 1250(94.13%) | 47(3.54%) | 31(2.33%) |  |
| **Current occupation** | | | | | |
| Businessman | 481 | 438(91.06%) | 31(6.44%) | 12(2.49%) | <0.001 |
| Officer | 245 | 228(93.06%) | 14(5.71%) | 3(1.22%) |  |
| Teacher | 231 | 203(87.88%) | 21(9.09%) | 7(3.03%) |  |
| Police | 277 | 252(90.97%) | 19(6.86%) | 6(2.17%) |  |
| Farmer | 217 | 195(89.86%) | 14(6.45%) | 8(3.69%) |  |
| Employee | 270 | 240(88.89%) | 25(9.26%) | 5(1.85%) |  |
| Doctor | 1171 | 961(82.07%) | 130(11.10%) | 80(6.83%) |  |
| Nurse | 1017 | 824(81.02%) | 129(12.68%) | 64(6.29%) |  |
| Medical technician | 139 | 119(85.61%) | 13(9.35%) | 7(5.04%) |  |
| Retired re-employee | 229 | 200(87.34%) | 23(10.04%) | 6(2.62%) |  |
| Non-medical student | 1172 | 1105(94.28%) | 53(4.52%) | 14(1.19%) |  |
| Medical student | 405 | 349(86.17%) | 41(10.12%) | 15(3.70%) |  |
| **Chronic disease** | | | | | |
| No chronic disease | 4373 | 4004(91.56%) | 262(5.99%) | 107(2.45%) | <0.001 |
| CDCPD | 671 | 460(68.55%) | 136(20.27%) | 75(11.18%) |  |
| Chronic diseases only | 810 | 650(80.25%) | 115(14.20%) | 45(5.56%) |  |

SAS, Zung's self-rating anxiety scale; CY, Chinese yuan; CDCPD, Chronic diseases comorbid with psychosomatic diseases.
