# Supplementary material for: Disrupted rhythms of life, work and entertainment and their associations with psychological impacts under the stress of the COVID-19 pandemic: A survey in 5854 Chinese people with different sociodemographic backgrounds
Source: PLoS One. 2021 May 17;16(5):e0250770. doi: 10.1371/journal.pone.0250770 (PMC8128272; doi:10.1371/journal.pone.0250770)
Supplement: S3 Table — (N = 5854). (DOCX) [file pone.0250770.s006.docx]

**S3 Table. Spearman’s correlation between subscale 1, subscale 2, Zung's self-rating depression scale (SDS)and Zung's self-rating anxiety scale (SAS). (N=5854)**

| **Outcomes** | **Subscale1** | **Subscale2** | **SDS** | **SAS** |
| --- | --- | --- | --- | --- |
| **Subscale1** | - | 0.058** | 0.550** | 0.544** |
| **Subscale2** | - | - | -0.037** | 0.008 |
| **SDS** | - | - | - | 0.808** |
| **SAS** | - | - | - | - |

**, *P*<0.001.
