# Supplementary material for: Disrupted rhythms of life, work and entertainment and their associations with psychological impacts under the stress of the COVID-19 pandemic: A survey in 5854 Chinese people with different sociodemographic backgrounds
Source: PLoS One. 2021 May 17;16(5):e0250770. doi: 10.1371/journal.pone.0250770 (PMC8128272; doi:10.1371/journal.pone.0250770)
Supplement: Attachment — Submitted filename: PONE-D-20-26146_R1.pdf [file pone.0250770.s009.pdf]

PLOS ONE

Disrupted rhythms of life, work and entertainment and their associations with psychological impacts under the stress of the COVID-19 pandemic: A Survey in 5854 Chinese people with different sociodemographic backgrounds

--Manuscript Draft--

**Manuscript Number:**	PONE-D-20-26146R1
**Article Type:**	Research Article
**Full Title:**	Disrupted rhythms of life, work and entertainment and their associations with psychological impacts under the stress of the COVID-19 pandemic: A Survey in 5854 Chinese people with different sociodemographic backgrounds
**Short Title:**	Disrupted rhythms and their associations with mental health problems under the stress of COVID-19 Epidemic.
**Corresponding Author:**	Dongfeng Chen<br>Army Medical Center of PLA, Daping Hospital, Army Medical University, No.10 Changjiang Branch Road, Daping, Yuzhong District, Chongqing 400042,P.R.China<br>Chongqing, CHINA
**Keywords:**	Coronavirus disease 2019 (COVID-19); Social rhythm disruption; Anxiety and depression; Psychological impacts; Chinese people.
**Abstract:**	Background & Aim The coronavirus disease 2019 (COVID-19) pandemic has affected the life and work of people worldwide. The present study aimed to evaluate the rhythm disruptions of life, work, and entertainment, and their associations with the psychological impacts during the initial phase of the COVID-19 pandemic.<br><br>Method A cross-sectional study was conducted from the 10 th to 17 th March 2020 in China. A structured e-questionnaire containing general information, the Chinese version of Brief Social Rhythm Scale, and Zung's self-rating scales of depression and anxiety (SDS and SAS) was posted and collected online through a public media (i.e. EQxiu online questionnaire platform). Scores in sleeping, getting up, and socializing (SGS) rhythm and eating, physical practice, and entertainment (EPE) rhythm were compared among and between participants with different sociodemographic backgrounds including gender, age, education, current occupation, annual income, health status, and chronic disease status. Correlations of SDS and SAS with SGS-scale and EPE-scale were analyzed.<br><br>Results Overall, 5854 participants were included. There were significant differences in the scores of SGS-scale and EPE-scale among people with different sociodemographic backgrounds. The scores were significantly higher in the groups with female gender, low education level, too-low or too-high income, poor health status, ages of 26-30 years or older than 61 years, nurses and subjects with divorce or widow status. There were also significant differences in SAS and SDS scores among people with different sociodemographic backgrounds (all $P < 0.05$). The overall prevalence of depression and anxiety was 24.3% and 12.6%, respectively, with nurses having the highest rates of depression (32.94%) and anxiety (18.98%) among the different occupational groups. SGS-scale was moderately correlated with SDS and SAS, and disruption of SGS rhythm was an independent risk factor for depression and anxiety.<br><br>Conclusion Social rhythm disruption was independently associated with depression and anxiety. Interventions should be applied to people vulnerable to the rhythm disruption during the COVID-19 pandemic.
**Order of Authors:**	Min Yang
	Ping He
	Xiaoming Xu
	Dan Li
	Jing Wang

Powered by Editorial Manager® and ProduXion Manager® from Aries Systems Corporation

	Yanjun Wang
	Bin Wang
	Wo Wang
	Mei Zhao
	Hui Lin
	Mingming Deng
	Tianwei Deng
	Li Kuang
	Dongfeng Chen
**Opposed Reviewers:**	
**Response to Reviewers:**	Dear Dr. Bullock,<br>Thank you for your decision letter and the reviewers for their time in reviewing our manuscript (ID: PONE-D-20-26146), entitled “Disrupted rhythms of life, work and entertainment and their associations with psychological impacts under the stress of the COVID-19 pandemic: A Survey in 5854 Chinese people with different sociodemographic backgrounds”. According to your insightful advice and the thoughtful comments and suggestions of the reviewers, we have significantly revised our manuscript. All changes are highlighted in red in the revised manuscript. In addition, we have responded to the reviewers’ comments in a point-by-point format, which are listed below this letter. We would like to re-submit it for your consideration. We hope that the revision is acceptable, and we look forward to hearing from you soon.<br><br>Response to the comments of Reviewer #1:<br>1. This is a timely and novel research project.<br>Overall language - While there is a very good flow of idea and structure to the manuscript, the language is awkward and redundant at times, and would benefit from additional editing by a fluent or native speaker of English.<br>Response: Thanks for your positive and encouraging comment on our research project. According to your suggestion, we have sent our manuscript to Medjaden Inc, a professional editing and proofreading service, for language polishing.<br><br>2. Statistical analysis - Please considering re-calculating probabilities while correcting for multiple comparisons/tests.<br>Response: Thanks for your professional suggestion. Accordingly, we have re-calculated probabilities while correcting for multiple comparisons. For more clarity and readability, we have split the original Table 1 into four tables, i.e. Table 1 (General sociodemographic characteristics of 5854 analyzed participants) (Page 9, Line 15 — Page 10, Line 1); Table 2 (Scores of social rhythms in participants with different sociodemographic backgrounds during the COVID-19 pandemic (N=5854) (Page 11, Line 6 — Page 12, Line 3); Table 3 (Comparison of SGS-scale and EPE-scale scores between the group with the highest score and each of other groups in terms of sociodemographic backgrounds) (Page 12, Line 4 — Page 14, Line 3); Table 4 (Score of depression and anxiety in participants with different sociodemographic backgrounds during the COVID-19 pandemic, N=5854) (Page 15, Line 4 — Page 16, Line 3). The overall 2, Z and P-values in the multiple comparisons are shown in Table 2. Additionally, we have conducted comparisons between two groups for each variable and presented the results for the comparisons between the group with the highest score and each of other groups in SGS-scale (Table 3) (Page 12, Line 4 — Page 14, Line 3).<br><br>3. Please consider reducing the table content to improve readability.<br>Response: As described above, we have split the original Table 1 into four tables (Tables 1-4) for clarity, and deleted the columns of SAS and SDS in Table 1 in the revised manuscript. We have labeled the original Tables 2, 3 and 4 as Supplementary Tables 1 and 2, and 3 in the revised manuscript (Page 38-40). In addition, we have combined the original Tables 5 and 6 into a single table (Table 5) in the revised manuscript (Page 17, Line 10—Page 18, Line 3). Thus, the formal table number has been reduced from 6 to 5, with three complementary tables in the revised manuscript.

Powered by Editorial Manager® and ProduXion Manager® from Aries Systems Corporation

In addition, we have labeled the original Figures 1 and 2 as Supplementary Figures 1A &B in the revised manuscript (Page 37).

Responses to the comments of Reviewer #2:
1. Is the manuscript technically sound, and do the data support the conclusions? See comments on the discussion
Response: Thanks for your positive and valuable comments. Our responses are listed below your comments on the Discussion accordingly. Moreover, we have significantly revised the Discussion section of the revised manuscript (Page 19, Lines 11-20; Page 21, Lines 6-21; Page 22, Lines 1- 21; Page 23, Lines 1-21 and Page 24, Lines 1-4).
2. Has the statistical analysis been performed appropriately and rigorously? No revisions
Response: Thanks for your professional suggestion. Accordingly, we have re-calculated probabilities while correcting for multiple comparisons. For more clarity and readability, we have split the original Table 1 into four tables, i.e. Table 1 (General sociodemographic characteristics of 5854 analyzed participants) (Page 9, Line 15 — Page 10, Line 1); Table 2 (Scores of social rhythms in participants with different sociodemographic backgrounds during the COVID-19 pandemic (N=5854) (Page 11, Line 6 — Page 12, Line 3); Table 3 (Comparison of SGS-scale and EPE-scale scores between the group with the highest score and each of other groups in terms of sociodemographic backgrounds) (Page 12, Line 4 — Page 14, Line 3); Table 4 (Score of depression and anxiety in participants with different sociodemographic backgrounds during the COVID-19 pandemic, N=5854) (Page 15, Line 4 — Page 16, Line 3). The overall 2, Z and P-values in the multiple comparisons are shown in Table 2. Additionally, we have conducted comparisons between two groups for each variable and presented the results for the comparisons between the group with the highest score and each of other groups in SGS-scale (Table 3) (Page 12, Line 4 — Page 14, Line 3).

3. Have the authors made all data underlying the findings in their manuscript fully available? No revisions.
Response: Thanks for your professional suggestion. We have made all data underlying the findings in the revised manuscript fully available. According to your insightful advice, these data have been reflected in the revised version of the Results, Discussions, and Supplementary materials.

4. Is the manuscript written in an intelligible fashion and written in standard English? While I answered yes, I think this manuscript calls for improvement and revisions in this category before publication. For example in the abstract the background and objective are awkward and hard to follow. As is, they read: “The rhythms of life, work and entertainment behaviors are considered as the external behavioral manifestations of biological rhythm. The present study aimed to evaluate the distinctive disrupted rhythms of behaviors and their associations with mental health problems in people with different backgrounds under the stress of COVID19 pandemic.” Social rhythms such as work and entertainment routines are driven by biological rhythms including the sleep wake cycle. The present study aimed to evaluate if disruption of social rhythms and routines were associated with mental health problems under the stress of the COVID19 pandemic and if this differed by people of XXX.”
Response: Thanks for your good suggestion. As for manuscript writing and language, we have tried our best to revise and improve the manuscript. In addition, we have sent our revised manuscript to Medjaden Inc, a professional editing and proofreading service, for language polishing.

Additional comments:
Introduction:
1. The Introduction needs to be expanded and to include more of the relevant literature. You seem to be missing a large body of research on this topic. Please see the article by Murray, Gottleib, & Swartz (2020) Maintaining Daily Routines to Stabilize Mood: Theory, Data, and Potential Intervention for Circadian Consequences of COVID-19. Canadian Journal of Psychiatry. If you look at the reference list of this paper, you will find more literature on this topic not included in your Background section.
Response: Thanks for your professional suggestion, and providing the valuable article, which we have learned and cited in the Introduction and Discussion sections of the revised manuscript (Page 5, Lines 13-14; Page 22, Lines 14-21; Page 23, Lines 1-9;

Page 23, Lines 9-21 and Page 24, Lines 1-4). Accordingly, we have made revisions with more literature on this topic in the Introduction section of the revised manuscript (Reference 5, 7, 23, and 24).

2. Specify what groups you are comparing in your aims.
Response: Thanks for your professional suggestion. The aim of the present study was to evaluate the social rhythm and its associations with the psychological impacts during the initial phase of the COVID-19 pandemic among Chinese people with different sociodemographic backgrounds including gender, age, marital status, education level, annual income, health status, current occupation, and chronic disease status. Moreover, we also compared the group with the highest SGS-scale and EPE-scale scores with each of other groups in terms of the above-mentioned variables (Table 3) (Page 12, Line 4 — Page 14, Line 3). Special groups referred to nurses, the elderly and subjects being divorced or with chronic diseases or psychosomatic diseases who were mostly suffered from disrupted rhythms of life-work behaviors. This point has been clarified in the revised manuscript (Page 10, Lines 7-14; Page 11, Lines 1-5).

3. Is this exploratory or do you have hypotheses?
Response: Yes, we have a hypothesis. We hypothesize that people with different sociodemographic backgrounds have different degrees of rhythm disruption in life, work, and entertainment, that the rhythm disruption is more severe in some specific groups, and that the rhythm disruption is associated with psychological impacts during the initial phase of the COVID-19 pandemic. Accordingly, we have stated the hypotheses clearly in the Introduction of the revised manuscript (Page 6, Lines 4-8).

Methods:
1. In the limitations I would include that those under 18, at least those over 13 could have signed/checked their own assent in addition to their parents' consent.
Response: Thanks for your thoughtful suggestion. Indeed, we did not consider obtaining the individual informed consent for teenagers themselves, in addition to their parents' consent. According to your suggestion, we have added it as a limitation in the Discussion section of the revised manuscript (Page 25, Line 21 and Page 26 Lines 1-2).

2. I suggest giving brief information on the psychometric properties for your instruments.
Response: Thanks for your professional suggestion. Accordingly, we have briefly described the psychometric properties for the instruments used in our study with references to the initial version and the Chinese version of SAS and SDS to interpret their reliability and validity in the revised manuscript (Zung's Depression and Anxiety Scale, Page 8, Lines 11-14).

3. Did you track ask about circadian rhythms/sleep wake cycle? If not, this is a limitation.
Response: Thanks for your thoughtful suggestion. In this study, we did not track the 24-hour circadian rhythm. Indeed, it is necessary to track the circadian rhythms/sleep wake cycle by multilead-sleep monitor considering different psychological impacts, which will be implemented in our further research. According to your suggestion, we have added this point as a limitation in the Discussion section of the revised manuscript (Page 25, Lines 15-18).

Statistical Analysis:
Specify what groups you are comparing. If a one-way t-test was used specify your hypothesis after your aims.
Response: Thanks for your professional suggestion. In the present study, SGS-scale and EPE-scale scores reflecting the life-work and entertainment activities, respectively were compared among Chinese people with different sociodemographic backgrounds including gender, age, marital status, education level, annual income, health status, current occupation, and chronic disease status. Moreover, the group with the highest score was compared with each of other groups in terms of above-mentioned variables by using one-way ANOVA with a post hoc test (Table 3) (Page 12, Line 4 — Page 14, Line 3). This point has been clearly stated in the Statistical analysis of the revised manuscript (Page 8, Lines 18-19). In addition, we have specified our hypothesis after the aim (Page 6, Lines 4-8).

Powered by Editorial Manager® and ProduXion Manager® from Aries Systems Corporation

Results:
1. What was the response rate?
Response: Thanks for your pertinent question. It was challenging to get the survey response rate because the lockdown and social isolation policy did not encourage a face-to-face survey. Therefore, we conducted the present study using online-survey through a public media (i.e. EQxiu online questionnaire platform), to which participation was voluntary. Accordingly, we have added this point as a limitation in the Discussion section of the revised manuscript (Page 25, Lines 18-21).

2. Give an example of why some questionnaires were invalid and therefore excluded.
Response: Eighteen questionnaires with invalid were excluded the data analysis due to frivolous or incomplete answers, or the same option for different questions or orderly answers. The information on the invalid questionnaires have been clearly presented in the Results section of the revised manuscript (Page 9, Lines 10-12).

3. In section 4 (Correlation analysis) specify what you mean by low score and high score group the first time you say it. Also, specify what the subscales are instead of referring to them as subscale1, etc.
Response: Thanks for carefully reviewing the manuscript and raising the suggestions. We have deleted the classification of "low score and high score groups". For more readability, we have renamed the two subscales of the social rhythm scale (i.e. subscale1 and subscale2) as SGS-scale and EPE-scale, respectively, in the revised manuscript. Specifically, the first eight items constituted SGS-scale, which addresses sleeping, getting up, and socializing rhythm, and the last nine items constituted the EPE-scale, which addresses eating, physical practice, and entertainment rhythm. These terms have been clearly defined in the Measures section of the revised manuscript (Page 7, Lines 16-20).

Discussion:
There seems to be a lot of speculation in this section and assumptions that is not backed up by research, the literature or the data. You may want to back up your paper/assertions with theory. See: Social Rhythms Disruption Theory.
Response: Thanks for your critical and constructive comment and suggestion. Accordingly, we have performed literature search and learned several theories on social rhythm, such as Social Zeitgeber Theory. Accordingly, we have extensively revised the Discussion section of the revised manuscript (Page 22, Lines 13-21; Page 23, Lines 1-21; Page 24, Lines 1-4).
There are five parts (paragraphs) in the Discussion section of the revised manuscript, including: 1), the characteristic analysis of disrupted rhythms with different sociodemographic backgrounds under the stress of COVID-19 pandemic, especially susceptible population suffering from rhythm disruption; 2), the correlations between rhythm disruption and psychological impacts, with explanations of disrupted rhythms on psychological impacts; 3), strategic measurement to restore the rhythm pattern; and 4), limitation; and 5), conclusion.

Responses to Additional Editor Comments:
1.Thank you for your interesting and timely research manuscript. The topic is clearly of current interest, however major revisions are necessary in order for this manuscript to be publishable. Reviewer 2 in particular provides comprehensive feedback on areas of the manuscript requiring significant revision.
Response: We have revised this manuscript according to the two reviewers' comments, with point-by-point responses.

2. Both reviewers recommend that the manuscript would benefit from thorough editing of language usage, spelling, and grammar by a fluent or native speaker of English. If you do not know anyone who can help you do this, you may wish to consider employing a professional scientific editing service.
Whilst you may use any professional scientific editing service of your choice, PLOS has partnered with both American Journal Experts (AJE) and Editage to provide discounted services to PLOS authors. Both organizations have experience helping authors meet PLOS guidelines and can provide language editing, translation, manuscript formatting, and figure formatting to ensure your manuscript meets our submission guidelines. To take advantage of our partnership with AJE, visit the AJE

	website (http://learn.aje.com/plos/) for a 15% discount off AJE services. To take advantage of our partnership with Editage, visit the Editage website (www.editage.com) and enter referral code PLOSEDIT for a 15% discount off Editage services. If the PLOS editorial team finds any language issues in text that either AJE or Editage has edited, the service provider will re-edit the text for free.<br>Response: Thank you for the advice. We have tried our best to improve the manuscript. In addition, we have sent our revised manuscript to Medjaden Inc, a professional editing and proofreading service, for language polishing.<br><br>3.In addition to the feedback provided by the reviewers I suggest that any revised submission particularly focus on improving the Discussion. A more sophisticated discussion around HPA axis activity, SCN regulation of circadian rhythms, and social rhythms is necessary (see also Reviewer 2's feedback on the Discussion). For example, the relevance of the "knockout monkey" model is unclear. I also suggest a more sophisticated discussion of the possible explanations for the observed gender differences in stress and anxiety. The manuscript relies on evidence from rat studies and, unusually, transgender individuals, to support increased HPA axis activity in females. A more nuanced discussion is needed that may or may not include reference to these biological differences between men and women, but that also considers differences in psychosocial contexts between the sexes and the impact of traditional gender roles and socio-cultural influences on higher rates of stress and anxiety among women in comparison to men. There is also an unusual reference to the impact of menopause on "women's circadian and endocrine disorders" that is not supported by a reference, nor is it a measured variable in the study.<br>Response: Thanks for your insightful and constructive advice. We have performed literature search, read relevant articles and revised Discussion section of the revised manuscript according to your professional suggestion and the advice of reviewer 2 (Page 19, Lines 11-20; Page 20, Lines 1-2; Page 21, Lines 6-21; Page 22, Lines 1-21; Page 23, Lines 1-21; Page 24, Lines 1-4 ; Page 25, Lines 5-8 and Lines 15-21; Page 26, lines 1-2.
Additional Information:	
Question	Response
**Financial Disclosure**<br><br>Enter a financial disclosure statement that describes the sources of funding for the work included in this submission. Review the submission guidelines for detailed requirements. View published research articles from *PLOS ONE* for specific examples.<br><br>This statement is required for submission and **will appear in the published article** if the submission is accepted. Please make sure it is accurate.	Include this sentence at the end of your statement: The funders had no role in study design, data collection and analysis, decision to publish, or preparation of the manuscript.

**Unfunded studies**<br>Enter: *The author(s) received no specific funding for this work.*<br><br>**Funded studies**<br>Enter a statement with the following details:<br>• Initials of the authors who received each award<br>• Grant numbers awarded to each author<br>• The full name of each funder<br>• URL of each funder website<br>• Did the sponsors or funders play any role in the study design, data collection and analysis, decision to publish, or preparation of the manuscript?<br>• **NO** - Include this sentence at the end of your statement: *The funders had no role in study design, data collection and analysis, decision to publish, or preparation of the manuscript.*<br>• **YES** - Specify the role(s) played.<br><br>* typeset	
**Competing Interests**<br><br>Use the instructions below to enter a competing interest statement for this submission. On behalf of all authors, disclose any competing interests that could be perceived to bias this work—acknowledging all financial support and any other relevant financial or non-financial competing interests.<br><br>This statement **will appear in the published article** if the submission is accepted. Please make sure it is accurate. View published research articles from *PLOS ONE* for specific examples.	The authors have declared that no competing interests exist.

Powered by Editorial Manager® and ProduXion Manager® from Aries Systems Corporation

**NO authors have competing interests**<br><br>Enter: *The authors have declared that no competing interests exist.*<br><br>**Authors with competing interests**<br><br>Enter competing interest details beginning with this statement:<br><br>*I have read the journal's policy and the authors of this manuscript have the following competing interests: [insert competing interests here]*<br><br>* typeset	
**Ethics Statement**<br><br>Enter an ethics statement for this submission. This statement is required if the study involved:<br><br>• Human participants<br>• Human specimens or tissue<br>• Vertebrate animals or cephalopods<br>• Vertebrate embryos or tissues<br>• Field research<br><br>Write "N/A" if the submission does not require an ethics statement.<br><br>General guidance is provided below. Consult the submission guidelines for detailed instructions. **Make sure that all information entered here is included in the Methods section of the manuscript.**	The study protocols was carried out in accordance with relevant guidelines and regulations and approved by the Ethics Committee of the Army Medical Center(Approval No. 2019-144). Before filling out the questionnaire, you were asked to read and check the informed consent and then continue to complete the questionnaire; those under 18 years old were asked to consent by their guardians and themselves. All completed questionnaires were received and checked for validity and completeness.

Powered by Editorial Manager® and ProduXion Manager® from Aries Systems Corporation

**Format for specific study types**<br><br>**Human Subject Research (involving human participants and/or tissue)**<br>• Give the name of the institutional review board or ethics committee that approved the study<br>• Include the approval number and/or a statement indicating approval of this research<br>• Indicate the form of consent obtained (written/oral) or the reason that consent was not obtained (e.g. the data were analyzed anonymously)<br><br>**Animal Research (involving vertebrate animals, embryos or tissues)**<br>• Provide the name of the Institutional Animal Care and Use Committee (IACUC) or other relevant ethics board that reviewed the study protocol, and indicate whether they approved this research or granted a formal waiver of ethical approval<br>• Include an approval number if one was obtained<br>• If the study involved *non-human primates*, add *additional details* about animal welfare and steps taken to ameliorate suffering<br>• If anesthesia, euthanasia, or any kind of animal sacrifice is part of the study, include briefly which substances and/or methods were applied<br><br>**Field Research**<br><br>Include the following details if this study involves the collection of plant, animal, or other materials from a natural setting:<br>• Field permit number<br>• Name of the institution or relevant body that granted permission	
**Data Availability**<br><br>Authors are required to make all data underlying the findings described fully available, without restriction, and from the time of publication. PLOS allows rare exceptions to address legal and ethical concerns. See the PLOS Data Policy and FAQ for detailed information.	Yes - all data are fully available without restriction

A Data Availability Statement describing where the data can be found is required at submission. Your answers to this question constitute the Data Availability Statement and **will be published in the article**, if accepted.<br><br>**Important:** Stating 'data available on request from the author' is not sufficient. If your data are only available upon request, select 'No' for the first question and explain your exceptional situation in the text box.<br><br>Do the authors confirm that all data underlying the findings described in their manuscript are fully available without restriction?	
**Describe where the data may be found in full sentences. If you are copying our sample text, replace any instances of XXX with the appropriate details.**<br><br>• If the data are **held or will be held in a public repository**, include URLs, accession numbers or DOIs. If this information will only be available after acceptance, indicate this by ticking the box below. For example: *All XXX files are available from the XXX database (accession number(s) XXX, XXX.).*<br>• If the data are all contained **within the manuscript and/or Supporting Information files**, enter the following: *All relevant data are within the manuscript and its Supporting Information files.*<br>• If neither of these applies but you are able to provide **details of access elsewhere**, with or without limitations, please do so. For example:<br><br>*Data cannot be shared publicly because of [XXX]. Data are available from the XXX Institutional Data Access / Ethics Committee (contact via XXX) for researchers who meet the criteria for access to confidential data.*<br><br>*The data underlying the results presented in the study are available from (include the name of the third party*	All relevant data are within the manuscript and its Supporting Information files.

and contact information or URL).<br>• This text is appropriate if the data are owned by a third party and authors do not have permission to share the data.<br><br>* typeset	
Additional data availability information:	

Powered by Editorial Manager® and ProduXion Manager® from Aries Systems Corporation

Dear Ms. Kiss,

Thank you for further advice on our manuscript (ID: PONE-D-20-26146R1), entitled "**Disrupted rhythms of life, work and entertainment and their associations with psychological impacts under the stress of the COVID-19 pandemic: A Survey in 5854 Chinese people with different sociodemographic backgrounds**". According to your advice, we have revised our manuscript. All changes are highlighted in red in the newly revised manuscript. In addition, we have responded to your advice in a point-by-point format, which are listed below this letter.

We hope that the revision is acceptable, and we look forward to hearing from you soon.

Kind regards,

Dr Dongfeng Chen

Response to your advice

1. In your Methods section, please include additional information about your dataset and ensure that you have included a statement specifying whether the collection method complied with the terms and conditions for the website."

Reply: We have included a statement specifying that the collection method complied with the terms and conditions for the website (Page 6, Lines 21; Page 7, Line 1).

2. Thank you for stating the following at the beginning of your manuscript:

"This study was supported by the grant from Chongqing basic research and foreword exploration project, China (Grant No.CSTC2018jcyjAX0620); and Chongqing Medical University, a special project of the emergency clinical research on the new coronavirus disease (general project): (1) Psychological intervention for first-line medical personnel in the new coronavirus pneumonia pandemic, and (2) psychological intervention model of negative emotion and behavior among hospital workers during the COVID-19 pandemic."

* We note that you have provided funding information that is not currently declared in your Funding Statement.

**However, funding information should not appear in the beginning or other areas of your manuscript. We will only publish funding information present in the Funding Statement section of the online submission form, which only we can update on your behalf.

** Please remove any funding-related text from the manuscript and let us know how you would like to update your Funding Statement.

Currently, your Funding Statement reads as follows:

'Include this sentence at the end of your statement: The funders had no role in study design, data collection and analysis, decision to publish, or preparation of the manuscript.'

Reply: We have removed this statement from the manuscript, and added it in the Funding Statement section of the online submission form according to your suggestion, which reads: "This study was supported by the grant from Chongqing basic research and foreword exploration project, China (Grant No.CSTC2018jcyjAX0620); and Chongqing Medical University, a special project of the emergency clinical research on the new coronavirus disease (general project): (1) Psychological intervention for first-line medical personnel in the new coronavirus

pneumonia pandemic, and (2) psychological intervention model of negative emotion and behavior among hospital workers during the COVID-19 pandemic."

3. *** Please include your amended statements within your COVER LETTER; we will change the online submission form on your behalf. ***

Reply: The amended statement is described above.

Manuscript

Click here to access/download;Manuscript;Manuscript, 1-25.docx

Disrupted rhythms of life, work and entertainment and their associations with psychological impacts under the stress of the COVID-19 pandemic: A Survey in 5854 Chinese people with different sociodemographic backgrounds

Min Yang*[1], Ping He*[2], Xiaoming Xu*[3], Dan Li*[4], Jing Wang[1], Yanjun Wang[1], Bin Wang[1], Wo Wang[5], Mei Zhao[4], Hui Lin[6], Mingming Deng[#7], Tianwei Deng[#8], Li Kuang[#3], Dongfeng Chen [#1]

[1]Army Medical Center of PLA, Daping Hospital, Army Medical University, 10 Changjiang Branch Road, Daping, Yuzhong District, Chongqing 400042, P.R. China;

[2]Department of Gastroenterology, Yongchuan Hospital of Chongqing Medical University, 439 Xuanhua Road, Yongchuan District, Chongqing 402160, P.R. China;

[3]Department of Psychiatry, the First Affiliated Hospital of Chongqing Medical University, 1 Youyi Road, Yuzhong District, Chongqing 400016, P.R. China;

[4] CAS Key Laboratory of Mental Health, Institute of Psychology, 16 Lincui Road, Chao Yang District, Beijing 100101, P.R. China;

[5] Mental Health Center, University-Town Hospital of Chongqing Medical University, 55 Middle Road, Shapingba District, Chongqing 401331, P.R. China;

[6] Department of Statistics, Army Medical University, 30 Gaotanyan Street, Shapingba District Chongqing 400038, P.R. China;

[7] Department of Gastroenterology, The Affiliated Hospital of Southwest Medical University, 25 Taiping Street, Jiangyang District, Luzhou 64600, P.R. China

[8] Department of Gastroenterology, Three Gorges Hospital of Chongqing University, 165 Xincheng Road, Wanzhou, Chongqing 404000, P.R. China;

[#]Corresponding authors: Dongfeng Chen, Li Kuang, Tianwei Deng, Mingming Deng.

Email:
chendf1981@126.com(CDF);kuangli0308@163.com(LK);dengtw1986@163.com(DTW);793070544@qq.com(DMM).

* These authors contributed equally to this work.

# These authors also contributed equally to this work.

Data Availability Statement: All relevant data are within the paper and its Supporting Information files.

Competing Interests: The authors have declared that no competing interests exist.

Abstract

Background & Aim The coronavirus disease 2019 (COVID-19) pandemic has affected the life and work of people worldwide. The present study aimed to evaluate the rhythm disruptions of life, work, and entertainment, and their associations with the psychological impacts during the initial phase of the COVID-19 pandemic.

Method A cross-sectional study was conducted from the 10th to 17th March 2020 in China. A structured e-questionnaire containing general information, the Chinese version of Brief Social Rhythm Scale, and Zung's self-rating scales of depression and anxiety (SDS and SAS) was posted and collected online through a public media (*i.e.* EQxiu online questionnaire platform). Scores in sleeping, getting up, and socializing (SGS) rhythm and eating, physical practice, and entertainment (EPE) rhythm were compared among and between participants with different sociodemographic backgrounds including gender, age, education, current occupation, annual income, health status, and chronic disease status. Correlations of SDS and SAS with SGS-scale and EPE-scale were analyzed.

Results Overall, 5854 participants were included. There were significant differences in the scores of SGS-scale and EPE-scale among people with different sociodemographic backgrounds. The scores were significantly higher in the groups

with female gender, low education level, too-low or too-high income, poor health status, ages of 26-30 years or older than 61 years, nurses and subjects with divorce or widow status. There were also significant differences in SAS and SDS scores among people with different sociodemographic backgrounds (all $P < 0.05$). The overall prevalence of depression and anxiety was 24.3% and 12.6%, respectively, with nurses having the highest rates of depression (32.94%) and anxiety (18.98%) among the different occupational groups. SGS-scale was moderately correlated with SDS and SAS, and disruption of SGS rhythm was an independent risk factor for depression and anxiety.

Conclusion Social rhythm disruption was independently associated with depression and anxiety. Interventions should be applied to people vulnerable to the rhythm disruption during the COVID-19 pandemic.

Key words: Coronavirus disease 2019 (COVID-19); Social rhythm disruption; Anxiety and depression; Psychological impacts; Chinese people.

Introduction

Coronavirus disease 2019 (COVID-19) is an acute respiratory infectious disease caused by a novel coronavirus, called severe acute respiratory syndrome coronavirus 2 (SARS-CoV-2)[1-3]. Due to the strong infectivity of the coronavirus and the lack of effective treatments and vaccines, COVID-19 has spread rapidly and become a pandemic worldwide with serious consequences. The social restriction policies implemented to control the infection in almost all countries have resulted in unemployment, financial hardship, and a reduction in quality of life in many countries (Bashir, M. F. 2020)[4], which have seriously affected the life and work of people. Simultaneously, the COVID-19 pandemic has alarming implications for a global increase in mental health problems, and the psychological burden during the COVID-19 pandemic has been reported[5, 6].

Social rhythm is defined as the regularity of timing of the behaviors in social activities and also described clinically as “daily routines”[7]. COVID-19-related social changes, particularly lockdown protocols, have indeed impacted the timing of daily behaviors in stabilizing circadian function, and social rhythm disruption may be correlated with psychological impacts[7]. Previous studies have reported that social rhythm disruption is connected with mental diseases, especially depression and bipolar disorder in the non-pandemic days, which has been explained by many theories[8-11]. Recently, several studies have reported social and circadian rhythm disruptions in students, office workers, the elder, and patients with narcolepsy or autism[12-14].

However, most of these studies mainly concentrated on sleep disruption, and none of them investigated the disrupted rhythms of life, work and entertainment behaviors and their associations with psychological impacts under the stress of the COVID-19 pandemic. We hypothesize that people with different sociodemographic backgrounds have different degrees of rhythm disruption in life, work, and entertainment, that the rhythm disruption is more severe in some specific groups, and that the rhythm disruption is associated with psychological impacts during the initial phase of the COVID-19 pandemic. Therefore, the aim of the present study was to evaluate the rhythm disruptions in life, work, and entertainment and their associations with the psychological impacts in the general Chinese population with different sociodemographic backgrounds during the initial phase of the COVID-19 pandemic.

Materials and Methods

Participants and procedure

Chinese people with different sociodemographic backgrounds voluntarily participated in this online survey. An e-questionnaire was designed to investigate the rhythm activities and psychological impacts in Chinese people with different backgrounds including gender, age, educational background, annual income, health status, current occupation, and chronic disease status during the COVID-19 pandemic through EQxiu online questionnaire platform. The collection method was complied with the terms and

conditions for the website. The survey was conducted from 10-17 March 2020 in 22 provinces and five autonomous regions, four municipalities, and two special administrative regions (*i.e.*, Hong Kong and Macao) of China. The study protocol was carried out following relevant guidelines and regulations and approved by the Ethics Committee of the Army Medical Center (Approval No. 2019-144). During the survey, the participants were asked to read and check the informed consent and then complete the questionnaire; those under 18 years old were asked to consent by their guardians before completing the questionnaire. All completed questionnaires were received and checked for validity and completeness.

Measures

Social rhythm. The Brief Social Rhythm Scale (BSRS) was developed to quickly assess social rhythmicity in large-scale multi-national samples and multiple languages[15]. The Chinese version of BSRS also included entertainment and physical practice, and was proved to have good reliability and validity in China[16]. The scale consisting of 17 items was used to investigate an individual's rhythm in six aspects, i.*e.*, sleeping (going to bed), getting up, socializing (SGS), eating, physical practice, and entertainment (EPE) on weekdays and weekends. The first eight items addressing SGS rhythm constituted SGS-scale, and the last nine items addressing EPE rhythm constituted EPE-scale. Each item was assigned with a score of 1, 2, 3, 4, 5, or 6,

corresponding to categories of very regularly, quite regularly, somewhat regularly, somewhat irregularly, quite irregularly, or very irregularly, respectively. The lower the score was, the more regular the rhythm was.

Zung’s depression and anxiety scale. Zung's self-rating depression scale (SDS) and Zung's self-rating anxiety scale (SAS) were used to evaluate the psychological impacts of each participant[17, 18]. There were 20 items in SAS and SDS, and each item was graded to 1-4 reflecting never, often, sometimes, and always. The rough score was the sum of 20 questions multiplied by 1.25 and rounded to get the standard score. The cut-off value was 50 for both SAS and SDS. The higher the score, the more obvious the anxiety or depression was, with the scores of 50-59, 60-69, and 70 points or more indicating mild, moderate, and severe anxiety or depressive symptom, respectively. The Chinese version of SDS and SAS, which was used in the present study, was reported to present good reliability, validity and excellent internal consistency, with the Cronbach coefficient of 0.784 for SDS in Chinese people[19], and of 0.931 for SAS in the general population[20].

Statistical analysis

Data were analyzed using SPSS version 23.0 (SPSS, Chicago, IL, USA). The data of normal distribution was expressed by mean±standard deviation. One-way analysis of variance with a post-hoc analysis, was used to make comparisons among groups. T-test

or a nonparametric test (*i.e.*, Mann-Whitney U test or Kruskal-Wallis test), where appropriate, was used to make comparisons between the group with the highest score and each of the other groups for each item. Pearson correlation or Spearman correlation, where appropriate, was used to determine the correlation between the scales of social rhythm scale and the Zung's scales. In addition, a multivariate linear regression analysis was used to determine the independent factors for the correlation of anxiety or depression with social rhythm. A P value of < 0.05 was considered statistically significant.

Results

Sociodemographic characteristics of the participants

A total of 5872 people checked the e-questionnaire. Eighteen questionnaires with invalid were excluded the data analysis due to frivolous or incomplete answers, or the same option for different questions or orderly answers. The data of 5854 participants with valid questionnaires were further analyzed. The general information on the sociodemographic characteristics of the participants is listed in Table 1.

Table 1. General sociodemographic characteristics of 5854 analyzed participants

Feature		N (%)	Feature		N (%)
Gender	Female	3439(58.75%)	Health status	Poor	53(0.91%)
	Male	2415(41.25%)		Normal	787(13.44%)

Age	-18	68(1.16%)		Good	3686(62.97%)
	18-25	1960(33.48%)		Very good	1328(22.69%)
	26-30	862(14.72%)	Current occupation	Businessman	481(8.22%)
	31-40	1339(22.87%)		Officer	245(4.19%)
	41-50	852(14.55%)		Teacher	231(3.95%)
	51-60	682(11.65%)		Police	277(4.73%)
	61-	91(1.55%)		Farmer	217(3.71%)
Marital status	Unmarried	2435(41.60%)		Employee	270(4.61%)
	Married	3232(55.21%)		Doctor	1171(20.00%)
	Divorced /widowed	187(3.19%)		Nurse	1017(17.37%)
Chronic disease	No chronic disease	4373(74.70%)		Medical technician	139(2.37%)
	CDCPD	671(11.46%)		Retired re-employee	229(3.91%)
	Chronic diseases only	810(13.84%)		Non-medical student	1172(20.02%)
Annual income	Less than 50000 CY	2482(42.40%)		Medical student	405(6.92%)
	50000-100000 CY	1849(31.59%)	Education	Junior high school or lower	97(1.66%)
	100000-200000 CY	1188(20.29%)		Senior high school	535(9.14%)
	200000-300000 CY	225(3.84%)		Junior college or undergraduate	4465(76.27%)
	More than 300000 CY	110(1.88%)		Master or above	757(12.93%)

CY, Chinese yuan; CDCPD, chronic diseases comorbid with psychosomatic diseases.

Disruptions of social rhythm in the participants

Generally, there were significant differences in SGS-scale and EPE-scale among participants with different sociodemographic backgrounds including gender, age,

educational background, annual income, health status, current occupation, and chronic disease status (all $P<0.05$, Table 2). Moreover, the SGS-scale and EPE-scale scores were compared between the group with the highest score and each of other groups (Table 3). Specifically, the female gender and poor health status were most closely associated with disrupted rhythms of life, work, and entertainment. The age group of 26-30 years, nurses and subjects with divorce or widow status, the education level of senior high school, annual pre-tax income of 50,000-100,000 Chinese Yuan (CY), or chronic disease comorbid with psychosomatic diseases mostly suffered from disrupted rhythms of life and work. The age group of older than 61, and subjects with education levels of master's degree or above, annual pre-tax income over 300,000 CY, or chronic disease without psychosomatic diseases mostly suffered from disrupted rhythms of entertainment. Especially, nurses showed significantly more severe disruption of SGS rhythm, whereas the elderly reported significant irregularity in EPE rhythm (Table 2).

Table 2. Scores of social rhythms in participants with different sociodemographic backgrounds during the COVID-19 pandemic (N=5854).

Feature		SGS-scale			EPE-scale		
		Mean±SD	Z/X2	*P*	Mean±SD	Z/X2	*P* value
Gender	Female	21.64±9.03	-10.183	<0.001	7.71±6.02	-7.627	<0.001
	Male	19.24±8.80			8.71±5.93		
Age	-18	17.63±8.69	146.237	<0.001	7.82±5.58	112.103	<0.001
	18-25	18.98±8.95			7.75±5.35		

	26-30	23.04±9.77			7.22±6.22		
	31-40	21.54±8.95			7.76±6.18		
	41-50	20.79±8.56			9.10±6.68		
	51-60	20.74±7.91			9.64±5.82		
	61-	21.27±8.78			9.87±6.14		
Marital status	Unmarried	19.53±9.19	81.065	<0.001	7.81±5.52	2.499	0.294
	Married	21.42±8.77			8.32±6.26		
	Divorced /widowed	22.04±9.21			8.76±7.25		
Education	Junior high school or lower	18.92±10.07	8.880	0.031	7.81±7.18	11.172	0.011
	Senior high school	21.18±8.97			7.75±5.97		
	Junior college or undergraduate	20.64±9.06			8.05±5.89		
	Master or above	20.59±8.61			8.85±6.46		
Annual income	Less than 50000 CY	19.21±8.90	126.387	<0.001	7.88±5.64	48.455	<0.001
	50000-100000 CY	22.18±9.26			7.68±6.06		
	100000-200000 CY	21.28±8.49			8.93±6.28		
	200000-300000 CY	20.66±8.41			9.31±6.03		
	More than 300000 CY	20.78±9.03			9.86±8.12		
Health status	Poor	28.02±10.00	500.564	<0.001	9.53±8.85	26.969	<0.001
	Normal	25.21±9.48			7.94±7.19		
	Good	20.93±8.36			8.35±5.90		
	Very good	16.90±8.85			7.53±5.30		
Current occupation	Businessman	20.21±8.50	322.663	<0.001	8.39±5.92	177.273	<0.001
	Officer	19.89±8.18			9.32±5.94		
	Teacher	21.06±8.41			9.74±6.77		

	Police	18.69±8.79			9.05±5.46		
	Farmer	19.81±9.55			7.47±6.17		
	Employee	20.49±7.99			8.44±5.81		
	Doctor	20.99±8.27			8.52±6.39		
	Nurse	24.47±9.91			6.46±6.08		
	Medical technician	21.31±8.65			7.63±6.56		
	Retired re-employee	21.63±8.78			9.62±5.28		
	Non-medical student	17.65±8.46			7.90±5.20		
	Medical student	20.64±8.80			8.66±6.08		
Chronic disease	No chronic disease	19.71±8.82	202.421	<0.001	7.94±5.67	6.269	0.044
	CDCPD	24.01±9.18			8.67±7.40		
	Chronic diseases only	22.98±8.82			8.68±6.37		

SD, standard deviation; CY, Chinese yuan; SGS-scale: the rhythm of sleep, getting-up, and socializing; EPE-scale: the rhythm of eating, physical practice, and entertainment activities; CDCPD, Chronic diseases comorbid with psychosomatic diseases.

Table 3. Comparison of SGS-scale and EPE-scale scores between the group with the highest score and each of the other groups in terms of sociodemographic backgrounds

Features	SGS-scale			EPE-scale		
	Highest-score group	Other groups	*P*-value	Highest-score group	Other groups	*P*-value
Marital status	Divorced /widowed	Unmarried	0.001	Divorced /widowed	Unmarried	0.227
		Married	0.748		Married	0.802

Chronic disease	CDCPD	No chronic disease	<0.001	Chronic diseases only	No chronic disease	0.006
		Chronic diseases only	0.086		CDCPD	1.000
Education	Senior high school	Junior high school or lower	0.222	Master or above	Junior high school or lower	0.695
		Junior college or undergraduate	0.720		Senior high school	0.010
		Master or above	0.811		Junior college or undergraduate	0.009
Health status	Poor	Normal	0.275	Poor	Normal	0.749
		Good	<0.001		Good	0.918
		Very good	<0.001		Very good	0.499
Annual income	50000-100000 CY	Less than 50000 CY	<0.001	More than 300000 CY	Less than 50000 CY	0.119
		100000-200000 CY	0.056		50000-100000 CY	0.063
		200000-300000 CY	0.113		100000-200000 CY	0.938
		More than 300000 CY	0.714		200000-300000 CY	0.999
Age	26-30	-18	<0.001	61-	-18	0.473
		18-25	<0.001		18-25	0.034
		31-40	0.006		26-30	0.003
		41-50	<0.001		31-40	0.042
		51-60	<0.001		41-50	0.998
		61-	0.799		51-60	1.000
Current occupation	Nurse	Businessman	<0.001	Teacher	Businessman	0.482
		Officer	<0.001		Officer	1.000
		Teacher	<0.001		Police	1.000
		Police	<0.001		Farmer	0.015
		Farmer	<0.001		Employee	0.781

	Employee	<0.001	Doctor	0.551
	Doctor	<0.001	Nurse	<0.001
	Medical technician	0.007	Medical technician	0.199
	Retired re-employee	0.001	Retired re-employee	1.000
	Non-medical student	<0.001	Non-medical student	0.008
	Medical student	<0.001	Medical student	0.953

CY, Chinese yuan; SGS-scale: the rhythm of sleep, getting-up, and socializing; EPE-scale: the rhythm of eating, physical practice, and entertainment activities; CDCPD, Chronic diseases comorbid with psychosomatic diseases.

Psychological impacts in the participants

There were significant differences in SAS and SDS scores among participants with different genders, ages, marital status, education levels, annual pre-tax incomes, health status, current occupation, and chronic diseases with or without psychosomatic diseases in each of the variables (all $P<0.05$) (Table 4). Specifically, nurses and participants with female gender, divorce or widow status, education levels of junior high school or below, poor health status, and chronic diseases comorbid with psychosomatic diseases mostly suffered from depression and anxiety. Whereas the age group of 26-30 years, and participants with annual pre-tax income of 50,000-100,000 CY mostly suffered from depression, the age group of 31-40 years and participants with annual pre-tax income over 300,000 CY mostly suffered from anxiety. Additionally, SDS scores were

significantly higher in medical students than in non-medical students (*P*<0.001). There were significant differences in the prevalence of depression and anxiety among participants with different sociodemographic backgrounds (all *P*<0.05, S1 and S2 Tables). The overall prevalence of depression and anxiety was 24.33% and 12.64%, respectively, as defined by SDS or SAS scores over 50. Among the different occupational groups, nurses had the highest rates of depression (32.94%) and anxiety (18.98%). Participants with chronic diseases combined with psychosomatic diseases had the highest rates of depression (45.90%) and anxiety (31.45%).

Table 4. Scores of depression and anxiety in participants with different sociodemographic backgrounds during the COVID-19 pandemic (N=5854).

Feature		SDS			SAS		
		Mean±SD	Z/X2	*P*	Mean±SD	Z/X2	*P*
Gender	Female	43.34±11.66		<0.001	38.8±9.39		<0.001
	Male	39.97±11.59			37.19±9.23		
Age	-18	39.34±10.93		<0.001	35.09±8.35		<0.001
	18-25	40.13±11.63			35.82±8.27		
	26-30	43.87±12.20			39.66±9.97		
	31-40	43.59±11.68			39.84±9.76		
	41-50	42.48±11.89			39.4±9.94		
	51-60	41.27±10.76			38.12±8.61		
	61-	41.03±10.97			38.91±9.32		
Marital	Unmarried	40.65±11.79		<0.001	36.26±8.56		<0.001

status	Married	42.78±11.60		39.39±9.61	
	Divorced /widowed	44.57±12.05		40.87±10.63	
Education	Junior high school or lower	45.22±13.29	<0.05	39.85±10.43	<0.001
	Senior high school	42.83±11.37		38.57±9.38	
	Junior college or undergraduate	41.71±11.67		37.92±9.24	
	Master or above	42.31±12.17		38.86±9.85	
Annual income	Less than 50000 CY	40.73±11.74	<0.001	36.49±8.86	<0.001
	50000-100000 CY	43.54±11.65		39.72±9.45	
	100000-200000 CY	42.46±11.79		39.17±9.67	
	200000-300000 CY	39.56±10.12		36.93±8.02	
	More than 300000 CY	42.19±12.56		40.05±11.42	
Health status	Poor	62.90±11.77	<0.001	55.94±12.3	<0.001
	Normal	49.73±12.34		44.56±10.55	
	Good	42.00±10.57		37.95±8.48	
	Very good	36.36±10.88		34.14±7.86	
Current occupation	Businessman	40.42±10.59	<0.001	37.29±8.37	<0.001
	Officer	40.25±9.25		37.86±7.63	
	Teacher	41.58±11.24		38.58±9.38	
	Police	38.89±11.34		36.71±8.89	
	Farmer	43.02±11.59		37.97±9.14	
	Employee	42.08±10.68		38.25±8.34	
	Doctor	43.85±12.39		40.12±10.32	
	Nurse	45.17±12.35		40.41±10.14	
	Medical technician	44.74±11.84		40.14±9.77	

	Retired re-employee	42.24±10.47		38.98±8.61	
	Non-medical student	38.43±10.73		34.5±7.32	
	Medical student	41.93±12.18		37.93±9.59	
Chronic disease	No chronic disease	40.27±11.12	<0.001	36.48±8.4	<0.05
	CDCPD	48.86±12.70		44.88±10.97	
	Chronic diseases only	45.27±11.42		41.47±9.45	

SD, standard deviation; CY, Chinese yuan; SGS-scale: the rhythm of sleep, getting-up, and socializing; EPE-scale: the rhythm of eating, physical practice, and entertainment activities; CDCPD, Chronic diseases comorbid with psychosomatic diseases.

Correlations between the social rhythm and psychological impacts

Among the 5854 participants, SGS-scale and EPE-scale were 20.65±9.01 and 8.12±6.00, respectively, and SAS score and SDS score were 30.51±7.49 and 41.95±11.75, respectively. Spearman correlation analysis found that SGS scale was moderately correlated with depression and anxiety, with the correlation coefficients of 0.550 and 0.544, respectively, (both P<0.001, S3 Table). In the multivariate linear regression analysis where the scores of SPS-scale or EPE-scale were taken as dependent variables, the score of SDS and SAS were taken as independent variables, and the age, gender, education, and occupation status were taken as confounding factors, the correlations between the scores of SPS-scale and EPE-scale and the scores of SDS and SAS remained unchanged (all P<0.05, Table 5, S1 Fig) .The disruption of sleep,

getting-up, and socializing rhythm was positively associated with depression and anxiety (*β*=0.355, *P*<0.001; β=0.186, *P*<0.001). The disruption of eating, physical practice, and entertainment rhythm showed opposite association to psychological impact: negatively related to depression and positively related to anxiety (*β*= -0.049, *P*=0.036; *β*= 0.082, *P*<0.001) (Table 5).

Table 5. Correlations between the social rhythm disruption and psychological impacts as determined by multivariate linear regressive analysis

			**Standardized coefficient (β)**	***t***	***P***	**EXP(B) 95% CI**	
						**Lower**	**Upper**
SGS-scale	Model 1	SDS	0.381	20.080	<0.001	0.264	0.321
		SAS	0.203	10.689	<0.001	0.160	0.231
	Model 2	SDS	0.373	19.463	<0.001	0.258	0.315
		SAS	0.200	10.385	<0.001	0.156	0.229
	Model 3	SDS	0.355	18.370	<0.001	0.243	0.302
		SAS	0.188	9.791	<0.001	0.145	0.217
	Model 4	SDS	0.355	18.373	<0.001	0.244	0.302
		SAS	0.186	9.645	<0.001	0.143	0.216
EPE-scale	Model 1	SDS	-0.082	-3.565	<0.001	-0.065	-0.019
		SAS	0.109	4.780	<0.001	0.041	0.099
	Model 2	SDS	-0.049	-2.129	0.033	-0.048	-0.002
		SAS	0.082	3.582	<0.001	0.024	0.082
	Model 3	SDS	-0.049	-2.101	0.036	-0.048	-0.002

	SAS	0.081	3.513	<0.001	0.023	0.081
Model 4	SDS	-0.049	-2.101	0.036	-0.048	-0.002
	SAS	0.082	3.518	<0.001	0.023	0.081

SGS-scale: the rhythm of sleep, getting-up, and socializing; EPE-scale: the rhythm of eating, physical practice, and entertainment activities; SDS/ SAS: Zung's self-rating depression and anxiety scales.

Model 1: uncorrected; Model 2: corrective factors: gender, age, marital status, and education level; Model 3: corrective factors: based on the corrective factors of Model 2 and health status, annual pre-tax income, and current occupation; and Model 4: corrective factors: based on the corrective factors of Model 3 and psychosomatic diseases. The results of Model 4 were presented in this table.

Discussion

The COVID-19 pandemic has brought great challenges and altered daily routines. It also undoubtedly contributes to emotional stress, fear, sadness, loneliness, anxiety and depression, and thus is associated with a global increase in psychological impacts[5]. One of the many mechanisms by which COVID-19-related changes in social rhythm impact mental health involves circadian disruption[7]. A previous study showed that the early identification and intervention of rhythm disruption are of important clinical

significance[21]. Rhythm disruption is part of the pathogenic cascade and is associated with mood disorders, such as anxiety, depression, low happiness, strong loneliness, and bipolar disorder during the non-outbreak period of infectious disease[7, 15, 21]. Limited studies have found that sleep disruption is related to depression in the general population during the COVID-19 pandemic[11]. The present study found that the disruption of social rhythm was quite different among people with various sociodemographic backgrounds. The female gender, low-degree education level, too-low or too-high income, poor health status, age group of 26-30 years and older than 61 years, nurses and subjects with divorce mostly suffered from disruption of social rhythm. In addition, the present study observed that the participants with female gender, age group of 26-40 years, divorce or widow status, low education level, too-low or too-high income, poor health status, and chronic diseases comorbid with psychosomatic diseases, and nurses mostly suffered from depression and/or anxiety.

There are two major possible explanations for the observed gender differences in rhythm disruption, depression, and anxiety. First, the steroid receptors are expressed in almost every site that receives direct suprachiasmatic nucleus (SCN) input[22]. The endogenous circadian clocks can be reset by hormone signals, affecting the balance of the hypothalamus-pituitary-adrenal (HPA) axis, and making the HPA axis more responsive to stress than males[22, 23]. The disruption of circadian rhythms within the HPA axis, and the sleep-arousal system differs between the genders, and is associated with dysfunction and disease[22]. Understanding gender differences in the circadian

timing system can lead to improved treatment strategies. Second, the psychosocial contexts differ between the genders, and the impact of traditional gender roles and socio-cultural influences on stress and anxiety are greater in women than in men[24].

In this study, we found that people with low education levels or low income mostly suffer from irregular social rhythms, likely due to their weak competitiveness in the job market and inability to adapt to the new E-commerce models during the COVID-19 pandemic due to the isolation or even lockdown. Moreover, they did not have sufficient judgment while facing various, overloaded and timeless information on the COVID-19 pandemic, and easily felt stressed, depressive and anxious[25]. Interestingly, in the present study, the people with high income were among those who mostly suffered from disrupted rhythms of entertainment during the COVID-19 pandemic. It has been shown that people with poor health status are likely to have basic diseases, weak immune function, and low ability to fight pathogens or adapt to a rhythm change[26], which may explain that people with poor health status suffered from disruption of social rhythm observed in the represent study. In addition, our finding that a high frequency of the irregular rhythm in the divorced group might be related to the disintegration of the family, loose family relations, economic problems, and the problem of raising children as a single parent. Especially, the isolation during the COVID-19 pandemic might have aggravated the fear for the unknown future, loneliness, helplessness, or irregular lifestyle[27].

The present study found that the people aged 26-40 years old and the elderly reported similarly high SGS-scale scores and were vulnerable to circadian disruption. The former is the main force of the society that promote the consumption, cultural creativity, and economic development[28]. However, when they must stay at home or fight against the COVID-19 pandemic due to the sudden outbreak of the disease, it is difficult for them to maintain a normal social rhythm. It is worth noting that the elderly reported the highest rate of rhythm disruption of eating, physical practice, and entertainment behaviors in the present study. Previously, Liu *et al.* [29]conducted a study on lifestyle and the health status of Chinee elderly, which included the elderly who lived in their own houses, with 20.6% of them being widows or widowers. They found that lack of physical practice, advanced age, and alcohol consumption were risk factors for their self-rate health status whereas a long sleep time was the protective factor[29]. Indeed, it has been reported that advanced age is characterized by a progressive decreasing amplitude and phase advance of circadian rhythmicity in overall biological functions, including blood pressure, hormonal circadian secretions, and immune function changes[30].

It is noteworthy to mention that nurses had the highest score of rhythm disruption in going to bed, get-up, and socializing among occupations, suggesting that they were more affected by the pandemic than other occupations such as businessmen, officers, teachers, police, farmers, doctors, and students. Night shift work has been thought to play a role through a disruption of the circadian rhythm, decreased synthesis of

melatonin and sleep deprivation[31]. We assume that nurses undertake a working articulation in shifts to keep the continuity of healthcare throughout a whole day, and the COVID-19 pandemic results in heavy workload, insufficient rest, quarantine, and social isolation. Consequently, the already dysfunctional social rhythm of nurses is further exacerbated. McElroy *et al*. reported that night and long shifts caused fatigue and adversely affected the family and social life of hospital employees[32]. Rosa *et al.* indicated that shift work could cause psychological consequences, including anxiety, stress, and depression, which is an obstacle for social and family relationships and a risk factor for metabolic disorders, diabetes, cardiovascular disorders, and breast cancer[33].

The present study firstly reported the critical connection between social rhythm disruption and psychological impacts in a large-scale population under the stress of such a pandemic. Our study further found that the scores of social rhythms were positively correlated with SDS and SAS scores under the stress of the COVID-19 pandemic. The following factors are speculated to be involved in the connection. The first factor is the social zeitgeber theory that offers an explanation. The notion that life events increase the likelihood of mood episodes *via* decreased zeitgeber scaffolding for a vulnerable circadian system has been known as the social zeitgeber hypothesis, and the "social rhythm" refers to the cross-day stability of the timing of behaviors that act as zeitgebers[7]. Social stimuli under the stress of COVID-19 may affect the circadian behavioral programs by regulating the phase and period of circadian clocks (*i.e.* a

zeitgeber action, either direct or by conditioning to photic zeitgebers) which leads to various adverse mental health[34, 35]. In turn, depressive episodes that arise because of life events disturbing social zeitgebers subsequently affect biological rhythms, and thus derail social and biological rhythms [35-36]. This present study expanded the scope of application of these rhythm theories from patients with mood disorders to the population with different sociodemographic backgrounds. The second factor is the lockdown protocols that have been adopted to minimize COVID-19 pandemic transmission. The protocols may weaken the zeitgeber-setting mechanism, and impact the stability of the timing of daily behaviors, such as sleeping, getting up, socializing, eating, physical practice, and entertainment, which, in turn, lead to mental health disorders, such as anxiety and depression[7]. The third factor is SCN that serves as a master clock synchronizing the phase of clocks throughout the body. The disrupted rhythm in SCN leads to helplessness and behavioral despair, which increases depression and anxiety-related behaviors[36]. The fourth factor is the daily function of HPA activity, which is also regulated by the biological clock[37]. Anxiety disorders and major depressive disorder have been associated with increased and blunted HPA axis reactivity to social stress, which engages the diverse biological pathways that bolster successful stress adaptation and promote stress resilience. Adaptation to the challenges of stress is compromised when these pathways are no longer functioning optimally, resulting in the disrupted rhythms on psychological impacts[38]. Of course, other mechanisms may also connect social rhythm dysregulation with phycological impacts.

A previous study reported that the 4/4 genotype of the circadian gene, *PER3*, was associated with anxiety symptoms in 804 Brazilian non-clinical young adults[39]. Mathew and his colleagues also found that social jetlag (a misalignment between sleep timing on the weekend and school week) was positively and independently associated with anxiety symptoms in adolescents[40].

Based on the findings of the present study, more effective measures that combine circadian rhythms with the diagnosis and treatment of psychosomatic diseases may improve psychological impacts and maximize the effectiveness of treatment. Therefore, the following measures are recommended for stabilizing daily routines, especially for those specific population who mostly suffered from disrupted rhythms: 1), self-management strategies for setting up and increasing regularity of daily activities and lifestyle habits during the COVID-19 pandemic[7]; 2), spending 1 hour/day outdoors, walking for more than one hour a day and basking in the sun, which may regulate the body clock by the light-dark cycle; 3), scheduling social interactions at the same time of the day, and seeking the best candidates who may share thoughts and feelings in real time[7]; 4), eating meals at the same time every day, and adjusting the eating patterns and food categories. Previous studies have shown that regular eating and consumption of various metabolites promote the periodic colonization of the intestinal bacteria on the mucosal surface, and alleviate various adverse mental problems through the intestinal flora-gut-brain axis[41]; 5), avoiding daylight hour naps and the nocturnal blue spectrum light pollution from computers, mobile phones, electronic tablets and

televisions, which suppresses the sleep-helping hormones[7]. Chronotherapy seems to be a promising approach to restore the proper circadian pattern in the elderly with an obvious sleep problem through adequate sleep hygiene, timed light exposure, and the use of a chronobiotic medication[12]; 6), taking drugs that adjust the biological clock for nurses with sleep disorders caused by nurses' shift system when necessary[42]; and 7), encouraging online therapy program for normalizing the disrupted social rhythms in COVID-19 environment, with the support of health care professionals and persuasive systems design and user experience/interaction features[7].

The present study provides new ideas for the simultaneous intervention from the perspective of social rhythms and psychology, and would lay an epidemiological foundation for the interaction of social rhythms with the diagnosis and treatment of psychosomatic diseases and the maximization of therapeutic efficacy. However, there are a few limitations in the present study. First, this cross-sectional study cannot reveal causality, and the nature of voluntary participation may result in selection bias. Second, in this study, we did not track the 24-hour circadian rhythm. Indeed, it is necessary to track the circadian rhythms/sleep-wake cycle by a multi-lead-sleep monitor considering different psychological impacts, which will be implemented in our further research. Third, the survey response rate was not obtained since this online-survey was participated voluntarily and the lockdown and social isolation policy did not encourage a face-to-face survey, and it was difficult to get the survey response rate. Fourth, we did not consider obtaining the individual informed consent for teenagers themselves under

18 years old, in addition to their parents' consent, which will also be implemented in our further similar research.

In conclusion, there exist rhythm disruptions among Chinese people with different sociodemographic backgrounds, which are closely associated with psychological impacts under the stress of the COVID-19 pandemic. Social rhythm disruption is independently associated with depression and anxiety. Interventions should be applied to the people vulnerable to the rhythm disruptions during the COVID-19 pandemic.

Acknowledgments

Zhihua Gao, PhD and Jinxia Zhu, PhD, provided valuable assistance with on questionnaire design. Kai Guo provided the on-line technical support. We are grateful for Medjaden Inc. for its assistance in the preparation of the manuscript and professional editing and language polishing.

Author Contributions

Min Yang, Ping He, Xiaoming Xu and Dan Li formulated the study design, carried out this study and wrote the manuscript. Jing Wang, Yanjun Wang and Mei Zhao collected

data and literature. Bin Wang and Wo Wang prepared the tables and format adjustment. Hui Lin and Ming-Ming Deng contributed to data analysis and data interpretation. Tianwei Deng, Li Kuang and Dongfeng Chen conceived the study and edited the manuscript.

References

1. Huang C, Wang Y, Li X, Ren L, Zhao J, Hu Y, et al. Clinical features of patients infected with 2019 novel coronavirus in Wuhan, China. Lancet (London, England). 2020;395(10223):497-506. Epub 2020/01/28. doi: 10.1016/s0140-6736(20)30183-5. PubMed PMID: 31986264; PubMed Central PMCID: PMCPMC7159299.

2. Li Q, Guan X, Wu P, Wang X, Zhou L, Tong Y, et al. Early Transmission Dynamics in Wuhan, China, of Novel Coronavirus-Infected Pneumonia. The New England journal of medicine. 2020;382(13):1199-207. Epub 2020/01/30. doi: 10.1056/NEJMoa2001316. PubMed PMID: 31995857; PubMed Central PMCID: PMCPMC7121484.

3. Zhou F, Yu T, Du R, Fan G, Liu Y, Liu Z, et al. Clinical course and risk factors for mortality of adult inpatients with COVID-19 in Wuhan, China: a retrospective cohort study. Lancet (London, England). 2020;395(10229):1054-62. Epub 2020/03/15. doi: 10.1016/s0140-6736(20)30566-3. PubMed PMID: 32171076; PubMed Central PMCID: PMCPMC7270627.

4. Bashir MF, Ma B, Shahzad L. A brief review of socio-economic and environmental impact of Covid-19. Air quality, atmosphere, & health. 2020:1-7. Epub 2020/08/25. doi: 10.1007/s11869-020-00894-8. PubMed PMID: 32837620; PubMed Central PMCID: PMCPMC7395801.

5. Pfefferbaum B, North CS. Mental Health and the Covid-19 Pandemic. The New

England journal of medicine. 2020;383(6):510-2. Epub 2020/04/14. doi: 10.1056/NEJMp2008017. PubMed PMID: 32283003.

6. Xiong J, Lipsitz O, Nasri F, Lui LMW, Gill H, Phan L, et al. Impact of COVID-19 pandemic on mental health in the general population: A systematic review. Journal of affective disorders. 2020;277:55-64. Epub 2020/08/18. doi: 10.1016/j.jad.2020.08.001. PubMed PMID: 32799105; PubMed Central PMCID: PMCPMC7413844.

7. Murray G, Gottlieb J, Swartz HA. Maintaining Daily Routines to Stabilize Mood: Theory, Data, and Potential Intervention for Circadian Consequences of COVID-19. Can J Psychiatry. 2020:706743720957825. Epub 2020/09/11. doi: 10.1177/0706743720957825. PubMed PMID: 32909832.

8. Crowe M, Inder M, Swartz HA, Murray G, Porter R. Social rhythm therapy-A potentially translatable psychosocial intervention for bipolar disorder. Bipolar disorders. 2020;22(2):121-7. Epub 2019/09/21. doi: 10.1111/bdi.12840. PubMed PMID: 31539451.

9. Haynes PL, Gengler D, Kelly M. Social Rhythm Therapies for Mood Disorders: an Update. Current psychiatry reports. 2016;18(8):75. Epub 2016/06/25. doi: 10.1007/s11920-016-0712-3. PubMed PMID: 27338753; PubMed Central PMCID: PMCPMC4919368.

10. Kim BN. Behavioral activation system (BAS) dysregulation and bipolar spectrum

psychopathology in daily life: An online-diary study. Psychiatry research. 2020;291:113180. Epub 2020/06/17. doi: 10.1016/j.psychres.2020.113180. PubMed PMID: 32544707.

11. Majumdar P, Biswas A, Sahu S. COVID-19 pandemic and lockdown: cause of sleep disruption, depression, somatic pain, and increased screen exposure of office workers and students of India. Chronobiology international. 2020;37(8):1191-200. Epub 2020/07/15. doi: 10.1080/07420528.2020.1786107. PubMed PMID: 32660352.

12. Cardinali DP, Brown GM, Reiter RJ, Pandi-Perumal SR. Elderly as a High-risk Group during COVID-19 Pandemic: Effect of Circadian Misalignment, Sleep Dysregulation and Melatonin Administration. Sleep and vigilance. 2020:1-7. Epub 2020/10/06. doi: 10.1007/s41782-020-00111-7. PubMed PMID: 33015537; PubMed Central PMCID: PMCPMC7519696.

13. Rodrigues Aguilar AC, Frange C, Huebra L, Dias Gomes AC, Tufik S, Santos Coelho FM. The effects of the COVID-19 pandemic on patients with narcolepsy. Journal of clinical sleep medicine : JCSM : official publication of the American Academy of Sleep Medicine. 2020. Epub 2020/10/31. doi: 10.5664/jcsm.8952. PubMed PMID: 33124977.

14. Türkoğlu S, Uçar HN, Çetin FH, Güler HA, Tezcan ME. The relationship between chronotype, sleep, and autism symptom severity in children with ASD in COVID-

19 home confinement period. Chronobiology international. 2020;37(8):1207-13. Epub 2020/08/05. doi: 10.1080/07420528.2020.1792485. PubMed PMID: 32746638.

15. Margraf J, Lavallee K, Zhang X, Schneider S. Social Rhythm and Mental Health: A Cross-Cultural Comparison. PLoS One. 2016;11(3):e0150312. Epub 2016/03/10. doi: 10.1371/journal.pone.0150312. PubMed PMID: 26954568; PubMed Central PMCID: PMCPMC4783111.

16. Gao ZH, Yang SQ, Margraf J, Zhang XC. Reliability and Validity for Rhythm Scale in Chinese. China journal of health psychology 2013;21(08):1219-21. (in Chinese) http://med.wanfangdata.com.cn/Paper/Detail/PeriodicalPaper_jkxlxzz201308042

17. Zung WW. A SELF-RATING DEPRESSION SCALE. Archives of general psychiatry. 1965;12:63-70. Epub 1965/01/01. doi: 10.1001/archpsyc.1965.01720310065008. PubMed PMID: 14221692.

18. Zung WW. A rating instrument for anxiety disorders. Psychosomatics. 1971;12(6):371-9. Epub 1971/11/01. doi: 10.1016/s0033-3182(71)71479-0. PubMed PMID: 5172928.

19. Peng S, Zhang YY, Ji Y, Tang WQ, Li Q, Yan XL, et al. Analysis of reliability and validity of Chinese version SDS Scale in women of rural area. Shanghai medical & pharmaceutical journal 2013;34(14):20-3. (in Chinese) http://med.wanfangdata.com.cn/Paper/Detail/PeriodicalPaper_shyy201314011

20. Hühne A, Welsh DK, Landgraf D. Prospects for circadian treatment of mood disorders. Annals of medicine. 2018;50(8):637-54. Epub 2018/09/29. doi: 10.1080/07853890.2018.1530449. PubMed PMID: 30265156.

21. Lyall LM, Wyse CA, Graham N, Ferguson A, Lyall DM, Cullen B, et al. Association of disrupted circadian rhythmicity with mood disorders, subjective wellbeing, and cognitive function: a cross-sectional study of 91 105 participants from the UK Biobank. The lancet Psychiatry. 2018;5(6):507-14. Epub 2018/05/20. doi: 10.1016/s2215-0366(18)30139-1. PubMed PMID: 29776774.

22. Bailey M, Silver R. Sex differences in circadian timing systems: implications for disease. Frontiers in neuroendocrinology. 2014;35(1):111-39. Epub 2013/11/30. doi: 10.1016/j.yfrne.2013.11.003. PubMed PMID: 24287074; PubMed Central PMCID: PMCPMC4041593.

23. Grafe LA, Bhatnagar S. The contribution of orexins to sex differences in the stress response. Brain Res. 2020;1731:145893. Epub 2018/08/07. doi: 10.1016/j.brainres.2018.07.026. PubMed PMID: 30081036; PubMed Central PMCID: PMCPMC6360123.

24. Schubert CC, Punamäki RL. Mental health among torture survivors: cultural background, refugee status and gender. Nordic journal of psychiatry. 2011;65(3):175-82. Epub 2010/09/22. doi: 10.3109/08039488.2010.514943. PubMed PMID: 20854222.

25. Pieh C, Budimir S, Probst T. The effect of age, gender, income, work, and physical activity on mental health during coronavirus disease (COVID-19) lockdown in Austria. Journal of psychosomatic research. 2020;136:110186. Epub 2020/07/19. doi: 10.1016/j.jpsychores.2020.110186. PubMed PMID: 32682159.

26. Koritala BSC, Çakmaklı S. The human circadian clock from health to economics. PsyCh journal. 2018;7(4):176-96. Epub 2018/11/21. doi: 10.1002/pchj.252. PubMed PMID: 30456783.

27. Groarke JM, Berry E, Graham-Wisener L, McKenna-Plumley PE, McGlinchey E, Armour C. Loneliness in the UK during the COVID-19 pandemic: Cross-sectional results from the COVID-19 Psychological Wellbeing Study. PLoS One. 2020;15(9):e0239698. Epub 2020/09/25. doi: 10.1371/journal.pone.0239698. PubMed PMID: 32970764; PubMed Central PMCID: PMCPMC7513993.

28. Nguyen C, Murray G, Anderson S, Filipowicz A, Ingram KK. In vivo molecular chronotyping, circadian misalignment, and high rates of depression in young adults. Journal of affective disorders. 2019;250:425-31. Epub 2019/03/18. doi: 10.1016/j.jad.2019.03.050. PubMed PMID: 30878655.

29. Liu WH, Zhou JY, Guo XY, Wu BY, Yu Q, Dong HL. Impact analysis of lifestyle on the health status of the elderly. Chinese primary health care 2018;32(10):29-31. (in Chinese) http://med.wanfangdata.com.cn/Paper/Detail/PeriodicalPaper_zgcjwsbj201810011

30. Lananna BV, Musiek ES. The wrinkling of time: Aging, inflammation, oxidative stress, and the circadian clock in neurodegeneration. Neurobiology of disease. 2020;139:104832. Epub 2020/03/18. doi: 10.1016/j.nbd.2020.104832. PubMed PMID: 32179175; PubMed Central PMCID: PMCPMC7727873.

31. Burdelak W, Peplońska B. [Night work and health of nurses and midviwes--a review]. Medycyna pracy. 2013;64(3):397-418. Epub 2013/11/23. PubMed PMID: 24261252.

32. McElroy SF, Olney A, Hunt C, Glennon C. Shift work and hospital employees: A descriptive multi-site study. International journal of nursing studies. 2020;112:103746. Epub 2020/09/16. doi: 10.1016/j.ijnurstu.2020.103746. PubMed PMID: 32928504.

33. Rosa D, Terzoni S, Dellafiore F, Destrebecq A. Systematic review of shift work and nurses' health. Occupational medicine (Oxford, England). 2019;69(4):237-43. Epub 2019/05/28. doi: 10.1093/occmed/kqz063. PubMed PMID: 31132107.

34. Grandin LD, Alloy LB, Abramson LY. The social zeitgeber theory, circadian rhythms, and mood disorders: review and evaluation. Clinical psychology review. 2006;26(6):679-94. Epub 2006/08/15. doi: 10.1016/j.cpr.2006.07.001. PubMed PMID: 16904251.

35. Healy D. Rhythm and blues. Neurochemical, neuropharmacological and neuropsychological implications of a hypothesis of circadian rhythm dysfunction in

the affective disorders. Psychopharmacology. 1987;93(3):271-85. Epub 1987/01/01. doi: 10.1007/bf00187243. PubMed PMID: 3124158.

36. Landgraf D, Long JE, Proulx CD, Barandas R, Malinow R, Welsh DK. Genetic Disruption of Circadian Rhythms in the Suprachiasmatic Nucleus Causes Helplessness, Behavioral Despair, and Anxiety-like Behavior in Mice. Biological psychiatry. 2016;80(11):827-35. Epub 2016/04/27. doi: 10.1016/j.biopsych.2016.03.1050. PubMed PMID: 27113500; PubMed Central PMCID: PMCPMC5102810.

37. Kinlein SA, Karatsoreos IN. The hypothalamic-pituitary-adrenal axis as a substrate for stress resilience: Interactions with the circadian clock. Frontiers in neuroendocrinology. 2020;56:100819. Epub 2019/12/22. doi: 10.1016/j.yfrne.2019.100819. PubMed PMID: 31863788; PubMed Central PMCID: PMCPMC7643247.

38. Fiksdal A, Hanlin L, Kuras Y, Gianferante D, Chen X, Thoma MV, et al. Associations between symptoms of depression and anxiety and cortisol responses to and recovery from acute stress. Psychoneuroendocrinology. 2019;102:44-52. Epub 2018/12/05. doi: 10.1016/j.psyneuen.2018.11.035. PubMed PMID: 30513499; PubMed Central PMCID: PMCPMC6420396.

39. Silva A, Dos Santos MJ, Góes Gitaí DL, de Miranda Coelho JAP, de Andrade TG. Depression and anxiety symptoms correlate with diurnal preference, sleep habits,

and Per3 VNTR polymorphism (rs57875989) in a non-clinical sample. Journal of affective disorders. 2020;277:260-70. Epub 2020/08/26. doi: 10.1016/j.jad.2020.07.138. PubMed PMID: 32841827.

40. Mathew GM, Li X, Hale L, Chang AM. Sleep duration and social jetlag are independently associated with anxious symptoms in adolescents. Chronobiology international. 2019;36(4):461-9. Epub 2019/02/23. doi: 10.1080/07420528.2018.1509079. PubMed PMID: 30786775; PubMed Central PMCID: PMCPMC6397070.

41. Kaczmarek JL, Thompson SV, Holscher HD. Complex interactions of circadian rhythms, eating behaviors, and the gastrointestinal microbiota and their potential impact on health. Nutrition reviews. 2017;75(9):673-82. Epub 2017/09/25. doi: 10.1093/nutrit/nux036. PubMed PMID: 28938796; PubMed Central PMCID: PMCPMC5914376.

42. Pevet P, Challet E. Melatonin: both master clock output and internal time-giver in the circadian clocks network. Journal of physiology, Paris. 2011;105(4-6):170-82. Epub 2011/09/15. doi: 10.1016/j.jphysparis.2011.07.001. PubMed PMID: 21914478.

Supporting Information

S1 Fig. Correlations of SGS-scale with SDS (A) and SAS (B)

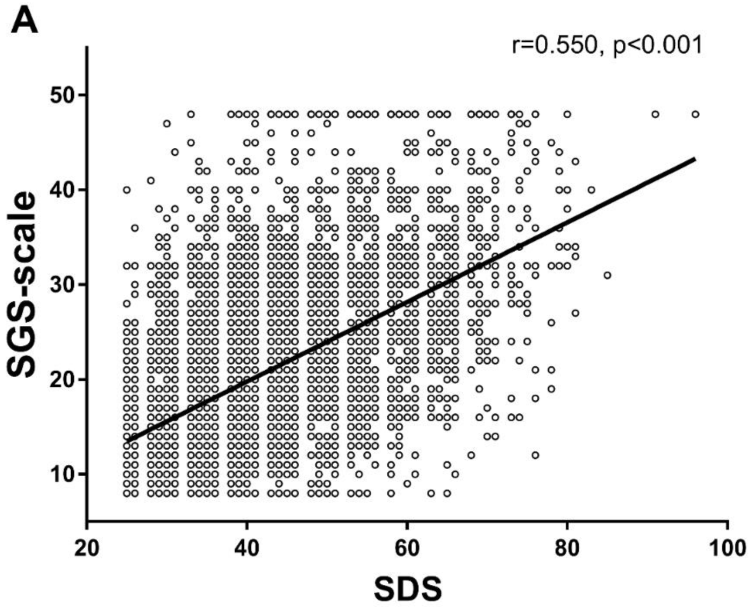

A

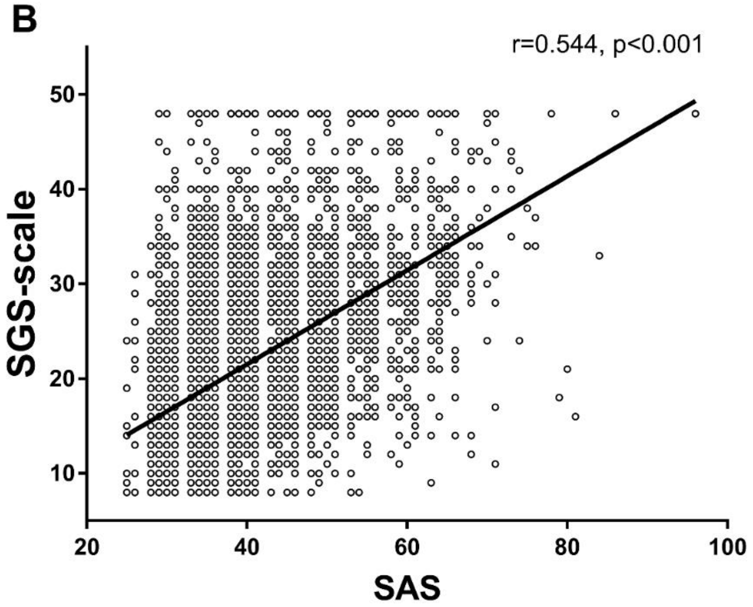

B

SGS-scale: the rhythm of sleep, getting-up, and socializing; SDS: Zung's self-rate depression scale; SAS: Zung's self-rate anxiety scale.

S1 Table. Prevalence of depression with different grades in participants (N=5854).

	N	Normal N=4430	Mild N=897	Moderate N=402	Severe N=125	*P*
**Health status**						
Poor	53	6(11.32%)	16(30.19%)	16(30.19%)	15(28.30%)	<0.001
Normal	787	410(52.10%)	204(25.92%)	121(15.37%)	52(6.61%)	
Good	3686	2849(77.29%)	576(15.63%)	218(5.91%)	43(1.17%)	
Very good	1328	1165(87.73%)	101(7.61%)	47(3.54%)	15(1.13%)	
**Current occupation**						
Businessman	481	388(80.67%)	64(13.31%)	25(5.20%)	4(0.83%)	<0.001
Officer	245	201(82.04%)	39(15.92%)	3(1.22%)	2(0.82%)	
Teacher	231	183(79.22%)	35(15.15%)	5(2.16%)	8(3.46%)	
Police	277	234(84.48%)	25(9.03%)	15(5.42%)	3(1.08%)	
Farmer	217	159(73.27%)	39(17.97%)	14(6.45%)	5(2.30%)	
Employee	270	208(77.04%)	47(17.41%)	12(4.44%)	3(1.11%)	
Doctor	1171	828(70.71%)	188(16.05%)	115(9.82%)	40(3.42%)	
Nurse	1017	682(67.06%)	186(18.29%)	113(11.11%)	36(3.54%)	
Medical technician	139	100(71.94%)	23(16.55%)	10(7.19%)	6(4.32%)	
Retired re-employee	229	174(75.98%)	42(18.34%)	9(3.93%)	4(1.75%)	
Non-medical student	1172	984(83.96%)	131(11.18%)	49(4.18%)	8(0.68%)	
Medical student	405	289(71.36%)	78(19.26%)	32(7.90%)	6(1.48%)	
**Chronic disease**						

No chronic disease	4373	3519(80.47%)	566(12.94%)	233(5.33%)	55(1.26%)	<0.001
CDCPD	671	363(54.10%)	171(25.48%)	90(13.41%)	47(7.00%)	
Chronic diseases only	810	548(67.65%)	160(19.75%)	79(9.75%)	23(2.84%)	

SDS, Zung's self-rating depression scale; CY, Chinese yuan; CDCPD, Chronic diseases comorbid with psychosomatic diseases.

S2 Table. Prevalence of anxiety with different grades in participants (N=5854).

	N	Normal	Mild	Moderate and severe	*P*
		N=5114	N=513	N=227	
**Health status**					
Poor	53	17(32.08%)	18(33.96%)	18(33.96%)	<0.001
Normal	787	550(69.89%)	161(20.46%)	76(9.66%)	
Good	3686	3297(89.45%)	287(7.79%)	102(2.77%)	
Very good	1328	1250(94.13%)	47(3.54%)	31(2.33%)	
**Current occupation**					
Businessman	481	438(91.06%)	31(6.44%)	12(2.49%)	<0.001
Officer	245	228(93.06%)	14(5.71%)	3(1.22%)	
Teacher	231	203(87.88%)	21(9.09%)	7(3.03%)	
Police	277	252(90.97%)	19(6.86%)	6(2.17%)	
Farmer	217	195(89.86%)	14(6.45%)	8(3.69%)	
Employee	270	240(88.89%)	25(9.26%)	5(1.85%)	
Doctor	1171	961(82.07%)	130(11.10%)	80(6.83%)	
Nurse	1017	824(81.02%)	129(12.68%)	64(6.29%)	
Medical technician	139	119(85.61%)	13(9.35%)	7(5.04%)	
Retired re-employee	229	200(87.34%)	23(10.04%)	6(2.62%)	
Non-medical student	1172	1105(94.28%)	53(4.52%)	14(1.19%)	
Medical student	405	349(86.17%)	41(10.12%)	15(3.70%)	
**Chronic disease**					

No chronic disease	4373	4004(91.56%)	262(5.99%)	107(2.45%)	<0.001
CDCPD	671	460(68.55%)	136(20.27%)	75(11.18%)	
Chronic diseases only	810	650(80.25%)	115(14.20%)	45(5.56%)	

SAS, Zung's self-rating anxiety scale; CY, Chinese yuan; CDCPD, Chronic diseases comorbid with psychosomatic diseases.

S3 Table. Spearman's correlation between subscale 1, subscale 2, Zung's self-rating depression scale (SDS)and Zung's self-rating anxiety scale (SAS). (N=5854)

Outcomes	Subscale1	Subscale2	SDS	SAS
**Subscale1**	-	0.058**	0.550**	0.544**
**Subscale2**	-	-	-0.037**	0.008
**SDS**	-	-	-	0.808**
**SAS**	-	-	-	-

**, *P*<0.001.

S1A Fig

Click here to access/download
Supporting Information
renamed_2e959.tif

S1B Fig

Click here to access/download
Supporting Information
renamed_dba10.tif

Click here to access/download
Other
Informed consent 2021-1-21.docx

Disrupted rhythms of life, work and entertainment and their associations with psychological impacts under the stress of the COVID-19 pandemic: A Survey in 5854 Chinese people with different sociodemographic backgrounds

Min Yang*[1], Ping He*[2], Xiaoming Xu*[3], Dan Li*[4], Jing Wang[1], Yanjun Wang[1], Bin Wang[1], Wo Wang[5], Mei Zhao[4], Hui Lin[6], Mingming Deng[#7], Tianwei Deng[#8], Li Kuang[#3], Dongfeng Chen [#1]

[1]Army Medical Center of PLA, Daping Hospital, Army Medical University, 10 Changjiang Branch Road, Daping, Yuzhong District, Chongqing 400042, P.R. China;

[2]Department of Gastroenterology, Yongchuan Hospital of Chongqing Medical University, 439 Xuanhua Road, Yongchuan District, Chongqing 402160, P.R. China;

[3]Department of Psychiatry, the First Affiliated Hospital of Chongqing Medical University, 1 Youyi Road, Yuzhong District, Chongqing 400016, P.R. China;

[4] CAS Key Laboratory of Mental Health, Institute of Psychology, 16 Lincui Road, Chao Yang District, Beijing 100101, P.R. China;

[5] Mental Health Center, University-Town Hospital of Chongqing Medical University, 55 Middle Road, Shapingba District, Chongqing 401331, P.R. China;

[6] Department of Statistics, Army Medical University, 30 Gaotanyan Street, Shapingba District Chongqing 400038, P.R. China;

[7] Department of Gastroenterology, The Affiliated Hospital of Southwest Medical University, 25 Taiping Street, Jiangyang District, Luzhou 64600, P.R. China

[8] Department of Gastroenterology, Three Gorges Hospital of Chongqing University, 165 Xincheng Road, Wanzhou, Chongqing 404000, P.R. China;

[#]Corresponding authors: Dongfeng Chen, Li Kuang, Tianwei Deng, Mingming Deng.

Email:
chendf1981@126.com(CDF);kuangli0308@163.com(LK);dengtw1986@163.com(DTW);793070544@qq.com(DMM).

* These authors contributed equally to this work.

# These authors also contributed equally to this work.

Data Availability Statement: All relevant data are within the paper and its Supporting Information files.

Competing Interests: The authors have declared that no competing interests exist.

Abstract

Background & Aim The coronavirus disease 2019 (COVID-19) pandemic has affected the life and work of people worldwide. The present study aimed to evaluate the rhythm disruptions of life, work, and entertainment, and their associations with the psychological impacts during the initial phase of the COVID-19 pandemic.

Method A cross-sectional study was conducted from the 10th to 17th March 2020 in China. A structured e-questionnaire containing general information, the Chinese version of Brief Social Rhythm Scale, and Zung's self-rating scales of depression and anxiety (SDS and SAS) was posted and collected online through a public media (*i.e.* EQxiu online questionnaire platform). Scores in sleeping, getting up, and socializing (SGS) rhythm and eating, physical practice, and entertainment (EPE) rhythm were compared among and between participants with different sociodemographic backgrounds including gender, age, education, current occupation, annual income, health status, and chronic disease status. Correlations of SDS and SAS with SGS-scale and EPE-scale were analyzed.

Results Overall, 5854 participants were included. There were significant differences in the scores of SGS-scale and EPE-scale among people with different sociodemographic backgrounds. The scores were significantly higher in the groups

with female gender, low education level, too-low or too-high income, poor health status, ages of 26-30 years or older than 61 years, nurses and subjects with divorce or widow status. There were also significant differences in SAS and SDS scores among people with different sociodemographic backgrounds (all $P < 0.05$). The overall prevalence of depression and anxiety was 24.3% and 12.6%, respectively, with nurses having the highest rates of depression (32.94%) and anxiety (18.98%) among the different occupational groups. SGS-scale was moderately correlated with SDS and SAS, and disruption of SGS rhythm was an independent risk factor for depression and anxiety.

Conclusion Social rhythm disruption was independently associated with depression and anxiety. Interventions should be applied to people vulnerable to the rhythm disruption during the COVID-19 pandemic.

Key words: Coronavirus disease 2019 (COVID-19); Social rhythm disruption; Anxiety and depression; Psychological impacts; Chinese people.

Introduction

Coronavirus disease 2019 (COVID-19) is an acute respiratory infectious disease caused by a novel coronavirus, called severe acute respiratory syndrome coronavirus 2 (SARS-CoV-2)[1-3]. Due to the strong infectivity of the coronavirus and the lack of effective treatments and vaccines, COVID-19 has spread rapidly and become a pandemic worldwide with serious consequences. The social restriction policies implemented to control the infection in almost all countries have resulted in unemployment, financial hardship, and a reduction in quality of life in many countries (Bashir, M. F. 2020)[4], which have seriously affected the life and work of people. Simultaneously, the COVID-19 pandemic has alarming implications for a global increase in mental health problems, and the psychological burden during the COVID-19 pandemic has been reported[5, 6].

Social rhythm is defined as the regularity of timing of the behaviors in social activities and also described clinically as "daily routines"[7]. COVID-19-related social changes, particularly lockdown protocols, have indeed impacted the timing of daily behaviors in stabilizing circadian function, and social rhythm disruption may be correlated with psychological impacts[7]. Previous studies have reported that social rhythm disruption is connected with mental diseases, especially depression and bipolar disorder in the non-pandemic days, which has been explained by many theories[8-11]. Recently, several studies have reported social and circadian rhythm disruptions in students, office workers, the elder, and patients with narcolepsy or autism[12-14].

However, most of these studies mainly concentrated on sleep disruption, and none of them investigated the disrupted rhythms of life, work and entertainment behaviors and their associations with psychological impacts under the stress of the COVID-19 pandemic. We hypothesize that people with different sociodemographic backgrounds have different degrees of rhythm disruption in life, work, and entertainment, that the rhythm disruption is more severe in some specific groups, and that the rhythm disruption is associated with psychological impacts during the initial phase of the COVID-19 pandemic. Therefore, the aim of the present study was to evaluate the rhythm disruptions in life, work, and entertainment and their associations with the psychological impacts in the general Chinese population with different sociodemographic backgrounds during the initial phase of the COVID-19 pandemic.

Materials and Methods

Participants and procedure

Chinese people with different sociodemographic backgrounds voluntarily participated in this online survey. An e-questionnaire was designed to investigate the rhythm activities and psychological impacts in Chinese people with different backgrounds including gender, age, educational background, annual income, health status, current occupation, and chronic disease status during the COVID-19 pandemic through EQxiu online questionnaire platform. The collection method ~~was~~ complied with the terms and

conditions for the website. The survey was conducted from 10-17 March 2020 in 22 provinces and five autonomous regions, four municipalities, and two special administrative regions (*i.e.*, Hong Kong and Macao) of China. The study protocol was carried out following relevant guidelines and regulations and approved by the Ethics Committee of the Army Medical Center (Approval No. 2019-144). During the survey, the participants were asked to read and check the informed consent and then complete the questionnaire; those under 18 years old were asked to consent by their guardians before completing the questionnaire. All completed questionnaires were ~~received and~~ checked for validity and completeness.

Measures

Social rhythm. The Brief Social Rhythm Scale (BSRS) was developed to quickly assess social rhythmicity in large-scale multi-national samples and multiple languages[15]. The Chinese version of BSRS also included entertainment and physical practice, and was proved to have good reliability and validity in China[16]. The scale consisting of 17 items was used to investigate an individual's rhythm in six aspects, i.*e.*, sleeping (going to bed), getting up, socializing (SGS), eating, physical practice, and entertainment (EPE) on weekdays and weekends. The first eight items addressing SGS rhythm constituted SGS-scale, and the last nine items addressing EPE rhythm constituted EPE-scale. Each item was assigned with a score of 1, 2, 3, 4, 5, or 6,

corresponding to categories of very regularly, quite regularly, somewhat regularly, somewhat irregularly, quite irregularly, or very irregularly, respectively. The lower the score ~~was~~, the more regular the rhythm ~~was~~.

Zung's depression and anxiety scale. Zung's self-rating depression scale (SDS) and Zung's self-rating anxiety scale (SAS) were used to evaluate the psychological impacts of each participant[17, 18]. There were 20 items in SAS and SDS, and each item was graded to 1-4 reflecting never, often, sometimes, and always. The rough score was the sum of 20 questions multiplied by 1.25 and rounded to get the standard score. The cut-off value was 50 for both SAS and SDS. The higher the score, the more obvious the anxiety or depression was, with the scores of 50-59, 60-69, and 70 points or more indicating mild, moderate, and severe anxiety or depressive symptom, respectively. The Chinese version of SDS and SAS, which was used in the present study, was reported to present good reliability, validity and excellent internal consistency, with the Cronbach coefficient of 0.784 for SDS in Chinese people[19], and of 0.931 for SAS in the general population[20].

Statistical analysis

Data were analyzed using SPSS version 23.0 (SPSS, Chicago, IL, USA). The data of normal distribution was expressed by mean±standard deviation. One-way analysis of variance with a post-hoc analysis, was used to make comparisons among groups. T-test

or a nonparametric test (*i.e.*, Mann-Whitney U test or Kruskal-Wallis test), where appropriate, was used to make comparisons between the group with the highest score and each of the other groups for each item. Pearson correlation or Spearman correlation, where appropriate, was used to determine the correlation between the ~~scales of social rhythm scale~~ and the Zung's scales. In addition, a multivariate linear regression analysis was used to determine the independent factors for the correlation of anxiety or depression with social rhythm. A *P* value of < 0.05 was considered statistically significant.

Results

Sociodemographic characteristics of the participants

A total of 5872 people checked the e-questionnaire. Eighteen questionnaires with invalid were excluded ~~the data analysis~~ due to frivolous or incomplete answers, or the same option for different questions or orderly answers. The data of 5854 participants with valid questionnaires were further analyzed. The general information on the sociodemographic characteristics of the participants is listed in Table 1.

Table 1. General sociodemographic characteristics of 5854 analyzed participants

Feature		N (%)	Feature		N (%)
Gender	Female	3439(58.75%)	Health status	Poor	53(0.91%)
	Male	2415(41.25%)		Normal	787(13.44%)

Age	-18	68(1.16%)		Good	3686(62.97%)
	18-25	1960(33.48%)		Very good	1328(22.69%)
	26-30	862(14.72%)	Current occupation	Businessman	481(8.22%)
	31-40	1339(22.87%)		Officer	245(4.19%)
	41-50	852(14.55%)		Teacher	231(3.95%)
	51-60	682(11.65%)		Police	277(4.73%)
	61-	91(1.55%)		Farmer	217(3.71%)
Marital status	Unmarried	2435(41.60%)		Employee	270(4.61%)
	Married	3232(55.21%)		Doctor	1171(20.00%)
	Divorced /widowed	187(3.19%)		Nurse	1017(17.37%)
Chronic disease	No chronic disease	4373(74.70%)		Medical technician	139(2.37%)
	CDCPD	671(11.46%)		Retired re-employee	229(3.91%)
	Chronic diseases only	810(13.84%)		Non-medical student	1172(20.02%)
Annual income	Less than 50000 CY	2482(42.40%)		Medical student	405(6.92%)
	50000-100000 CY	1849(31.59%)	Education	Junior high school or lower	97(1.66%)
	100000-200000 CY	1188(20.29%)		Senior high school	535(9.14%)
	200000-300000 CY	225(3.84%)		Junior college or undergraduate	4465(76.27%)
	More than 300000 CY	110(1.88%)		Master or above	757(12.93%)

CY, Chinese yuan; CDCPD, chronic diseases comorbid with psychosomatic diseases.

Disruptions of social rhythm in the participants

Generally, there were significant differences in SGS-scale and EPE-scale among participants with different sociodemographic backgrounds including gender, age,

educational background, annual income, health status, current occupation, and chronic disease status (all $P<0.05$, Table 2). Moreover, the SGS-scale and EPE-scale scores were compared between the group with the highest score and each of other groups (Table 3). Specifically, the female gender and poor health status were most closely associated with disrupted rhythms of life, work, and entertainment. The age group of 26-30 years, nurses and subjects with divorce or widow status, the education level of senior high school, annual pre-tax income of 50,000-100,000 Chinese Yuan (CY), or chronic disease comorbid with psychosomatic diseases mostly suffered from disrupted rhythms of life and work. The age group of older than 61, and subjects with education levels of master's degree or above, annual pre-tax income over 300,000 CY, or chronic disease without psychosomatic diseases mostly suffered from disrupted rhythms of entertainment. Especially, nurses showed significantly more severe disruption of SGS rhythm, whereas the elderly reported significant irregularity in EPE rhythm (Table 2).

Table 2. Scores of social rhythms in participants with different sociodemographic backgrounds during the COVID-19 pandemic (N=5854).

Feature		SGS-scale			EPE-scale		
		Mean±SD	Z/X2	*P*	Mean±SD	Z/X2	*P* value
Gender	Female	21.64±9.03	-10.183	<0.001	7.71±6.02	-7.627	<0.001
	Male	19.24±8.80			8.71±5.93		
Age	-18	17.63±8.69	146.237	<0.001	7.82±5.58	112.103	<0.001
	18-25	18.98±8.95			7.75±5.35		

	26-30	23.04±9.77			7.22±6.22		
	31-40	21.54±8.95			7.76±6.18		
	41-50	20.79±8.56			9.10±6.68		
	51-60	20.74±7.91			9.64±5.82		
	61-	21.27±8.78			9.87±6.14		
Marital status	Unmarried	19.53±9.19	81.065	<0.001	7.81±5.52	2.499	0.294
	Married	21.42±8.77			8.32±6.26		
	Divorced /widowed	22.04±9.21			8.76±7.25		
Education	Junior high school or lower	18.92±10.07	8.880	0.031	7.81±7.18	11.172	0.011
	Senior high school	21.18±8.97			7.75±5.97		
	Junior college or undergraduate	20.64±9.06			8.05±5.89		
	Master or above	20.59±8.61			8.85±6.46		
Annual income	Less than 50000 CY	19.21±8.90	126.387	<0.001	7.88±5.64	48.455	<0.001
	50000-100000 CY	22.18±9.26			7.68±6.06		
	100000-200000 CY	21.28±8.49			8.93±6.28		
	200000-300000 CY	20.66±8.41			9.31±6.03		
	More than 300000 CY	20.78±9.03			9.86±8.12		
Health status	Poor	28.02±10.00	500.564	<0.001	9.53±8.85	26.969	<0.001
	Normal	25.21±9.48			7.94±7.19		
	Good	20.93±8.36			8.35±5.90		
	Very good	16.90±8.85			7.53±5.30		
Current occupation	Businessman	20.21±8.50	322.663	<0.001	8.39±5.92	177.273	<0.001
	Officer	19.89±8.18			9.32±5.94		
	Teacher	21.06±8.41			9.74±6.77		

	Police	18.69±8.79			9.05±5.46		
	Farmer	19.81±9.55			7.47±6.17		
	Employee	20.49±7.99			8.44±5.81		
	Doctor	20.99±8.27			8.52±6.39		
	Nurse	24.47±9.91			6.46±6.08		
	Medical technician	21.31±8.65			7.63±6.56		
	Retired re-employee	21.63±8.78			9.62±5.28		
	Non-medical student	17.65±8.46			7.90±5.20		
	Medical student	20.64±8.80			8.66±6.08		
Chronic disease	No chronic disease	19.71±8.82	202.421	<0.001	7.94±5.67	6.269	0.044
	CDCPD	24.01±9.18			8.67±7.40		
	Chronic diseases only	22.98±8.82			8.68±6.37		

SD, standard deviation; CY, Chinese yuan; SGS-scale: the rhythm of sleep, getting-up, and socializing; EPE-scale: the rhythm of eating, physical practice, and entertainment activities; CDCPD, Chronic diseases comorbid with psychosomatic diseases.

Table 3. Comparison of SGS-scale and EPE-scale scores between the group with the highest score and each of the other groups in terms of sociodemographic backgrounds

Features	SGS-scale			EPE-scale		
	Highest-score group	Other groups	*P*-value	Highest-score group	Other groups	*P*-value
Marital status	Divorced /widowed	Unmarried	0.001	Divorced /widowed	Unmarried	0.227
		Married	0.748		Married	0.802

Chronic disease	CDCPD	No chronic disease	<0.001	Chronic diseases only	No chronic disease	0.006
		Chronic diseases only	0.086		CDCPD	1.000
Education	Senior high school	Junior high school or lower	0.222	Master or above	Junior high school or lower	0.695
		Junior college or undergraduate	0.720		Senior high school	0.010
		Master or above	0.811		Junior college or undergraduate	0.009
Health status	Poor	Normal	0.275	Poor	Normal	0.749
		Good	<0.001		Good	0.918
		Very good	<0.001		Very good	0.499
Annual income	50000-100000 CY	Less than 50000 CY	<0.001	More than 300000 CY	Less than 50000 CY	0.119
		100000-200000 CY	0.056		50000-100000 CY	0.063
		200000-300000 CY	0.113		100000-200000 CY	0.938
		More than 300000 CY	0.714		200000-300000 CY	0.999
Age	26-30	-18	<0.001	61-	-18	0.473
		18-25	<0.001		18-25	0.034
		31-40	0.006		26-30	0.003
		41-50	<0.001		31-40	0.042
		51-60	<0.001		41-50	0.998
		61-	0.799		51-60	1.000
Current occupation	Nurse	Businessman	<0.001	Teacher	Businessman	0.482
		Officer	<0.001		Officer	1.000
		Teacher	<0.001		Police	1.000
		Police	<0.001		Farmer	0.015
		Farmer	<0.001		Employee	0.781

Employee	<0.001	Doctor	0.551
Doctor	<0.001	Nurse	<0.001
Medical technician	0.007	Medical technician	0.199
Retired re-employee	0.001	Retired re-employee	1.000
Non-medical student	<0.001	Non-medical student	0.008
Medical student	<0.001	Medical student	0.953

CY, Chinese yuan; SGS-scale: the rhythm of sleep, getting-up, and socializing; EPE-scale: the rhythm of eating, physical practice, and entertainment activities; CDCPD, Chronic diseases comorbid with psychosomatic diseases.

Psychological impacts in the participants

There were significant differences in SAS and SDS scores among participants with different genders, ages, marital status, education levels, annual pre-tax incomes, health status, current occupation, and chronic diseases with or without psychosomatic diseases in each of the variables (all $P<0.05$) (Table 4). Specifically, nurses and participants with female gender, divorce or widow status, education levels of junior high school or below, poor health status, and chronic diseases comorbid with psychosomatic diseases mostly suffered from depression and anxiety. Whereas the age group of 26-30 years, and participants with annual pre-tax income of 50,000-100,000 CY mostly suffered from depression, the age group of 31-40 years and participants with annual pre-tax income over 300,000 CY mostly suffered from anxiety. Additionally, SDS scores were

significantly higher in medical students than in non-medical students ~~(*P*<0.001)~~. There were significant differences in the prevalence of depression and anxiety among participants with different sociodemographic backgrounds (all *P*<0.05, S1 and S2 Tables). The overall prevalence of depression and anxiety was 24.33% and 12.64%, respectively, as defined by SDS or SAS scores over 50. Among the different occupational groups, nurses had the highest rates of depression (32.94%) and anxiety (18.98%). Participants with chronic diseases combined with psychosomatic diseases had the highest rates of depression (45.90%) and anxiety (31.45%).

Table 4. Scores of depression and anxiety in participants with different sociodemographic backgrounds during the COVID-19 pandemic (N=5854).

Feature		SDS			SAS		
		Mean±SD	Z/X2	*P*	Mean±SD	Z/X2	*P*
Gender	Female	43.34±11.66		<0.001	38.8±9.39		<0.001
	Male	39.97±11.59			37.19±9.23		
Age	-18	39.34±10.93		<0.001	35.09±8.35		<0.001
	18-25	40.13±11.63			35.82±8.27		
	26-30	43.87±12.20			39.66±9.97		
	31-40	43.59±11.68			39.84±9.76		
	41-50	42.48±11.89			39.4±9.94		
	51-60	41.27±10.76			38.12±8.61		
	61-	41.03±10.97			38.91±9.32		
Marital	Unmarried	40.65±11.79		<0.001	36.26±8.56		<0.001

status	Married	42.78±11.60		39.39±9.61	
	Divorced /widowed	44.57±12.05		40.87±10.63	
Education	Junior high school or lower	45.22±13.29	<0.05	39.85±10.43	<0.001
	Senior high school	42.83±11.37		38.57±9.38	
	Junior college or undergraduate	41.71±11.67		37.92±9.24	
	Master or above	42.31±12.17		38.86±9.85	
Annual income	Less than 50000 CY	40.73±11.74	<0.001	36.49±8.86	<0.001
	50000-100000 CY	43.54±11.65		39.72±9.45	
	100000-200000 CY	42.46±11.79		39.17±9.67	
	200000-300000 CY	39.56±10.12		36.93±8.02	
	More than 300000 CY	42.19±12.56		40.05±11.42	
Health status	Poor	62.90±11.77	<0.001	55.94±12.3	<0.001
	Normal	49.73±12.34		44.56±10.55	
	Good	42.00±10.57		37.95±8.48	
	Very good	36.36±10.88		34.14±7.86	
Current occupation	Businessman	40.42±10.59	<0.001	37.29±8.37	<0.001
	Officer	40.25±9.25		37.86±7.63	
	Teacher	41.58±11.24		38.58±9.38	
	Police	38.89±11.34		36.71±8.89	
	Farmer	43.02±11.59		37.97±9.14	
	Employee	42.08±10.68		38.25±8.34	
	Doctor	43.85±12.39		40.12±10.32	
	Nurse	45.17±12.35		40.41±10.14	
	Medical technician	44.74±11.84		40.14±9.77	

	Retired re-employee	42.24±10.47		38.98±8.61	
	Non-medical student	38.43±10.73		34.5±7.32	
	Medical student	41.93±12.18		37.93±9.59	
Chronic disease	No chronic disease	40.27±11.12	<0.001	36.48±8.4	<0.05
	CDCPD	48.86±12.70		44.88±10.97	
	Chronic diseases only	45.27±11.42		41.47±9.45	

SD, standard deviation; CY, Chinese yuan; SGS-scale: the rhythm of sleep, getting-up, and socializing; EPE-scale: the rhythm of eating, physical practice, and entertainment activities; CDCPD, Chronic diseases comorbid with psychosomatic diseases.

Correlations between the social rhythm and psychological impacts

Among the 5854 participants, SGS-scale and EPE-scale were 20.65±9.01 and 8.12±6.00, respectively, and SAS score and SDS score were 30.51±7.49 and 41.95±11.75, respectively. Spearman correlation analysis found that SGS scale was moderately correlated with depression and anxiety, with the correlation coefficients of 0.550 and 0.544, respectively, (both *P*<0.001, S3 Table). In the multivariate linear regression analysis where the scores of SPS-scale or EPE-scale were taken as dependent variables, the score of SDS and SAS were taken as independent variables, and the age, gender, education, and occupation status were taken as confounding factors, the correlations between the scores of SPS-scale and EPE-scale and the scores of SDS and SAS remained unchanged (all *P*<0.05, Table 5, S1 Fig) .The disruption of sleep,

getting-up, and socializing rhythm was positively associated with depression and anxiety (*β*=0.355, *P*<0.001; β=0.186, *P*<0.001). The disruption of eating, physical practice, and entertainment rhythm showed opposite association to psychological impact: negatively related to depression and positively related to anxiety (*β*= -0.049, *P*=0.036; *β*= 0.082, *P*<0.001) (Table 5).

Table 5. Correlations between the social rhythm disruption and psychological impacts as determined by multivariate linear regressive analysis

			**Standardized coefficient (β)**	***t***	***P***	**EXP(B) 95% CI**	
						**Lower**	**Upper**
SGS-scale	Model 1	SDS	0.381	20.080	<0.001	0.264	0.321
		SAS	0.203	10.689	<0.001	0.160	0.231
	Model 2	SDS	0.373	19.463	<0.001	0.258	0.315
		SAS	0.200	10.385	<0.001	0.156	0.229
	Model 3	SDS	0.355	18.370	<0.001	0.243	0.302
		SAS	0.188	9.791	<0.001	0.145	0.217
	Model 4	SDS	0.355	18.373	<0.001	0.244	0.302
		SAS	0.186	9.645	<0.001	0.143	0.216
EPE-scale	Model 1	SDS	-0.082	-3.565	<0.001	-0.065	-0.019
		SAS	0.109	4.780	<0.001	0.041	0.099
	Model 2	SDS	-0.049	-2.129	0.033	-0.048	-0.002
		SAS	0.082	3.582	<0.001	0.024	0.082
	Model 3	SDS	-0.049	-2.101	0.036	-0.048	-0.002

	SAS	0.081	3.513	<0.001	0.023	0.081
Model 4	SDS	-0.049	-2.101	0.036	-0.048	-0.002
	SAS	0.082	3.518	<0.001	0.023	0.081

SGS-scale: the rhythm of sleep, getting-up, and socializing; EPE-scale: the rhythm of eating, physical practice, and entertainment activities; SDS/ SAS: Zung's self-rating depression and anxiety scales.

Model 1: uncorrected; Model 2: corrective factors: gender, age, marital status, and education level; Model 3: corrective factors: based on the corrective factors of Model 2 and health status, annual pre-tax income, and current occupation; and Model 4: corrective factors: based on the corrective factors of Model 3 and psychosomatic diseases. The results of Model 4 were presented in this table.

Discussion

The COVID-19 pandemic has brought great challenges and altered daily routines. It also undoubtedly contributes to emotional stress, fear, sadness, loneliness, anxiety and depression, and thus is associated with a global increase in psychological impacts[5]. One of the many mechanisms by which COVID-19-related changes in social rhythm impact mental health involves circadian disruption[7]. A previous study showed that the early identification and intervention of rhythm disruption are of important clinical

significance[21]. Rhythm disruption is part of the pathogenic cascade and is associated with mood disorders, such as anxiety, depression, low happiness, strong loneliness, and bipolar disorder during the non-outbreak period of infectious disease[7, 15, 21]. Limited studies have found that sleep disruption is related to depression in the general population during the COVID-19 pandemic[11]. The present study found that the disruption of social rhythm was quite different among people with various sociodemographic backgrounds. The female gender, low-degree education level, too-low or too-high income, poor health status, age group of 26-30 years and older than 61 years, nurses and subjects with divorce mostly suffered from disruption of social rhythm. In addition, the present study observed that the participants with female gender, age group of 26-40 years, divorce or widow status, low education level, too-low or too-high income, poor health status, and chronic diseases comorbid with psychosomatic diseases, and nurses mostly suffered from depression and/or anxiety.

There are two major possible explanations for the observed gender differences in rhythm disruption, depression, and anxiety. First, the steroid receptors are expressed in almost every site that receives direct suprachiasmatic nucleus (SCN) input[22]. The endogenous circadian clocks can be reset by hormone signals, affecting the balance of the hypothalamus-pituitary-adrenal (HPA) axis, and making the HPA axis more responsive to stress than males[22, 23]. The disruption of circadian rhythms within the HPA axis, and the sleep-arousal system differs between the genders, and is associated with dysfunction and disease[22]. Understanding gender differences in the circadian

timing system can lead to improved treatment strategies. Second, the psychosocial contexts differ between the genders, and the impact of traditional gender roles and socio-cultural influences on stress and anxiety are greater in women than in men[24].

In this study, we found that people with low education levels or low income mostly suffer from irregular social rhythms, likely due to their weak competitiveness in the job market and inability to adapt to the new E-commerce models during the COVID-19 pandemic due to the isolation or even lockdown. Moreover, they did not have sufficient judgment while facing various, overloaded and timeless information on the COVID-19 pandemic, and easily felt stressed, depressive and anxious[25]. Interestingly, in the present study, the people with high income were among those who mostly suffered from disrupted rhythms of entertainment during the COVID-19 pandemic. It has been shown that people with poor health status are likely to have basic diseases, weak immune function, and low ability to fight pathogens or adapt to a rhythm change[26], which may explain that people with poor health status suffered from disruption of social rhythm observed in the represent study. In addition, our finding that a high frequency of the irregular rhythm in the divorced group might be related to the disintegration of the family, loose family relations, economic problems, and the problem of raising children as a single parent. Especially, the isolation during the COVID-19 pandemic might have aggravated the fear for the unknown future, loneliness, helplessness, or irregular lifestyle[27].

The present study found that the people aged 26-40 years old and the elderly reported similarly high SGS-scale scores and were vulnerable to circadian disruption. The former is the main force of the society that promote the consumption, cultural creativity, and economic development[28]. However, when they must stay at home or fight against the COVID-19 pandemic due to the sudden outbreak of the disease, it is difficult for them to maintain a normal social rhythm. It is worth noting that the elderly reported the highest rate of rhythm disruption of eating, physical practice, and entertainment behaviors in the present study. Previously, Liu *et al.* [29]conducted a study on lifestyle and the health status of Chinee elderly, which included the elderly who lived in their own houses, with 20.6% of them being widows or widowers. They found that lack of physical practice, advanced age, and alcohol consumption were risk factors for their self-rate health status whereas a long sleep time was the protective factor[29]. Indeed, it has been reported that advanced age is characterized by a progressive decreasing amplitude and phase advance of circadian rhythmicity in overall biological functions, including blood pressure, hormonal circadian secretions, and immune function changes[30].

It is noteworthy to mention that nurses had the highest score of rhythm disruption in going to bed, get-up, and socializing among occupations, suggesting that they were more affected by the pandemic than other occupations such as businessmen, officers, teachers, police, farmers, doctors, and students. Night shift work has been thought to play a role through a disruption of the circadian rhythm, decreased synthesis of

melatonin and sleep deprivation[31]. We assume that nurses undertake a working articulation in shifts to keep the continuity of healthcare throughout a whole day, and the COVID-19 pandemic results in heavy workload, insufficient rest, quarantine, and social isolation. Consequently, the already dysfunctional social rhythm of nurses is further exacerbated. McElroy *et al*. reported that night and long shifts caused fatigue and adversely affected the family and social life of hospital employees[32]. Rosa *et al*. indicated that shift work could cause psychological consequences, including anxiety, stress, and depression, which is an obstacle for social and family relationships and a risk factor for metabolic disorders, diabetes, cardiovascular disorders, and breast cancer[33].

The present study firstly reported the critical connection between social rhythm disruption and psychological impacts in a large-scale population under the stress of such a pandemic. Our study further found that the scores of social rhythms were positively correlated with SDS and SAS scores under the stress of the COVID-19 pandemic. The following factors are speculated to be involved in the connection. The first factor is the social zeitgeber theory ~~that offers an explanation~~. The notion that life events increase the likelihood of mood episodes *via* decreased zeitgeber scaffolding for a vulnerable circadian system has been known as the social zeitgeber hypothesis, and the "social rhythm" refers to the cross-day stability of the timing of behaviors that act as zeitgebers[7]. Social stimuli under the stress of COVID-19 may affect the circadian behavioral programs by regulating the phase and period of circadian clocks (*i.e.* a

zeitgeber action, either direct or by conditioning to photic zeitgebers) which leads to various adverse mental health[34, 35]. In turn, depressive episodes that arise because of life events disturbing social zeitgebers subsequently affect biological rhythms, and thus derail social and biological rhythms [35-36]. This present study expanded the scope of application of these rhythm theories from patients with mood disorders to the population with different sociodemographic backgrounds. The second factor is the lockdown protocols that have been adopted to minimize COVID-19 pandemic transmission. The protocols may weaken the zeitgeber-setting mechanism, and impact the stability of the timing of daily behaviors, such as sleeping, getting up, socializing, eating, physical practice, and entertainment, which, in turn, lead to mental health disorders, such as anxiety and depression[7]. The third factor is SCN that serves as a master clock synchronizing the phase of clocks throughout the body. The disrupted rhythm in SCN leads to helplessness and behavioral despair, which increases depression and anxiety-related behaviors[36]. The fourth factor is the daily function of HPA activity, which is also regulated by the biological clock[37]. Anxiety disorders and major depressive disorder have been associated with increased and blunted HPA axis reactivity to social stress, which engages the diverse biological pathways that bolster successful stress adaptation and promote stress resilience. Adaptation to the challenges of stress is compromised when these pathways are no longer functioning optimally, resulting in the disrupted rhythms on psychological impacts[38]. Of course, other mechanisms may also connect social rhythm dysregulation with phycological impacts.

~~A previous study reported that the 4/4 genotype of the circadian gene, *PER3*, was associated with anxiety symptoms in 804 Brazilian non-clinical young adults[39]. Mathew and his colleagues also found that social jetlag (a misalignment between sleep timing on the weekend and school week) was positively and independently associated with anxiety symptoms in adolescents[40].~~

Based on the findings of the present study, more effective measures that combine circadian rhythms with the diagnosis and treatment of psychosomatic diseases may improve psychological impacts and maximize the effectiveness of treatment. Therefore, the following measures are recommended for stabilizing daily routines, especially for those specific population who mostly suffered from disrupted rhythms: 1), self-management strategies for setting up and increasing regularity of daily activities and lifestyle habits during the COVID-19 pandemic[7]; 2), spending 1 hour/day outdoors, walking for more than one hour a day and basking in the sun, which may regulate the body clock by the light-dark cycle; 3), scheduling social interactions at the same time of the day, and seeking the best candidates who may share thoughts and feelings in real time[7]; 4), eating meals at the same time every day, and adjusting the eating patterns and food categories. Previous studies have shown that regular eating and consumption of various metabolites promote the periodic colonization of the intestinal bacteria on the mucosal surface, and alleviate various adverse mental problems through the intestinal flora-gut-brain axis[41]; 5), avoiding daylight hour naps and the nocturnal blue spectrum light pollution from computers, mobile phones, electronic tablets and

televisions, which suppresses the sleep-helping hormones[7]. Chronotherapy seems to be a promising approach to restore the proper circadian pattern in the elderly with an obvious sleep problem through adequate sleep hygiene, timed light exposure, and the use of a chronobiotic medication[12]; 6), taking drugs that adjust the biological clock for nurses with sleep disorders caused by nurses' shift system when necessary[42]; and 7), encouraging online therapy program for normalizing the disrupted social rhythms in COVID-19 environment, with the support of health care professionals and persuasive systems design and user experience/interaction features[7].

The present study provides new ideas for the simultaneous intervention from the perspective of social rhythms and psychology, and would lay an epidemiological foundation for the interaction of social rhythms with the diagnosis and treatment of psychosomatic diseases and the maximization of therapeutic efficacy. However, there are a few limitations in the present study. First, this cross-sectional study cannot reveal causality, and the nature of voluntary participation may result in selection bias. Second, in this study, we did not track the 24-hour circadian rhythm. Indeed, it is necessary to track the circadian rhythms/sleep-wake cycle by a multi-lead-sleep monitor considering different psychological impacts, which will be implemented in our further research. Third, the survey response rate was not obtained since this online-survey was participated voluntarily and the lockdown and social isolation policy did not encourage a face-to-face survey, ~~and it was difficult to get the survey response rate~~. Fourth, we did not consider obtaining the individual informed consent for teenagers themselves under

18 years old, in addition to their parents' consent, which will also be implemented in our further similar research.

In conclusion, there exist rhythm disruptions among Chinese people with different sociodemographic backgrounds, which are closely associated with psychological impacts under the stress of the COVID-19 pandemic. Social rhythm disruption is independently associated with depression and anxiety. Interventions should be applied to the people vulnerable to the rhythm disruptions during the COVID-19 pandemic.

Acknowledgments

Zhihua Gao, PhD and Jinxia Zhu, PhD, provided valuable assistance with on questionnaire design. Kai Guo provided the on-line technical support. We are grateful for Medjaden Inc. for its assistance in the preparation of the manuscript and professional editing and language polishing.

Author Contributions

Min Yang, Ping He, Xiaoming Xu and Dan Li formulated the study design, carried out this study and wrote the manuscript. Jing Wang, Yanjun Wang and Mei Zhao collected

data and literature. Bin Wang and Wo Wang prepared the tables and format adjustment. Hui Lin and Ming-Ming Deng contributed to data analysis and data interpretation. Tianwei Deng, Li Kuang and Dongfeng Chen conceived the study and edited the manuscript.

References

1. Huang C, Wang Y, Li X, Ren L, Zhao J, Hu Y, et al. Clinical features of patients infected with 2019 novel coronavirus in Wuhan, China. Lancet (London, England). 2020;395(10223):497-506. Epub 2020/01/28. doi: 10.1016/s0140-6736(20)30183-5. PubMed PMID: 31986264; PubMed Central PMCID: PMCPMC7159299.

2. Li Q, Guan X, Wu P, Wang X, Zhou L, Tong Y, et al. Early Transmission Dynamics in Wuhan, China, of Novel Coronavirus-Infected Pneumonia. The New England journal of medicine. 2020;382(13):1199-207. Epub 2020/01/30. doi: 10.1056/NEJMoa2001316. PubMed PMID: 31995857; PubMed Central PMCID: PMCPMC7121484.

3. Zhou F, Yu T, Du R, Fan G, Liu Y, Liu Z, et al. Clinical course and risk factors for mortality of adult inpatients with COVID-19 in Wuhan, China: a retrospective cohort study. Lancet (London, England). 2020;395(10229):1054-62. Epub 2020/03/15. doi: 10.1016/s0140-6736(20)30566-3. PubMed PMID: 32171076; PubMed Central PMCID: PMCPMC7270627.

4. Bashir MF, Ma B, Shahzad L. A brief review of socio-economic and environmental impact of Covid-19. Air quality, atmosphere, & health. 2020:1-7. Epub 2020/08/25. doi: 10.1007/s11869-020-00894-8. PubMed PMID: 32837620; PubMed Central PMCID: PMCPMC7395801.

5. Pfefferbaum B, North CS. Mental Health and the Covid-19 Pandemic. The New

England journal of medicine. 2020;383(6):510-2. Epub 2020/04/14. doi: 10.1056/NEJMp2008017. PubMed PMID: 32283003.

6. Xiong J, Lipsitz O, Nasri F, Lui LMW, Gill H, Phan L, et al. Impact of COVID-19 pandemic on mental health in the general population: A systematic review. Journal of affective disorders. 2020;277:55-64. Epub 2020/08/18. doi: 10.1016/j.jad.2020.08.001. PubMed PMID: 32799105; PubMed Central PMCID: PMCPMC7413844.

7. Murray G, Gottlieb J, Swartz HA. Maintaining Daily Routines to Stabilize Mood: Theory, Data, and Potential Intervention for Circadian Consequences of COVID-19. Can J Psychiatry. 2020:706743720957825. Epub 2020/09/11. doi: 10.1177/0706743720957825. PubMed PMID: 32909832.

8. Crowe M, Inder M, Swartz HA, Murray G, Porter R. Social rhythm therapy-A potentially translatable psychosocial intervention for bipolar disorder. Bipolar disorders. 2020;22(2):121-7. Epub 2019/09/21. doi: 10.1111/bdi.12840. PubMed PMID: 31539451.

9. Haynes PL, Gengler D, Kelly M. Social Rhythm Therapies for Mood Disorders: an Update. Current psychiatry reports. 2016;18(8):75. Epub 2016/06/25. doi: 10.1007/s11920-016-0712-3. PubMed PMID: 27338753; PubMed Central PMCID: PMCPMC4919368.

10. Kim BN. Behavioral activation system (BAS) dysregulation and bipolar spectrum

psychopathology in daily life: An online-diary study. Psychiatry research. 2020;291:113180. Epub 2020/06/17. doi: 10.1016/j.psychres.2020.113180. PubMed PMID: 32544707.

11. Majumdar P, Biswas A, Sahu S. COVID-19 pandemic and lockdown: cause of sleep disruption, depression, somatic pain, and increased screen exposure of office workers and students of India. Chronobiology international. 2020;37(8):1191-200. Epub 2020/07/15. doi: 10.1080/07420528.2020.1786107. PubMed PMID: 32660352.

12. Cardinali DP, Brown GM, Reiter RJ, Pandi-Perumal SR. Elderly as a High-risk Group during COVID-19 Pandemic: Effect of Circadian Misalignment, Sleep Dysregulation and Melatonin Administration. Sleep and vigilance. 2020:1-7. Epub 2020/10/06. doi: 10.1007/s41782-020-00111-7. PubMed PMID: 33015537; PubMed Central PMCID: PMCPMC7519696.

13. Rodrigues Aguilar AC, Frange C, Huebra L, Dias Gomes AC, Tufik S, Santos Coelho FM. The effects of the COVID-19 pandemic on patients with narcolepsy. Journal of clinical sleep medicine : JCSM : official publication of the American Academy of Sleep Medicine. 2020. Epub 2020/10/31. doi: 10.5664/jcsm.8952. PubMed PMID: 33124977.

14. Türkoğlu S, Uçar HN, Çetin FH, Güler HA, Tezcan ME. The relationship between chronotype, sleep, and autism symptom severity in children with ASD in COVID-

19 home confinement period. Chronobiology international. 2020;37(8):1207-13. Epub 2020/08/05. doi: 10.1080/07420528.2020.1792485. PubMed PMID: 32746638.

15. Margraf J, Lavallee K, Zhang X, Schneider S. Social Rhythm and Mental Health: A Cross-Cultural Comparison. PLoS One. 2016;11(3):e0150312. Epub 2016/03/10. doi: 10.1371/journal.pone.0150312. PubMed PMID: 26954568; PubMed Central PMCID: PMCPMC4783111.

16. Gao ZH, Yang SQ, Margraf J, Zhang XC. Reliability and Validity for Rhythm Scale in Chinese. China journal of health psychology 2013;21(08):1219-21. (in Chinese) http://med.wanfangdata.com.cn/Paper/Detail/PeriodicalPaper_jkxlxzz201308042

17. Zung WW. A SELF-RATING DEPRESSION SCALE. Archives of general psychiatry. 1965;12:63-70. Epub 1965/01/01. doi: 10.1001/archpsyc.1965.01720310065008. PubMed PMID: 14221692.

18. Zung WW. A rating instrument for anxiety disorders. Psychosomatics. 1971;12(6):371-9. Epub 1971/11/01. doi: 10.1016/s0033-3182(71)71479-0. PubMed PMID: 5172928.

19. Peng S, Zhang YY, Ji Y, Tang WQ, Li Q, Yan XL, et al. Analysis of reliability and validity of Chinese version SDS Scale in women of rural area. Shanghai medical & pharmaceutical journal 2013;34(14):20-3. (in Chinese) http://med.wanfangdata.com.cn/Paper/Detail/PeriodicalPaper_shyy201314011

20. Hühne A, Welsh DK, Landgraf D. Prospects for circadian treatment of mood disorders. Annals of medicine. 2018;50(8):637-54. Epub 2018/09/29. doi: 10.1080/07853890.2018.1530449. PubMed PMID: 30265156.

21. Lyall LM, Wyse CA, Graham N, Ferguson A, Lyall DM, Cullen B, et al. Association of disrupted circadian rhythmicity with mood disorders, subjective wellbeing, and cognitive function: a cross-sectional study of 91 105 participants from the UK Biobank. The lancet Psychiatry. 2018;5(6):507-14. Epub 2018/05/20. doi: 10.1016/s2215-0366(18)30139-1. PubMed PMID: 29776774.

22. Bailey M, Silver R. Sex differences in circadian timing systems: implications for disease. Frontiers in neuroendocrinology. 2014;35(1):111-39. Epub 2013/11/30. doi: 10.1016/j.yfrne.2013.11.003. PubMed PMID: 24287074; PubMed Central PMCID: PMCPMC4041593.

23. Grafe LA, Bhatnagar S. The contribution of orexins to sex differences in the stress response. Brain Res. 2020;1731:145893. Epub 2018/08/07. doi: 10.1016/j.brainres.2018.07.026. PubMed PMID: 30081036; PubMed Central PMCID: PMCPMC6360123.

24. Schubert CC, Punamäki RL. Mental health among torture survivors: cultural background, refugee status and gender. Nordic journal of psychiatry. 2011;65(3):175-82. Epub 2010/09/22. doi: 10.3109/08039488.2010.514943. PubMed PMID: 20854222.

25. Pieh C, Budimir S, Probst T. The effect of age, gender, income, work, and physical activity on mental health during coronavirus disease (COVID-19) lockdown in Austria. Journal of psychosomatic research. 2020;136:110186. Epub 2020/07/19. doi: 10.1016/j.jpsychores.2020.110186. PubMed PMID: 32682159.

26. Koritala BSC, Çakmaklı S. The human circadian clock from health to economics. PsyCh journal. 2018;7(4):176-96. Epub 2018/11/21. doi: 10.1002/pchj.252. PubMed PMID: 30456783.

27. Groarke JM, Berry E, Graham-Wisener L, McKenna-Plumley PE, McGlinchey E, Armour C. Loneliness in the UK during the COVID-19 pandemic: Cross-sectional results from the COVID-19 Psychological Wellbeing Study. PLoS One. 2020;15(9):e0239698. Epub 2020/09/25. doi: 10.1371/journal.pone.0239698. PubMed PMID: 32970764; PubMed Central PMCID: PMCPMC7513993.

28. Nguyen C, Murray G, Anderson S, Filipowicz A, Ingram KK. In vivo molecular chronotyping, circadian misalignment, and high rates of depression in young adults. Journal of affective disorders. 2019;250:425-31. Epub 2019/03/18. doi: 10.1016/j.jad.2019.03.050. PubMed PMID: 30878655.

29. Liu WH, Zhou JY, Guo XY, Wu BY, Yu Q, Dong HL. Impact analysis of lifestyle on the health status of the elderly. Chinese primary health care 2018;32(10):29-31. (in Chinese) http://med.wanfangdata.com.cn/Paper/Detail/PeriodicalPaper_zgcjwsbj201810011

30. Lananna BV, Musiek ES. The wrinkling of time: Aging, inflammation, oxidative stress, and the circadian clock in neurodegeneration. Neurobiology of disease. 2020;139:104832. Epub 2020/03/18. doi: 10.1016/j.nbd.2020.104832. PubMed PMID: 32179175; PubMed Central PMCID: PMCPMC7727873.

31. Burdelak W, Peplońska B. [Night work and health of nurses and midviwes--a review]. Medycyna pracy. 2013;64(3):397-418. Epub 2013/11/23. PubMed PMID: 24261252.

32. McElroy SF, Olney A, Hunt C, Glennon C. Shift work and hospital employees: A descriptive multi-site study. International journal of nursing studies. 2020;112:103746. Epub 2020/09/16. doi: 10.1016/j.ijnurstu.2020.103746. PubMed PMID: 32928504.

33. Rosa D, Terzoni S, Dellafiore F, Destrebecq A. Systematic review of shift work and nurses' health. Occupational medicine (Oxford, England). 2019;69(4):237-43. Epub 2019/05/28. doi: 10.1093/occmed/kqz063. PubMed PMID: 31132107.

34. Grandin LD, Alloy LB, Abramson LY. The social zeitgeber theory, circadian rhythms, and mood disorders: review and evaluation. Clinical psychology review. 2006;26(6):679-94. Epub 2006/08/15. doi: 10.1016/j.cpr.2006.07.001. PubMed PMID: 16904251.

35. Healy D. Rhythm and blues. Neurochemical, neuropharmacological and neuropsychological implications of a hypothesis of circadian rhythm dysfunction in

the affective disorders. Psychopharmacology. 1987;93(3):271-85. Epub 1987/01/01. doi: 10.1007/bf00187243. PubMed PMID: 3124158.

36. Landgraf D, Long JE, Proulx CD, Barandas R, Malinow R, Welsh DK. Genetic Disruption of Circadian Rhythms in the Suprachiasmatic Nucleus Causes Helplessness, Behavioral Despair, and Anxiety-like Behavior in Mice. Biological psychiatry. 2016;80(11):827-35. Epub 2016/04/27. doi: 10.1016/j.biopsych.2016.03.1050. PubMed PMID: 27113500; PubMed Central PMCID: PMCPMC5102810.

37. Kinlein SA, Karatsoreos IN. The hypothalamic-pituitary-adrenal axis as a substrate for stress resilience: Interactions with the circadian clock. Frontiers in neuroendocrinology. 2020;56:100819. Epub 2019/12/22. doi: 10.1016/j.yfrne.2019.100819. PubMed PMID: 31863788; PubMed Central PMCID: PMCPMC7643247.

38. Fiksdal A, Hanlin L, Kuras Y, Gianferante D, Chen X, Thoma MV, et al. Associations between symptoms of depression and anxiety and cortisol responses to and recovery from acute stress. Psychoneuroendocrinology. 2019;102:44-52. Epub 2018/12/05. doi: 10.1016/j.psyneuen.2018.11.035. PubMed PMID: 30513499; PubMed Central PMCID: PMCPMC6420396.

39. Silva A, Dos Santos MJ, Góes Gitaí DL, de Miranda Coelho JAP, de Andrade TG. Depression and anxiety symptoms correlate with diurnal preference, sleep habits,

and Per3 VNTR polymorphism (rs57875989) in a non-clinical sample. Journal of affective disorders. 2020;277:260-70. Epub 2020/08/26. doi: 10.1016/j.jad.2020.07.138. PubMed PMID: 32841827.

40. Mathew GM, Li X, Hale L, Chang AM. Sleep duration and social jetlag are independently associated with anxious symptoms in adolescents. Chronobiology international. 2019;36(4):461-9. Epub 2019/02/23. doi: 10.1080/07420528.2018.1509079. PubMed PMID: 30786775; PubMed Central PMCID: PMCPMC6397070.

41. Kaczmarek JL, Thompson SV, Holscher HD. Complex interactions of circadian rhythms, eating behaviors, and the gastrointestinal microbiota and their potential impact on health. Nutrition reviews. 2017;75(9):673-82. Epub 2017/09/25. doi: 10.1093/nutrit/nux036. PubMed PMID: 28938796; PubMed Central PMCID: PMCPMC5914376.

42. Pevet P, Challet E. Melatonin: both master clock output and internal time-giver in the circadian clocks network. Journal of physiology, Paris. 2011;105(4-6):170-82. Epub 2011/09/15. doi: 10.1016/j.jphysparis.2011.07.001. PubMed PMID: 21914478.

Supporting Information

S1 Fig. Correlations of SGS-scale with SDS (A) and SAS (B)

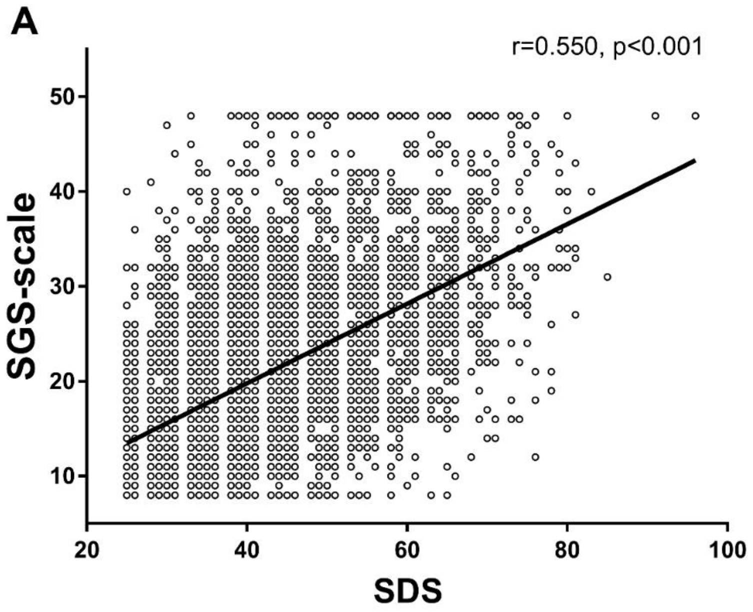

A

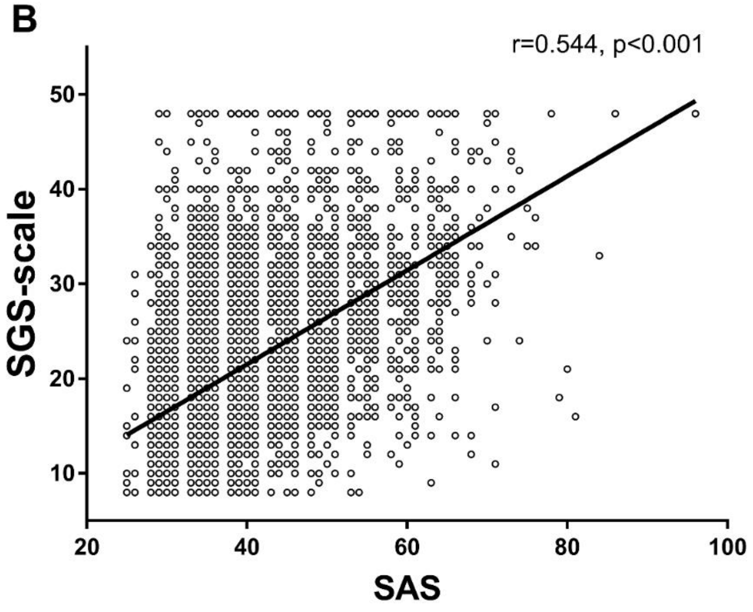

B

SGS-scale: the rhythm of sleep, getting-up, and socializing; SDS: Zung’s self-rate depression scale; SAS: Zung’s self-rate anxiety scale.

S1 Table. Prevalence of depression with different grades in participants (N=5854).

	N	Normal	Mild	Moderate	Severe	*P*
		N=4430	N=897	N=402	N=125	
**Health status**						
Poor	53	6(11.32%)	16(30.19%)	16(30.19%)	15(28.30%)	<0.001
Normal	787	410(52.10%)	204(25.92%)	121(15.37%)	52(6.61%)	
Good	3686	2849(77.29%)	576(15.63%)	218(5.91%)	43(1.17%)	
Very good	1328	1165(87.73%)	101(7.61%)	47(3.54%)	15(1.13%)	
**Current occupation**						
Businessman	481	388(80.67%)	64(13.31%)	25(5.20%)	4(0.83%)	<0.001
Officer	245	201(82.04%)	39(15.92%)	3(1.22%)	2(0.82%)	
Teacher	231	183(79.22%)	35(15.15%)	5(2.16%)	8(3.46%)	
Police	277	234(84.48%)	25(9.03%)	15(5.42%)	3(1.08%)	
Farmer	217	159(73.27%)	39(17.97%)	14(6.45%)	5(2.30%)	
Employee	270	208(77.04%)	47(17.41%)	12(4.44%)	3(1.11%)	
Doctor	1171	828(70.71%)	188(16.05%)	115(9.82%)	40(3.42%)	
Nurse	1017	682(67.06%)	186(18.29%)	113(11.11%)	36(3.54%)	
Medical technician	139	100(71.94%)	23(16.55%)	10(7.19%)	6(4.32%)	
Retired re-employee	229	174(75.98%)	42(18.34%)	9(3.93%)	4(1.75%)	
Non-medical student	1172	984(83.96%)	131(11.18%)	49(4.18%)	8(0.68%)	
Medical student	405	289(71.36%)	78(19.26%)	32(7.90%)	6(1.48%)	
**Chronic disease**						

No chronic disease	4373	3519(80.47%)	566(12.94%)	233(5.33%)	55(1.26%)	<0.001
CDCPD	671	363(54.10%)	171(25.48%)	90(13.41%)	47(7.00%)	
Chronic diseases only	810	548(67.65%)	160(19.75%)	79(9.75%)	23(2.84%)	

SDS, Zung's self-rating depression scale; CY, Chinese yuan; CDCPD, Chronic diseases comorbid with psychosomatic diseases.

S2 Table. Prevalence of anxiety with different grades in participants (N=5854).

	N	Normal	Mild	Moderate and severe	*P*
		N=5114	N=513	N=227	
**Health status**					
Poor	53	17(32.08%)	18(33.96%)	18(33.96%)	<0.001
Normal	787	550(69.89%)	161(20.46%)	76(9.66%)	
Good	3686	3297(89.45%)	287(7.79%)	102(2.77%)	
Very good	1328	1250(94.13%)	47(3.54%)	31(2.33%)	
**Current occupation**					
Businessman	481	438(91.06%)	31(6.44%)	12(2.49%)	<0.001
Officer	245	228(93.06%)	14(5.71%)	3(1.22%)	
Teacher	231	203(87.88%)	21(9.09%)	7(3.03%)	
Police	277	252(90.97%)	19(6.86%)	6(2.17%)	
Farmer	217	195(89.86%)	14(6.45%)	8(3.69%)	
Employee	270	240(88.89%)	25(9.26%)	5(1.85%)	
Doctor	1171	961(82.07%)	130(11.10%)	80(6.83%)	
Nurse	1017	824(81.02%)	129(12.68%)	64(6.29%)	
Medical technician	139	119(85.61%)	13(9.35%)	7(5.04%)	
Retired re-employee	229	200(87.34%)	23(10.04%)	6(2.62%)	
Non-medical student	1172	1105(94.28%)	53(4.52%)	14(1.19%)	
Medical student	405	349(86.17%)	41(10.12%)	15(3.70%)	
**Chronic disease**					

No chronic disease	4373	4004(91.56%)	262(5.99%)	107(2.45%)	<0.001
CDCPD	671	460(68.55%)	136(20.27%)	75(11.18%)	
Chronic diseases only	810	650(80.25%)	115(14.20%)	45(5.56%)	

SAS, Zung's self-rating anxiety scale; CY, Chinese yuan; CDCPD, Chronic diseases comorbid with psychosomatic diseases.

S3 Table. Spearman's correlation between subscale 1, subscale 2, Zung's self-rating depression scale (SDS)and Zung's self-rating anxiety scale (SAS). (N=5854)

Outcomes	Subscale1	Subscale2	SDS	SAS
**Subscale1**	-	0.058**	0.550**	0.544**
**Subscale2**	-	-	-0.037**	0.008
**SDS**	-	-	-	0.808**
**SAS**	-	-	-	-

**, *P*<0.001.

Dear Dr. Bullock,

Thank you for your decision letter and the reviewers for their time in reviewing our manuscript (ID: PONE-D-20-26146), entitled "**Disrupted rhythms of life, work and entertainment and their associations with psychological impacts under the stress of the COVID-19 pandemic: A Survey in 5854 Chinese people with different sociodemographic backgrounds**". According to your insightful advice and the thoughtful comments and suggestions of the reviewers, we have significantly revised our manuscript. All changes are highlighted in red in the revised manuscript. In addition, we have responded to the reviewers' comments in a point-by-point format, which are listed below this letter. We would like to re-submit it for your consideration. We hope that the revision is acceptable, and we look forward to hearing from you soon.

Response to the comments of Reviewer #1:

1. This is a timely and novel research project.

Overall language - While there is a very good flow of idea and structure to the manuscript, the language is awkward and redundant at times, and would benefit from additional editing by a fluent or native speaker of English.

Response: Thanks for your positive and encouraging comment on our research project. According to your suggestion, we have sent our manuscript to Medjaden Inc, a professional editing and proofreading service, for language polishing.

2. Statistical analysis - Please considering re-calculating probabilities while correcting for multiple comparisons/tests.

Response: Thanks for your professional suggestion. Accordingly, we have re-calculated probabilities while correcting for multiple comparisons. For more clarity and readability, we have split the original Table 1 into four tables, *i.e.* Table 1 (General sociodemographic characteristics of 5854 analyzed participants) (Page 9, Line 15 —

Page 10, Line 1); Table 2 (Scores of social rhythms in participants with different sociodemographic backgrounds during the COVID-19 pandemic (N=5854) (Page 11, Line 6 — Page 12, Line 3); Table 3 (Comparison of SGS-scale and EPE-scale scores between the group with the highest score and each of other groups in terms of sociodemographic backgrounds) (Page 12, Line 4 — Page 14, Line 3); Table 4 (Score of depression and anxiety in participants with different sociodemographic backgrounds during the COVID-19 pandemic, N=5854) (Page 15, Line 4 — Page 16, Line 3). The overall χ^2, Z and P-values in the multiple comparisons are shown in Table 2. Additionally, we have conducted comparisons between two groups for each variable and presented the results for the comparisons between the group with the highest score and each of other groups in SGS-scale (Table 3) (Page 12, Line 4 — Page 14, Line 3).

3. Please consider reducing the table content to improve readability.

Response: As described above, we have split the original Table 1 into four tables (Tables 1-4) for clarity, and deleted the columns of SAS and SDS in Table 1 in the revised manuscript. We have labeled the original Tables 2, 3 and 4 as Supplementary Tables 1 and 2, and 3 in the revised manuscript (Page 38-40). In addition, we have combined the original Tables 5 and 6 into a single table (Table 5) in the revised manuscript (Page 17, Line 10—Page 18, Line 3). Thus, the formal table number has been reduced from 6 to 5, with three complementary tables in the revised manuscript. In addition, we have labeled the original Figures 1 and 2 as Supplementary Figures 1A &B in the revised manuscript (Page 37).

Responses to the comments of Reviewer #2:

1. Is the manuscript technically sound, and do the data support the conclusions? See comments on the discussion

Response: Thanks for your positive and valuable comments. Our responses are listed below your comments on the Discussion accordingly. Moreover, we have significantly revised the Discussion section of the revised manuscript (Page 19, Lines 11-20; Page 21, Lines 6-21; Page 22, Lines 1- 21; Page 23, Lines 1-21 and Page 24, Lines 1-4).

2. Has the statistical analysis been performed appropriately and rigorously? No revisions

Response: Thanks for your professional suggestion. Accordingly, we have re-calculated probabilities while correcting for multiple comparisons. For more clarity and readability, we have split the original Table 1 into four tables, *i.e*. Table 1 (General sociodemographic characteristics of 5854 analyzed participants) (Page 9, Line 15 — Page 10, Line 1); Table 2 (Scores of social rhythms in participants with different sociodemographic backgrounds during the COVID-19 pandemic (N=5854) (Page 11, Line 6 — Page 12, Line 3); Table 3 (Comparison of SGS-scale and EPE-scale scores between the group with the highest score and each of other groups in terms of sociodemographic backgrounds) (Page 12, Line 4 — Page 14, Line 3); Table 4 (Score of depression and anxiety in participants with different sociodemographic backgrounds during the COVID-19 pandemic, N=5854) (Page 15, Line 4 — Page 16, Line 3). The overall χ^2, Z and P-values in the multiple comparisons are shown in Table 2. Additionally, we have conducted comparisons between two groups for each variable and presented the results for the comparisons between the group with the highest score and each of other groups in SGS-scale (Table 3) (Page 12, Line 4 — Page 14, Line 3).

3. Have the authors made all data underlying the findings in their manuscript fully available? No revisions.

Response: Thanks for your professional suggestion. We have made all data underlying the findings in the revised manuscript fully available. According to your insightful advice, these data have been reflected in the revised version of the Results, Discussions, and Supplementary materials.

4. Is the manuscript written in an intelligible fashion and written in standard English? While I answered yes, I think this manuscript calls for improvement and revisions in this category before publication. For example in the abstract the background and objective are awkward and hard to follow. As is, they read: “The rhythms of life, work and entertainment behaviors are considered as the external behavioral manifestations of biological rhythm. The present study aimed to evaluate the distinctive disrupted rhythms of behaviors and their associations with mental health problems in people with different backgrounds under the stress of COVID19 pandemic.” Social rhythms such as work and entertainment routines are driven by biological rhythms including the sleep wake cycle. The present study aimed to evaluate if disruption of social rhythms and routines were associated with mental health problems under the stress of the COVID19 pandemic and if this differed by people of XXX.”

Response: Thanks for your good suggestion. As for manuscript writing and language, we have tried our best to revise and improve the manuscript. In addition, we have sent our revised manuscript to Medjaden Inc, a professional editing and proofreading service, for language polishing.

Additional comments:

Introduction:

1. The Introduction needs to be expanded and to include more of the relevant literature. You seem to be missing a large body of research on this topic. Please see the article by Murray, Gottleib, & Swartz (2020) Maintaining Daily Routines to Stabilize Mood: Theory, Data, and Potential Intervention for Circadian Consequences of COVID-19. Canadian Journal of Psychiatry. If you look at the reference list of this paper, you will find more literature on this topic not included in your Background section.

Response: Thanks for your professional suggestion, and providing the valuable article, which we have learned and cited in the Introduction and Discussion sections of the

revised manuscript (Page 5, Lines 13-14; Page 22, Lines 14-21; Page 23, Lines 1-9; Page 23, Lines 9-21 and Page 24, Lines 1-4). Accordingly, we have made revisions with more literature on this topic in the Introduction section of the revised manuscript (Reference 5, 7, 23, and 24).

2. Specify what groups you are comparing in your aims.

Response: Thanks for your professional suggestion. The aim of the present study was to evaluate the social rhythm and its associations with the psychological impacts during the initial phase of the COVID-19 pandemic among Chinese people with different sociodemographic backgrounds including gender, age, marital status, education level, annual income, health status, current occupation, and chronic disease status. Moreover, we also compared the group with the highest SGS-scale and EPE-scale scores with each of other groups in terms of the above-mentioned variables (Table 3) (Page 12, Line 4 — Page 14, Line 3). Special groups referred to nurses, the elderly and subjects being divorced or with chronic diseases or psychosomatic diseases who were mostly suffered from disrupted rhythms of life-work behaviors. This point has been clarified in the revised manuscript (Page 10, Lines 7-14; Page 11, Lines 1-5).

3. Is this exploratory or do you have hypotheses?

Response: Yes, we have a hypothesis. We hypothesize that people with different sociodemographic backgrounds have different degrees of rhythm disruption in life, work, and entertainment, that the rhythm disruption is more severe in some specific groups, and that the rhythm disruption is associated with psychological impacts during the initial phase of the COVID-19 pandemic. Accordingly, we have stated the hypotheses clearly in the Introduction of the revised manuscript (Page 6, Lines 4-8).

Methods:

1. In the limitations I would include that those under 18, at least those over 13 could have signed/checked their own assent in addition to their parents' consent.

Response: Thanks for your thoughtful suggestion. Indeed, we did not consider obtaining the individual informed consent for teenagers themselves, in addition to their parents' consent. According to your suggestion, we have added it as a limitation in the Discussion section of the revised manuscript (Page 25, Line 21 and Page 26 Lines 1-2).

2. I suggest giving brief information on the psychometric properties for your instruments.

Response: Thanks for your professional suggestion. Accordingly, we have briefly described the psychometric properties for the instruments used in our study with references to the initial version and the Chinese version of SAS and SDS to interpret their reliability and validity-in the revised manuscript (Zung's Depression and Anxiety Scale, Page 8, Lines 11-14).

3. Did you track ask about circadian rhythms/sleep wake cycle? If not, this is a limitation.

Response: Thanks for your thoughtful suggestion. In this study, we did not track the 24-hour circadian rhythm. Indeed, it is necessary to track the circadian rhythms/sleep wake cycle by multilead-sleep monitor considering different psychological impacts, which will be implemented in our further research. According to your suggestion, we have added this point as a limitation in the Discussion section of the revised manuscript (Page 25, Lines 15-18).

Statistical Analysis:

Specify what groups you are comparing. If a one-way t-test was used specify your hypothesis after your aims.

Response: Thanks for your professional suggestion. In the present study, SGS-scale and EPE-scale scores reflecting the life-work and entertainment activities, respectively were compared among Chinese people with different sociodemographic backgrounds including gender, age, marital status, education level, annual income, health status, current occupation, and chronic disease status. Moreover, the group with the highest score was compared with each of other groups in terms of above-mentioned variables by using one-way ANOVA with a post hoc test (Table 3) (Page 12, Line 4 — Page 14, Line 3). This point has been clearly stated in the Statistical analysis of the revised manuscript (Page 8, Lines 18-19). In addition, we have specified our hypothesis after the aim (Page 6, Lines 4-8).

Results:

1. What was the response rate?

Response: Thanks for your pertinent question. It was challenging to get the survey response rate because the lockdown and social isolation policy did not encourage a face-to-face survey. Therefore, we conducted the present study using online-survey through a public media (*i.e.* EQxiu online questionnaire platform), to which participation was voluntary. Accordingly, we have added this point as a limitation in the Discussion section of the revised manuscript (Page 25, Lines 18-21).

2. Give an example of why some questionnaires were invalid and therefore excluded.

Response: Eighteen questionnaires with invalid were excluded the data analysis due to frivolous or incomplete answers, or the same option for different questions or

orderly answers. The information on the invalid questionnaires have been clearly presented in the Results section of the revised manuscript (Page 9, Lines 10-12).

3. In section 4 (Correlation analysis) specify what you mean by low score and high score group the first time you say it. Also, specify what the subscales are instead of referring to them as subscale1, etc.

Response: Thanks for carefully reviewing the manuscript and raising the suggestions. We have deleted the classification of “low score and high score groups”. For more readability, we have renamed the two subscales of the social rhythm scale (*i.e.* subscale1 and subscale2) as SGS-scale and EPE-scale, respectively, in the revised manuscript. Specifically, the first eight items constituted SGS-scale, which addresses sleeping, getting up, and socializing rhythm, and the last nine items constituted the EPE-scale, which addresses eating, physical practice, and entertainment rhythm. These terms have been clearly defined in the Measures section of the revised manuscript (Page 7, Lines 16-20).

Discussion:

There seems to be a lot of speculation in this section and assumptions that is not backed up by research, the literature or the data. You may want to back up your paper/assertions with theory. See: Social Rhythms Disruption Theory.

Response: Thanks for your critical and constructive comment and suggestion. Accordingly, we have performed literature search and learned several theories on social rhythm, such as Social Zeitgeber Theory. Accordingly, we have extensively revised the Discussion section of the revised manuscript (Page 22, Lines 13-21; Page 23, Lines 1-21; Page 24, Lines 1-4).

There are five parts (paragraphs) in the Discussion section of the revised manuscript, including: 1), the characteristic analysis of disrupted rhythms with different

sociodemographic backgrounds under the stress of COVID-19 pandemic, especially susceptible population suffering from rhythm disruption; 2), the correlations between rhythm disruption and psychological impacts, with explanations of disrupted rhythms on psychological impacts; 3), strategic measurement to restore the rhythm pattern; and 4), limitation; and 5), conclusion.

Responses to Additional Editor Comments:

1.Thank you for your interesting and timely research manuscript. The topic is clearly of current interest, however major revisions are necessary in order for this manuscript to be publishable. Reviewer 2 in particular provides comprehensive feedback on areas of the manuscript requiring significant revision.

Response: We have revised this manuscript according to the two reviewers' comments, with point-by-point responses.

2. Both reviewers recommend that the manuscript would benefit from thorough editing of language usage, spelling, and grammar by a fluent or native speaker of English. If you do not know anyone who can help you do this, you may wish to consider employing a professional scientific editing service.

Whilst you may use any professional scientific editing service of your choice, PLOS has partnered with both American Journal Experts (AJE) and Editage to provide discounted services to PLOS authors. Both organizations have experience helping authors meet PLOS guidelines and can provide language editing, translation, manuscript formatting, and figure formatting to ensure your manuscript meets our submission guidelines. To take advantage of our partnership with AJE, visit the AJE website (http://learn.aje.com/plos/) for a 15% discount off AJE services. To take advantage of our partnership with Editage, visit the Editage website (www.editage.com) and enter referral code PLOSEDIT for a 15% discount off Editage services. If the PLOS

editorial team finds any language issues in text that either AJE or Editage has edited, the service provider will re-edit the text for free.

Response: Thank you for the advice. We have tried our best to improve the manuscript. In addition, we have sent our revised manuscript to Medjaden Inc, a professional editing and proofreading service, for language polishing.

3.In addition to the feedback provided by the reviewers I suggest that any revised submission particularly focus on improving the Discussion. A more sophisticated discussion around HPA axis activity, SCN regulation of circadian rhythms, and social rhythms is necessary (see also Reviewer 2's feedback on the Discussion). For example, the relevance of the "knockout monkey" model is unclear. I also suggest a more sophisticated discussion of the possible explanations for the observed gender differences in stress and anxiety. The manuscript relies on evidence from rat studies and, unusually, transgender individuals, to support increased HPA axis activity in females. A more nuanced discussion is needed that may or may not include reference to these biological differences between men and women, but that also considers differences in psychosocial contexts between the sexes and the impact of traditional gender roles and socio-cultural influences on higher rates of stress and anxiety among women in comparison to men. There is also an unusual reference to the impact of menopause on "women's circadian and endocrine disorders" that is not supported by a reference, nor is it a measured variable in the study.

Response: Thanks for your insightful and constructive advice. We have performed literature search, read relevant articles and revised Discussion section of the revised manuscript according to your professional suggestion and the advice of reviewer 2 (Page 19, Lines 11-20; Page 20, Lines 1-2; Page 21, Lines 6-21; Page 22, Lines 1-21; Page 23, Lines 1-21; Page 24, Lines 1-4 ; Page 25, Lines 5-8 and Lines 15-21; Page 26, lines 1-2.
