# Supplementary material for: Disrupted rhythms of life, work and entertainment and their associations with psychological impacts under the stress of the COVID-19 pandemic: A survey in 5854 Chinese people with different sociodemographic backgrounds
Source: PLoS One. 2021 May 17;16(5):e0250770. doi: 10.1371/journal.pone.0250770 (PMC8128272; doi:10.1371/journal.pone.0250770)
Supplement: Attachment — Submitted filename: Informed consent.doc [file pone.0250770.s007.doc]

**Informed consent**

The purpose of this survey is to investigate your work, life, entertainment behavior and mental health under the pressure of the current epidemic of new coronary pneumonia, and the results are used only for statistical purposes and strictly confidential. Please take a few minutes to fill out this questionnaire. This questionnaire is anonymous, all data is only used for statistical analysis, please feel free to fill in. We hope to get your real ideas and valuable advice. Thank you for your cooperation!
