# Supplementary material for: Disrupted rhythms of life, work and entertainment and their associations with psychological impacts under the stress of the COVID-19 pandemic: A survey in 5854 Chinese people with different sociodemographic backgrounds
Source: PLoS One. 2021 May 17;16(5):e0250770. doi: 10.1371/journal.pone.0250770 (PMC8128272; doi:10.1371/journal.pone.0250770)
Supplement: Attachment — Submitted filename: Response to reviewers, 1-25.docx [file pone.0250770.s008.docx]

**Dear Dr. Bullock,**

Thank you for your decision letter and the reviewers for their time in reviewing our manuscript ( ID: PONE-D-20-26146), entitled “**Disrupted rhythms of life, work and entertainment and their associations with psychological impacts under the stress of the COVID-19 pandemic: A Survey in 5854 Chinese people with different sociodemographic backgrounds**”. According to your insightful advice and the thoughtful comments and suggestions of the reviewers, we have significantly revised our manuscript. All changes are highlighted in red in the revised manuscript. In addition, we have responded to the reviewers’ comments in a point-by-point format, which are listed below this letter. We would like to re-submit it for your consideration. We hope that the revision is acceptable, and we look forward to hearing from you soon.

**Response to the comments of Reviewer #1:**

1. This is a timely and novel research project.
Overall language - While there is a very good flow of idea and structure to the manuscript, the language is awkward and redundant at times, and would benefit from additional editing by a fluent or native speaker of English.
Response: Thanks for your positive and encouraging comment on our research project. According to your suggestion, we have sent our manuscript to Medjaden Inc, a professional editing and proofreading service, for language polishing.

2. Statistical analysis - Please considering re-calculating probabilities while correcting for multiple comparisons/tests.

Response: Thanks for your professional suggestion. Accordingly, we have re-calculated probabilities while correcting for multiple comparisons. For more clarity and readability, we have split the original Table 1 into four tables, *i.e*. Table 1 (General sociodemographic characteristics of 5854 analyzed participants) (Page 9, Line 15 — Page 10, Line 1); Table 2 (Scores of social rhythms in participants with different sociodemographic backgrounds during the COVID-19 pandemic (N=5854) (Page 11, Line 6 — Page 12, Line 3); Table 3 (Comparison of SGS-scale and EPE-scale scores between the group with the highest score and each of other groups in terms of sociodemographic backgrounds) (Page 12, Line 4 — Page 14, Line 3); Table 4 (Score of depression and anxiety in participants with different sociodemographic backgrounds during the COVID-19 pandemic, N=5854) (Page 15, Line 4 — Page 16, Line 3). The overall χ^2^, Z and P-values in the multiple comparisons are shown in Table 2. Additionally, we have conducted comparisons between two groups for each variable and presented the results for the comparisons between the group with the highest score and each of other groups in SGS-scale (Table 3) (Page 12, Line 4 — Page 14, Line 3).

3. Please consider reducing the table content to improve readability.

Response: As described above, we have split the original Table 1 into four tables (Tables 1-4) for clarity, and deleted the columns of SAS and SDS in Table 1 in the revised manuscript. We have labeled the original Tables 2, 3 and 4 as Supplementary Tables 1 and 2, and 3 in the revised manuscript (Page 38-40). In addition, we have combined the original Tables 5 and 6 into a single table (Table 5) in the revised manuscript (Page 17, Line 10—Page 18, Line 3). Thus, the formal table number has been reduced from 6 to 5, with three complementary tables in the revised manuscript. In addition, we have labeled the original Figures 1 and 2 as Supplementary Figures 1A &B in the revised manuscript (Page 37).

**Responses to the comments of Reviewer #2:**

1. Is the manuscript technically sound, and do the data support the conclusions? See comments on the discussion

Response: Thanks for your positive and valuable comments. Our responses are listed below your comments on the Discussion accordingly. Moreover, we have significantly revised the Discussion section of the revised manuscript (Page 19, Lines 11-20; Page 21, Lines 6-21; Page 22, Lines 1- 21; Page 23, Lines 1-21 and Page 24, Lines 1-4).

2. Has the statistical analysis been performed appropriately and rigorously? No revisions

Response: Thanks for your professional suggestion. Accordingly, we have re-calculated probabilities while correcting for multiple comparisons. For more clarity and readability, we have split the original Table 1 into four tables, *i.e*. Table 1 (General sociodemographic characteristics of 5854 analyzed participants) (Page 9, Line 15 — Page 10, Line 1); Table 2 (Scores of social rhythms in participants with different sociodemographic backgrounds during the COVID-19 pandemic (N=5854) (Page 11, Line 6 — Page 12, Line 3); Table 3 (Comparison of SGS-scale and EPE-scale scores between the group with the highest score and each of other groups in terms of sociodemographic backgrounds) (Page 12, Line 4 — Page 14, Line 3); Table 4 (Score of depression and anxiety in participants with different sociodemographic backgrounds during the COVID-19 pandemic, N=5854) (Page 15, Line 4 — Page 16, Line 3). The overall χ^2^, Z and P-values in the multiple comparisons are shown in Table 2. Additionally, we have conducted comparisons between two groups for each variable and presented the results for the comparisons between the group with the highest score and each of other groups in SGS-scale (Table 3) (Page 12, Line 4 — Page 14, Line 3).

3. Have the authors made all data underlying the findings in their manuscript fully available? No revisions.

Response: Thanks for your professional suggestion. We have made all data underlying the findings in the revised manuscript fully available. According to your insightful advice, these data have been reflected in the revised version of the Results, Discussions, and Supplementary materials.

4. Is the manuscript written in an intelligible fashion and written in standard English?
While I answered yes, I think this manuscript calls for improvement and revisions in this category before publication. For example in the abstract the background and objective are awkward and hard to follow. As is, they read: “The rhythms of life, work and entertainment behaviors are considered as the external behavioral manifestations of biological rhythm. The present study aimed to evaluate the distinctive disrupted rhythms of behaviors and their associations with mental health problems in people with different backgrounds under the stress of COVID19 pandemic.” Social rhythms such as work and entertainment routines are driven by biological rhythms including the sleep wake cycle. The present study aimed to evaluate if disruption of social rhythms and routines were associated with mental health problems under the stress of the COVID19 pandemic and if this differed by people of XXX.”

Response: Thanks for your good suggestion. As for manuscript writing and language, we have tried our best to revise and improve the manuscript. In addition, we have sent our revised manuscript to Medjaden Inc, a professional editing and proofreading service, for language polishing.

Additional comments:
Introduction:
1. The Introduction needs to be expanded and to include more of the relevant literature. You seem to be missing a large body of research on this topic. Please see the article by Murray, Gottleib, & Swartz (2020) Maintaining Daily Routines to Stabilize Mood: Theory, Data, and Potential Intervention for Circadian Consequences of COVID-19. Canadian Journal of Psychiatry. If you look at the reference list of this paper, you will find more literature on this topic not included in your Background section.

Response: Thanks for your professional suggestion, and providing the valuable article, which we have learned and cited in the Introduction and Discussion sections of the revised manuscript (Page 5, Lines 13-14; Page 22, Lines 14-21; Page 23, Lines 1-9; Page 23, Lines 9-21 and Page 24, Lines 1-4). Accordingly, we have made revisions with more literature on this topic in the Introduction section of the revised manuscript (Reference 5, 7, 23, and 24 ).

2. Specify what groups you are comparing in your aims.

Response: Thanks for your professional suggestion. The aim of the present study was to evaluate the social rhythm and its associations with the psychological impacts during the initial phase of the COVID-19 pandemic among Chinese people with different sociodemographic backgrounds including gender, age, marital status, education level, annual income, health status, current occupation, and chronic disease status. Moreover, we also compared the group with the highest SGS-scale and EPE-scale scores with each of other groups in terms of the above-mentioned variables (Table 3) (Page 12, Line 4 — Page 14, Line 3). Special groups referred to nurses, the elderly and subjects being divorced or with chronic diseases or psychosomatic diseases who were mostly suffered from disrupted rhythms of life-work behaviors. This point has been clarified in the revised manuscript (Page 10, Lines 7-14; Page 11, Lines 1-5).

3. Is this exploratory or do you have hypotheses?

Response: Yes, we have a hypothesis. We hypothesize that people with different sociodemographic backgrounds have different degrees of rhythm disruption in life, work, and entertainment, that the rhythm disruption is more severe in some specific groups, and that the rhythm disruption is associated with psychological impacts during the initial phase of the COVID-19 pandemic. Accordingly, we have stated the hypotheses clearly in the Introduction of the revised manuscript (Page 6, Lines 4-8).

Methods:

1. In the limitations I would include that those under 18, at least those over 13 could have signed/checked their own assent in addition to their parents’ consent.

Response: Thanks for your thoughtful suggestion. Indeed, we did not consider obtaining the individual informed consent for teenagers themselves, in addition to their parents’ consent. According to your suggestion, we have added it as a limitation in the Discussion section of the revised manuscript (Page 25, Line 21 and Page 26 Lines 1-2).

2. I suggest giving brief information on the psychometric properties for your instruments.

Response: Thanks for your professional suggestion. Accordingly, we have briefly described the psychometric properties for the instruments used in our study with references to the initial version and the Chinese version of SAS and SDS to interpret their reliability and validity in the revised manuscript (Zung’s Depression and Anxiety Scale, Page 8, Lines 11-14).

3. Did you track ask about circadian rhythms/sleep wake cycle? If not, this is a limitation.

Response: Thanks for your thoughtful suggestion. In this study, we did not track the 24-hour circadian rhythm. Indeed, it is necessary to track the circadian rhythms/sleep wake cycle by multilead-sleep monitor considering different psychological impacts, which will be implemented in our further research. According to your suggestion, we have added this point as a limitation in the Discussion section of the revised manuscript (Page 25, Lines 15-18).

Statistical Analysis:
Specify what groups you are comparing. If a one-way t-test was used specify your hypothesis after your aims.

Response: Thanks for your professional suggestion. In the present study, SGS-scale and EPE-scale scores reflecting the life-work and entertainment activities, respectively were compared among Chinese people with different sociodemographic backgrounds including gender, age, marital status, education level, annual income, health status, current occupation, and chronic disease status. Moreover, the group with the highest score was compared with each of other groups in terms of above-mentioned variables by using one-way ANOVA with a post hoc test (Table 3) (Page 12, Line 4 — Page 14, Line 3). This point has been clearly stated in the Statistical analysis of the revised manuscript (Page 8, Lines 18-19). In addition, we have specified our hypothesis after the aim (Page 6, Lines 4-8).

Results:
1. What was the response rate?

Response: Thanks for your pertinent question. It was challenging to get the survey response rate because the lockdown and social isolation policy did not encourage a face-to-face survey. Therefore, we conducted the present study using online-survey through a public media (*i.e.* EQxiu online questionnaire platform), to which participation was voluntary. Accordingly, we have added this point as a limitation in the Discussion section of the revised manuscript (Page 25, Lines 18-21).

2. Give an example of why some questionnaires were invalid and therefore excluded.

Response: Eighteen questionnaires with invalid were excluded the data analysis due to frivolous or incomplete answers, or the same option for different questions or orderly answers. The information on the invalid questionnaires have been clearly presented in the Results section of the revised manuscript (Page 9, Lines 10-12).

3. In section 4 (Correlation analysis) specify what you mean by low score and high score group the first time you say it. Also, specify what the subscales are instead of referring to them as subscale1, etc.

Response: Thanks for carefully reviewing the manuscript and raising the suggestions. We have deleted the classification of “low score and high score groups”. For more readability, we have renamed the two subscales of the social rhythm scale (*i.e.* subscale1 and subscale2) as SGS-scale and EPE-scale, respectively, in the revised manuscript. Specifically, the first eight items constituted SGS-scale, which addresses sleeping, getting up, and socializing rhythm, and the last nine items constituted the EPE-scale, which addresses eating, physical practice, and entertainment rhythm. These terms have been clearly defined in the Measures section of the revised manuscript (Page 7, Lines 16-20).

Discussion:
There seems to be a lot of speculation in this section and assumptions that is not backed up by research, the literature or the data. You may want to back up your paper/assertions with theory. See: Social Rhythms Disruption Theory.

Response: Thanks for your critical and constructive comment and suggestion. Accordingly, we have performed literature search and learned several theories on social rhythm, such as Social Zeitgeber Theory. Accordingly, we have extensively revised the Discussion section of the revised manuscript (Page 22, Lines 13-21; Page 23, Lines 1-21; Page 24, Lines 1-4).

There are five parts (paragraphs) in the Discussion section of the revised manuscript, including: 1), the characteristic analysis of disrupted rhythms with different sociodemographic backgrounds under the stress of COVID-19 pandemic, especially susceptible population suffering from rhythm disruption; 2), the correlations between rhythm disruption and psychological impacts, with explanations of disrupted rhythms on psychological impacts; 3), strategic measurement to restore the rhythm pattern; and 4), limitation; and 5), conclusion.

**Responses to Additional Editor Comments:**

1.Thank you for your interesting and timely research manuscript. The topic is clearly of current interest, however major revisions are necessary in order for this manuscript to be publishable. Reviewer 2 in particular provides comprehensive feedback on areas of the manuscript requiring significant revision.

Response: We have revised this manuscript according to the two reviewers’ comments, with point-by-point responses.

2. Both reviewers recommend that the manuscript would benefit from thorough editing of language usage, spelling, and grammar by a fluent or native speaker of English. If you do not know anyone who can help you do this, you may wish to consider employing a professional scientific editing service.

Whilst you may use any professional scientific editing service of your choice, PLOS has partnered with both American Journal Experts (AJE) and Editage to provide discounted services to PLOS authors. Both organizations have experience helping authors meet PLOS guidelines and can provide language editing, translation, manuscript formatting, and figure formatting to ensure your manuscript meets our submission guidelines. To take advantage of our partnership with AJE, visit the AJE website (http://learn.aje.com/plos/) for a 15% discount off AJE services. To take advantage of our partnership with Editage, visit the Editage website (www.editage.com) and enter referral code PLOSEDIT for a 15% discount off Editage services. If the PLOS editorial team finds any language issues in text that either AJE or Editage has edited, the service provider will re-edit the text for free.

Response: Thank you for the advice. We have tried our best to improve the manuscript. In addition, we have sent our revised manuscript to Medjaden Inc, a professional editing and proofreading service, for language polishing.

3.In addition to the feedback provided by the reviewers I suggest that any revised submission particularly focus on improving the Discussion. A more sophisticated discussion around HPA axis activity, SCN regulation of circadian rhythms, and social rhythms is necessary (see also Reviewer 2's feedback on the Discussion). For example, the relevance of the "knockout monkey" model is unclear. I also suggest a more sophisticated discussion of the possible explanations for the observed gender differences in stress and anxiety. The manuscript relies on evidence from rat studies and, unusually, transgender individuals, to support increased HPA axis activity in females. A more nuanced discussion is needed that may or may not include reference to these biological differences between men and women, but that also considers differences in psychosocial contexts between the sexes and the impact of traditional gender roles and socio-cultural influences on higher rates of stress and anxiety among women in comparison to men. There is also an unusual reference to the impact of menopause on "women's circadian and endocrine disorders" that is not supported by a reference, nor is it a measured variable in the study.

Response: Thanks for your insightful and constructive advice. We have performed literature search, read relevant articles and revised Discussion section of the revised manuscript according to your professional suggestion and the advice of reviewer 2 (Page 19, Lines 11-20; Page 20, Lines 1-2; Page 21, Lines 6-21; Page 22, Lines 1-21; Page 23, Lines 1-21; Page 24, Lines 1-4 ; Page 25, Lines 5-8 and Lines 15-21; Page 26, lines 1-2.
