# Supplementary material for: Disrupted rhythms of life, work and entertainment and their associations with psychological impacts under the stress of the COVID-19 pandemic: A survey in 5854 Chinese people with different sociodemographic backgrounds
Source: PLoS One. 2021 May 17;16(5):e0250770. doi: 10.1371/journal.pone.0250770 (PMC8128272; doi:10.1371/journal.pone.0250770)
Supplement: Attachment — Submitted filename: Response to Reviewers.docx [file pone.0250770.s010.docx]

Dear Dr. Bullock,

Thank you very much for your encouraging decision and helpful comments on the revised version of our manuscript, titled “PONE-D-20-26146R2: Disrupted rhythms of life, work and entertainment and their associations with psychological impacts under the stress of the COVID-19 pandemic: A Survey in 5854 Chinese people with different sociodemographic backgrounds”. We also thank the reviewer for endorsing the publication of our manuscript in PLOS ONE. We have further revised the manuscript, and highlighted all changes in the version with Track Changes. In addition, our responses to your comments, in a point-by-point format, are listed below:

**Abstract**-Method:

1. Add the add the word "also" in “correlations of SDS and SAS with SGS-scale and EPE-scale were analyzed.”

Response: We have added the word “also” in the sentence in the newly revised manuscript (Page 3, Line 16).

**Abstract**-Results:

2. Clarify the mean of “too-low or too-high” in “The scores were significantly higher in the groups with female gender, low education level, too-low or too-high income, poor health status, ages of 26-30 years or older than 61 years, nurses and subjects with divorce or widow status.”

Response: We accept your suggestion and have changed the “too-low or too-high” into “lower or higher than average” in the newly revised manuscript (Page 3, Line 21).

**Introduction**

3. delete “(Bashir, M. F. 2020)” to keep referencing style consistent throughout.

Response: We have deleted it from in the newly revised manuscript.

4. Change the word “alarming” in “Simultaneously, the COVID-19 pandemic has alarming implications for a global increase in mental health problems, and the psychological burden during the COVID-19 pandemic has been reported.” to "negative" or "significant" to avoid overstatement.

Response: We have changed it into the word “significant” in the newly revised manuscript (Page 5, Line 10).

5. Add the word “negative” before “psychological impacts” in “COVID-19-related social changes, particularly lockdown protocols, have indeed impacted the timing of daily behaviors in stabilizing circadian function, and social rhythm disruption may be correlated with psychological impacts”.

Response: We have added the word “negative” before psychological impacts in the newly revised manuscript (Page 5, Line 16).

6. Change the word “elder” to “elderly” in “Recently, several studies have reported social and circadian rhythm disruptions in students, office workers, the elder, and patients with narcolepsy or autism.”

Response: We have changed the “elder” to “elderly” in this sentence in the newly revised manuscript (Page 5, Line 20).

7. These hypotheses are not specific enough. Hypotheses should specify a direction of relationship between specific variables. I feel the "hypotheses" listed here should instead be presented as research questions which don't require as much specificity in their predictions.

Response: Thanks for kindly providing such insightful advice, which we agree and accept. Accordingly, we have replaced the hypotheses with a research question in the newly revised manuscript (Page 5, Line 21; Page 6, Line 1-3).

8. The aim should be presented before the hypotheses.

Response: According to your advice, we have replaced the hypotheses with the research question in the newly revised manuscript (Page 6, Line 3-6). Therefore, we keep the aim in the last sentence of the Introduction.

**Materials and Methods-**Participants and procedure

9. “The collection method was complied with the terms and conditions for the website.” Unclear what website is being referred to here. Do you mean the online questionnaire platform? Does that have an ethics compliance process? I'm just not sure how complying with terms and conditions for a website supports ethical practice in research. Which organisation or government body approves ethical procedures in research in China? Please clarify.

Response: We really apologize for this misleading and confusing statement, which we have deleted from the newly revised manuscript. What we wanted to express is that we complied with the terms and conditions, such as prohibition of the production, copying, publishing, and dissemination of pornographic, violent, obscene content and respect and protection of the personal privacy of all participants, which are required by the online questionnaire platform we used for the present survey study.

10. Delete the “received and” in “All completed questionnaires were received and checked for validity and completeness.”

Response: We have deleted “received and” from this sentence in the newly revised manuscript (Page 7, Line 1-2).

11. Materials and Methods-Measures

Change the word “proved” into “shown” in “The Chinese version of BSRS also included entertainment and physical practice, and was proved to have good reliability and validity in China.”

Response: We have change “proved” into “shown” in this sentence in the newly revised manuscript (Page 7, Line 8).

12.The lower the score was, the more regular the rhythm was.

Response: We have deleted the “was” in the newly revised manuscript (Page 7, Line15-16).

13. Use the word "characteristics" instead of "impacts" and the words "rated on a scale of" instead of "graded to" for clarity in “Zung's self-rating depression scale (SDS) and Zung's self-rating anxiety scale (SAS) were used to evaluate the psychological impacts of each participant[17, 18]. There were 20 items in SAS and SDS, and each item was graded to 1-4 reflecting never, often, sometimes, and always.”

Response: We have changed the words “impacts” and “graded to” into “characteristics” and “rated on a scale of”, respectively, in the newly revised manuscript (Page 7, Line 19 and Page 7, Line 20).

14. Use the word "raw" instead of "rough" here for clarity in “The rough score was the sum of 20 questions multiplied by 1.25 and rounded to get the standard score.”and explain why scores are multiplied by 1.25.

Response: We have used the “raw” instead of “rough” in this sentence in the newly revised manuscript (Page 7, Line 21). It is a common practice to convert the results to a percentage scale in the psychometric instruments. Therefore, the raw score of SAS and SDS in the present study were multiplied by 1.25 as described in previous studies [19], which has been stated in the in the newly revised manuscript (Page 7, Lines 21; Page 8, Lines 1).

*References: Sun J, Sun R, Jiang Y, Chen X, Li Z, Ma Z, et al. The relationship between psychological health and social support: Evidence from physicians in China. PLoS One. 2020;15(1):e0228152. Epub 2020/01/30. doi: 10.1371/journal.pone.0228152. PubMed PMID: 31995601; PubMed Central PMCID: PMCPMC6988930.*

15. Materials and Methods-Statistical analysis

Replace “scales of social rhythm scale”with "BSRS scales" in “Pearson correlation or Spearman correlation, where appropriate, was used to determine the correlation between the scales of social rhythm scale and the Zung’s scales.”

Response: We have replaced the “scales of social rhythm scale” with "BSRS scales" in the newly revised manuscript (Page 8, Line 16).

16. Add that statistical significance criteria were "corrected for multiple comparisons where appropriate" in “A P value of < 0.05 was considered statistically significant.”

Response: Thanks for your thoughtful suggestion. We have modified the sentence in the newly revised manuscript (Page 8, Lines 19-21) as “A P value of < 0.05 was considered statistically significant (corrected for multiple comparisons where appropriate).”

**Results**

17. Add the word “data” behind “invalid” and delete “the data analysis” in “Eighteen questionnaires with invalid were excluded the data analysis due to frivolous or incomplete answers, or the same option for different questions or orderly answers.”

Response: We have changed the sentence in the newly revised manuscript (Page 9, Lines 4) as “Eighteen questionnaires with invalid data were excluded due to frivolous or incomplete answers, or the same option for different questions or orderly answers.”

18. Adjusted for multiple comparisons? Please confirm. Some of the comparisons in Table 2 seem greater than the adjusted p value (e.g., Education, Chronic disease).

Response: Thanks for kindly providing such insightful advice. We have deleted “all” in the newly revised manuscript (Page 10, Line 7) as “Generally, there were significant differences in SGS-scale and EPE-scale among participants with different sociodemographic backgrounds including gender, age, educational background, annual income, health status, current occupation, and chronic disease status (P<0.05, Table 2).

19. An indication of effect size would help clarify the strength of these differences. Report Cohen's d statistic for all statistically significant comparisons in Tables 2, 3, and 4 to help with interpretation of the statistical outcomes.

Response: We have added a statement in the Statistical analysis section (Page 8, Lines 19-20), and reported the Cohen’s d statistic for all statistically significant comparisons in Tables 2, 3, and 4 of the newly revised manuscript.

20. Delete P<0.001 in “Additionally, SDS scores were significantly higher in medical students than in non-medical students (P<0.001).”

Response: We have deleted P<0.001 in this sentence in the newly revised manuscript (Page 14, Lines 9-10).

21. Add the “mean score” before “SGS-scale”, add the word “mean” before “SAS score” and “SDS score” in “Among the 5854 participants, SGS-scale and EPE-scale were 20.65±9.01 and 8.12±6.00, respectively, and SAS score and SDS score were 30.51±7.49 and 41.95±11.75, respectively.”

Response: We have modified this sentence in the newly revised manuscript (Page 16, Lines 6-7) as “Among the 5854 participants, mean scores on the SGS-scale and EPE-scale were 20.65±9.01 and 8.12±6.00, respectively, and mean SAS score and mean SDS score were 30.51±7.49 and 41.95±11.75, respectively. ”

**Discussion**

22. “lower or higher than average income” needs to be defined better. See my comment in the Abstract.

Response: We have changed it into “lower or higher than average income” in the newly revised manuscript (Page 18, Line 18-19; Page 19, Line 1-2).

23. Which hormones? I assume you mean female-specific hormones? Please clarify. “The endogenous circadian clocks can be reset by hormone signals, affecting the balance of the hypothalamus-pituitary-adrenal (HPA) axis, and making the HPA axis more responsive to stress than males.”

Response: Thank you for your kind suggestion. We have clarified this issue and modified the sentence in the newly revised manuscript (Page 19, Lines 7) as follow: “The endogenous circadian clocks can be reset by estrogen hormone signals in women, affecting the balance of the hypothalamus-pituitary-adrenal (HPA) axis, and making the HPA axis more responsive to stress than males.”

24. “likely due to their weak competitiveness in the job market and inability to adapt to the new E-commerce models during the COVID-19 pandemic due to the isolation or even lockdown.” A reference is needed to support this statement.

Response: A reference has been added to support this statement in the newly revised manuscript (reference [26], Page 19, Line 19)

*Reference: Brenner MH, Bhugra D. Acceleration of Anxiety, Depression, and Suicide: Secondary Effects of Economic Disruption Related to COVID-19. Frontiers in psychiatry. 2020;11:592467. Epub 2021/01/02. doi: 10.3389/fpsyt.2020.592467. PubMed PMID: 33384627; PubMed Central PMCID: PMCPMC7771384.*

25. Use the word "timely" instead of "timeless" to help with clarity. “Moreover, they did not have sufficient judgment while facing various, overloaded and timeless information on the COVID-19 pandemic, and easily felt stressed, depressive and anxious[27].”

Response: We have used the word "timely" instead of "timeless" in the newly revised manuscript (Page 19, Line 20).

26. Please re-write this highlighted section to improve clarity. It is unclear at the moment why people with high income are also the people with poor health status.- “Interestingly, in the present study, the people with high income were among those who mostly suffered from disrupted rhythms of entertainment during the COVID-19 pandemic. It has been shown that people with poor health status are likely to have basic diseases, weak immune function, and low ability to fight pathogens or adapt to a rhythm change[26], which may explain that people with poor health status suffered from disruption of social rhythm observed in the represent study.”

Response: Thank you for your thoughtful suggestion. We have rewritten this section to clarify the disrupted rhythm characteristics of different groups of people in the newly revised manuscript (Page 20, Lines 1-13) as follows: “Interestingly, both the people with high incomes and those with poor health status were mostly suffered from disrupted rhythms. On one hand, the disrupted rhythm in people with high incomes may be explained as follows: during the COVID-19 pandemic, the consumption expenditures of high-income groups dropped sharply by as much as 17%, while the expenditures of low-income families dropped by only 4%[28]. At the same time, entertainment venues that provide services for high-income groups, such as cultural activity centers, clubs and gymnasiums, golf playgrounds, luxury party venues, were mostly closed. All these changes would impact severely on the social rhythms of these groups of people. On the other hand, the disrupted rhythm in people with poor health status can be explained by the fact that their regular activities, such as walking, jogging, swimming and fitness, were significantly restricted since their poor health status with basic diseases, weak immune function, and low ability to fight pathogens or adapt to a rhythm change[29].

27. Correct the spelling in “Previously, Liu et al. [29]conducted a study on lifestyle and the health status of Chinee elderly, which included the elderly who lived in their own houses, with 20.6% of them being widows or widowers.”

Response: We have corrected the spelling of the word “Chinese” in the newly revised manuscript (Page 21, Line 5).

28. Has to be linked back to sleep - how does decreasing amplitude and phase advance of the circadian rhythm affect sleep. “Indeed, it has been reported that advanced age is characterized by a progressive decreasing amplitude and phase advance of circadian rhythmicity in overall biological functions,”

Response: Actually, this paragraph intends to address the reasons how COVID-19 pandemic impacted on the Chinese elderly through various internal and external factors. Therefore, we modified this section in the newly revised manuscript (Page 21, Lines 9-16) as follows “Indeed, it has been reported that advanced age is characterized by a progressive decreasing amplitude and phase advance of circadian rhythmicity in overall biological functions, including blood pressure, hormonal circadian secretions, and immune function changes[33]. The elderly could not go out for dancing after dinner, exercise in the morning, or visit old friends or relatives due to recommended social isolation. Furthermore, they were inherently at high risk for COVID-19 infection and easily generated negative emotions [34]. These various factors were prone to form a vicious circle.”

29. Deleted “that offers an explanation” in “The first factor is the social zeitgeber theory that offers an explanation. ”

Response: We have deleted it in this sentence in the newly revised manuscript (Page 22, Line 16 ).

30. Add "outcomes" after "mental health" in “Social stimuli under the stress of COVID-19 may affect the circadian behavioral programs by regulating the phase and period of circadian clocks (i.e. a zeitgeber action, either direct or by conditioning to photic zeitgebers) which leads to various adverse mental health.”

Response: We have added outcomes after “mental health” in this sentence in the newly revised manuscript (Page 23, Line 2).

31. I don't see this as a strong enough justification or explanation for the findings, especially as the reference source is based on studies in mice. Either drop this justification altogether or provide a stronger argument supported by more relevant research to humans. The third factor is SCN that serves as a master clock synchronizing the phase of clocks throughout the body. The disrupted rhythm in SCN leads to helplessness and behavioral despair, which increases depression and anxiety-related behaviors[36].

Response: We have deleted this point of view in the newly revised manuscript.

32. Add the word “respectively” after “social stress” to improve clarity in “Anxiety disorders and major depressive disorder have been associated with increased and blunted HPA axis reactivity to social stress, which engages the diverse biological pathways that bolster successful stress adaptation and promote stress resilience.”

Response: We have added the word “respectively” after “social stress” in this sentence in the newly revised manuscript (Page 23, Line 14).

33. Please correct the spelling in “Of course, other mechanisms may also connect social rhythm dysregulation with phycological impacts.” and add “, including circadian gene variations [39] and social jetlag [40].” at the end of this sentence. Then delete the section “A previous study reported that the 4/4 genotype of the circadian gene, PER3, was associated with anxiety symptoms in 804 Brazilian non-clinical young adults[39]. Mathew and his colleagues also found that social jetlag (a misalignment between sleep timing on the weekend and school week) was positively and independently associated with anxiety symptoms in adolescents[40].”

Response: We have modified this sentence as “Of course, other mechanisms may also connect social rhythm dysregulation with psychological impacts, including circadian gene variations [43] and social jetlag [44].” in the newly revised manuscript (Page 23, Line 19-20).

34. Please add a supporting reference in “2), spending 1 hour/day outdoors, walking for more than one hour a day and basking in the sun, which may regulate the body clock by the light-dark cycle;”.

Response: Thank you for your important advice. A supporting reference has been added in the newly revised manuscript (Page 24, Line 9) .

*Reference 7, Murray G, Gottlieb J, Swartz HA. Maintaining Daily Routines to Stabilize Mood: Theory, Data, and Potential Intervention for Circadian Consequences of COVID-19. Can J Psychiatry. 2020:706743720957825. Epub 2020/09/11. doi: 10.1177/0706743720957825. PubMed PMID: 32909832.*

35. “Previous studies have shown that regular eating and consumption of various metabolites promote the periodic colonization of the intestinal bacteria on the mucosal surface, and alleviate various adverse mental problems through the intestinal flora-gut-brain axis[41];”- This isn't a circadian rhythm explanation - it deviates too far from the central themes of the paper and should be removed.

Response: We have deleted this sentence in the newly revised manuscript.
